# Supplementary material for: Cost-effectiveness of Spironolactone for Adult Female Acne (SAFA): economic evaluation alongside a randomised controlled trial
Source: BMJ Open. 2023 Dec 10;13(12):e073245. doi: 10.1136/bmjopen-2023-073245 (PMC10729081; doi:10.1136/bmjopen-2023-073245)
Supplement: Supplementary data [file bmjopen-2023-073245supp002.pdf]

SUPPORTING INFORMATION

ONLINE SUPPLEMENTAL FILE S2: PARTICIPANT RESOURCE USE QUESTIONNAIRE

The following information is presented in addition to the main paper, "Cost-effectiveness of Spironolactone for Adult Female Acne (SAFA): Economic evaluation alongside a randomised controlled trial", cited as Pyne S, Sach TH, Lawrence M, et al *BMJ Open* 2023;:1–11. doi: bmjopen-2023-073245.

The example given below is taken from the SAFA 6-week questionnaire. These questions were part of a wider questionnaire used at 6 weeks.

Participant's initials: ☐☐☐ Participant's study identifier: ☐☐☐☐☐☐☐☐

SAFA 6 week Questionnaire – Participant

Services received

These questions are about your health and care needs. In the **last 6 weeks** what publically provided services (i.e. those you do not have to pay for out of your own pocket) have you received because of your acne?

If you are unsure, please put in your best estimate.

Question 1: Community-based NHS services

1a. In the **last 6 weeks** have you seen any community-based health professionals (e.g. GP, practice nurse, dietician etc) because of your acne?

☐ Yes ☐ No, if 'No' please go to question 2

Have you seen any of the following health professionals in the **last 6 weeks**? If 'Yes', please tell us how many times. There is space for you to name other professionals you have seen via the NHS and how many time you have visited them. If you did not see any other professionals please tick 'No' in the "Other" rows.

General Practitioner	☐ No	☐ yes	If yes, how many times?	___________
Practice nurse	☐ No	☐ Yes	If yes, how many times?	___________
Health care assistant	☐ No	☐ Yes	If yes, how many times?	___________
NHS Walk in centre	☐ No	☐ Yes	If yes, how many times?	___________
Community dermatology service	☐ No	☐ Yes	If yes, how many times?	___________
Other, please specify: _________________________	☐ No	☐ Yes	If yes, how many times?	___________
Other, please specify: _________________________	☐ No	☐ Yes	If yes, how many times?	___________
Other, please specify: _________________________	☐ No	☐ Yes	If yes, how many times?	___________

Funded by NIHR

Participant's initials: | | | |

Participant's study identifier: | | | | | | | | |

Question 2: Medication

2. In the **last 6 weeks** have you been **prescribed** any medications because of your acne? (Please include anything that you feel is related to your acne, for example if you take anti-depressants and your depression is mainly because of your acne you would include this).

☐ Yes ☐ No, if 'No' please go to question 3a

If 'Yes', please give the name of the medication, the strength, and size of the item.

Name of medication (item)	Strength	Unit	Number of items	Type of item (e.g. pack, bottle, tube, etc)	Number in item	Size of item
Example 1: Epiduo Gel	2.5	%	2	tubes	12	grams per tube
Example 2: Tetracyclin	250	mg	1	pack	28	tablets per pack

Question 3: Hospital-based services

3a. **In the last 6 weeks** have you visited a hospital as an outpatient because of your acne or side effects from treatment for your acne?

☐ Yes ☐ No, if 'No' please go to question 3b

If 'Yes', for **each outpatient visit** you had at the hospital as a result of your acne, please tell us which health professional you saw and how many times. Please enter '0' if you did not visit the health professional or in 'Other' if there were no other visits.

Please do not include visits with any professionals that took place outside of the hospital. These should be included in question 1 above. Please do not include visits made as part of this study in your answers below.

Funded by NIHR

Participant's initials: ☐☐☐ Participant's study identifier: ☐☐☐☐☐☐☐☐

**Health professional you saw** *(If unknown, please write the department in which you saw them)*	**Number of outpatient visits**
Example: Dermatology nurse	*2* visits
Dermatologist	visits
Dermatology nurse	visits
Other, please specify:	visits
Other, please specify:	visits
Other, please specify:	visits

3b. Did you attend Accident and Emergency Services in the **last 6 weeks** because of your acne or side effects from treatment for your acne?

☐ Yes ☐ No, if 'No' please go to question 3c

If 'Yes', how many visits in the last 6 weeks: ______________________________

3c. In **the last 6 weeks**, have you been admitted to hospital as an inpatient as a result of your acne or side effects from treatment for your acne?

☐ Yes ☐ No, if 'No' please go to question 4

If 'Yes', for **each inpatient** visit you have had, please tell us the type of ward you were admitted to and for how many nights.

Please include any day case procedures

**Visit number**	**The type of department or ward or reason for admission**	**Duration of each stay (number of nights)**
Example	*Dermatology*	*2* nights
1		nights
2		nights

Participant's initials: | | | |

Participant's study identifier: | | | | | | | | |

Question 4: Other services

4. In the **last 6 weeks** have you received any other publically provided services because of your acne?

☐ Yes ☐ No, if 'No' please go to question 5a

If 'Yes', please give details including type and how many times received:

Details of service	Type of service	Number of times received

Costs incurred by yourself or family

These next few questions are about the costs incurred by you and your family/friends because of your acne.

Question 5: Personal Costs

5a. In the **last 6 weeks** have you or your family/friends incurred any other costs because of your acne? Please do not include visits made as part of this study in your answers below.

☐ Yes ☐ No, if 'No' please go to question 5b

If 'Yes', please give the details below and the approximate cost of items purchased as a result of your acne.

Item	Number of items or visits	Overall cost
Example: Homeopath	*2 visits*	*£80 (2 x £40)*
Complementary therapists		
Non-prescribed medication		
Travel costs to health care appointments		
Parking costs at health care appointments		
Cosmetic and skin care products		
Other, please specify:		

Funded by NIHR

Participant's initials: ☐☐☐ Participant's study identifier: ☐☐☐☐☐☐☐☐

Other, please specify:		
Other, please specify:		

5b. What is your current **primary occupation**? Please tick one:

☐ Paid employment ☐ self-employment ☐ Voluntary work ☐ Education/studying

☐ None of the above (i.e. retired, unemployed)

In the **last 6 weeks** has your acne had an impact on your primary occupation?

☐ Yes ☐ No, if 'No' please go to question 5c.

If 'Yes', please fill in each row of the table below **about how your acne has affected your primary occupation** in the last 6 weeks. This asks only about your acne, so if, for example, you reduced your hours worked to look after a dependent please do not put this in this table. Please do not include visits made as part of this study in your answers below.

I have had to take leave	☐ No	☐ Yes	If yes, how much leave have you taken in the last 6 weeks?<br>_____weeks ________days _______hours<br>If in paid employment or self-employment, was this paid leave?<br>☐ Yes ☐ No ☐ Mixture of paid and unpaid<br>If a mixture of paid and unpaid leave, how much of the leave was paid leave?<br>_____weeks ________days _______hours
I have reduced the hours I undertake my primary occupation each week	☐ No	☐ Yes	If yes, how many hours per week did you used to undertake?<br>How many hours per week do you undertake now?<br>How long ago did this change:<br>_____weeks ________days _______hours

Funded by NIHR

Participant's initials: | | | |

Participant's study identifier: | | | | | | | | |

I have increased the hours I undertake my primary occupation each week	☐ No	☐ Yes	If yes, how many hours per week did you used to undertake?<br><br>How many hours per week do you undertake now?<br><br>How long ago did this change:<br>_____weeks ________days _______hours
I have completely stopped my primary occupation and will not be going back to it	☐ No	☐ Yes	How long ago did this change:<br>_____weeks ________days _______hours
I have changed my role within my primary occupation	☐ No	☐ Yes	If yes, what was your old role title:<br><br>What is your new role title:<br><br>How long ago did this change:<br>_____weeks ________days _______hours

5c. Have you had a family member or friend who has had to take time off paid work to accompany you to health care appointments related to your acne?

☐ Yes ☐ No, if 'No' please go to question 5d

If yes, how much leave have they had to take in the last 6 weeks to accompany you to appointments related to your acne?

________hours

5d. **Support outside of official services** (For example, charity support groups such as The Acne and Rosacea Association, helplines etc)

In the **last 6 weeks**, have you received support or attended support groups?

☐ Yes ☐ No

If 'Yes', please list what support you have accessed and state whether you incurred any costs as a result (e.g. membership fee, participation fee, telephone cost etc)

Funded by NIHR

Participant's initials: | | | |

Participant's study identifier: | | | | | | | | |

Type of Support	Cost Incurred (£)
	£
	£
	£

Thank you for completing this questionnaire.

Funded by NIHR
