# Supplementary material for: Cost-effectiveness of Spironolactone for Adult Female Acne (SAFA): economic evaluation alongside a randomised controlled trial
Source: BMJ Open. 2023 Dec 10;13(12):e073245. doi: 10.1136/bmjopen-2023-073245 (PMC10729081; doi:10.1136/bmjopen-2023-073245)
Supplement: Supplementary data [file bmjopen-2023-073245supp001.pdf]

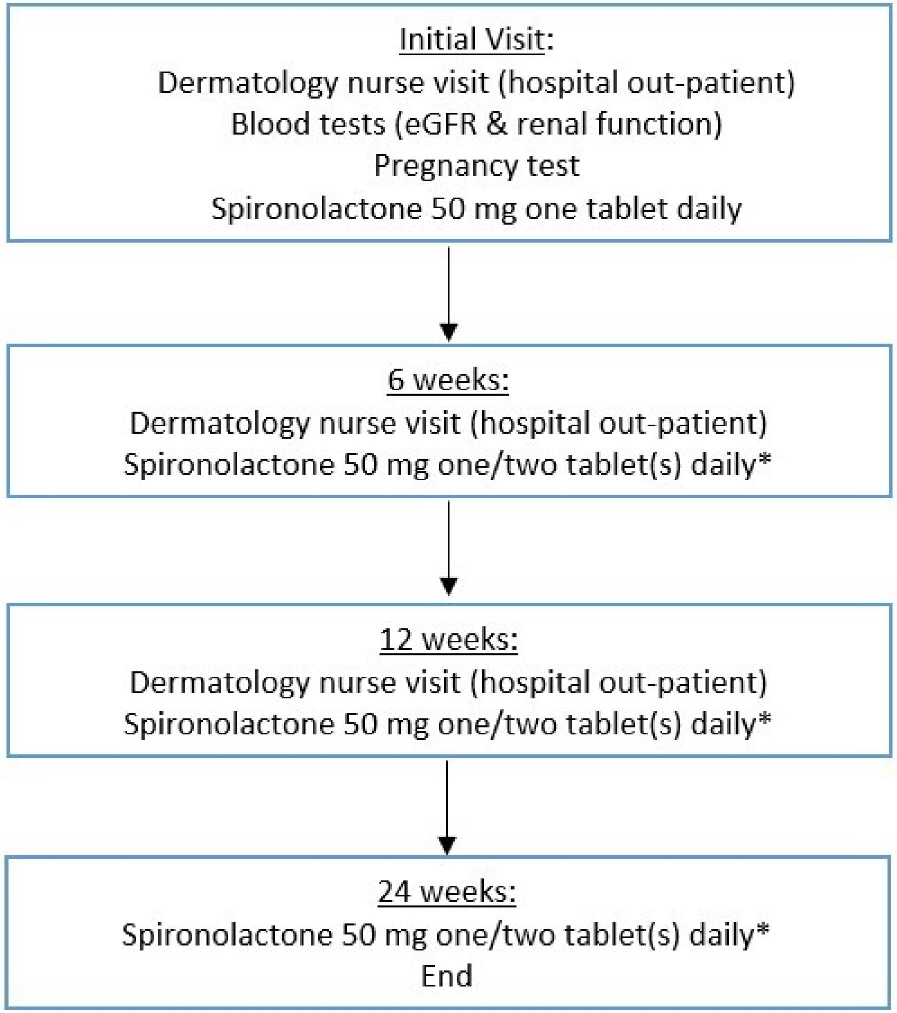

*At 6 weeks, dose was increased to 100 mg/day, assuming treatment was tolerated, which was the case for 182/184 (99%) of available patients in the spironolactone arm of the study
