# Supplementary material for: Cost-effectiveness of Spironolactone for Adult Female Acne (SAFA): economic evaluation alongside a randomised controlled trial
Source: BMJ Open. 2023 Dec 10;13(12):e073245. doi: 10.1136/bmjopen-2023-073245 (PMC10729081; doi:10.1136/bmjopen-2023-073245)
Supplement: Supplementary data [file bmjopen-2023-073245supp003.pdf]

SUPPORTING INFORMATION

ONLINE SUPPLEMENTAL FILE S3: FURTHER SENSITIVITY AND SUBGROUP ANALYSES

The following information is presented in addition to the main paper, "Cost-effectiveness of Spironolactone for Adult Female Acne (SAFA): Economic evaluation alongside a randomised controlled trial", cited as Pyne S, Sach TH, Lawrence M, et al *BMJ Open* 2023;1–11. doi: bmjopen-2023-073245 and linked to the clinical trial paper published in The BMJ.[1] In addition to the sensitivity analyses presented in the main paper, a further two sensitivity analyses and a sub-group analysis were agreed before analysis and conducted to explore key uncertainties around the parameters of the economic evaluation. The details of these are outlined below.

Baseline Resource use
Table S1 presents the levels of resource use, at baseline, prior to randomisation (Table S1).

Sensitivity analysis: costing the intervention as per the SAFA trial protocol

Figure S1 describes the per protocol intervention resource use, undertaken in the trial and used to inform sensitivity analysis 2 (SA2). Subgroup analysis by age
A single sub-group analysis was undertaken for age (categorised as below 25 years and 25 years and over) as the clinical analysis found age significantly interacts with the outcome.[1]

The ICER was £263,871 per QALY for women under 25 years compared to £19,994 for women over 25 years of age (see Table S2). This result suggests that spironolactone is likely to be cost effective for women aged over 25 years. Whilst this finding is in line with the clinical findings, it ought to be interpreted with caution given the small sample sizes necessitated by splitting the dataset into subgroups combined with missing data.

Costs and outcomes over 52 weeks
Data was also collected beyond the treatment period (24 weeks) for up to 52 weeks. Response rates were, however, significantly lower at this time point, with 58% of participants missing EQ-5D data and 93% missing resource use data (see Supplementary Table S3). It is difficult to draw conclusions from these data, but incremental QALYs over 52 weeks was 0.0644 (95%CI 0.0093 to 0.1194) and incremental cost (NHS perspective) (see Supplementary Table S4) over the same period was £95.44 (95% CI 8.29 to 182.70).

Reference:

1. Santer M, Lawrence M, Renz S, *et al.* Effectiveness of spironolactone for women with acne vulgaris (SAFA) in England and Wales: pragmatic, multicentre, phase 3, double blind, randomised controlled trial. *BMJ* 2023;**BMJ-2022-074349**:e074349. doi:10.1136/bmj-2022-074349

SUPPLEMENTARY FIGURES

Supplementary Figure S1 | Intervention resource use as delivered via secondary care per trial protocol

SUPPLEMENTARY TABLES

Supplementary Table S1 | Estimates of mean baseline resource use by treatment group (available case data)

Resource	Spironolactone (N=201)		No active systemic treatment (N=209)		Mean difference
	Mean (n)	Std dev	Mean (n)	Std dev	(95% CI)
Total community-based HCP visits	0.27 (200)	0.616	0.225 (209)	0.590	0.045 (-0.072 to 0.162)
Total hospital contacts	0.119 (193)	0.446	0.095 (200)	0.396	0.024 (-0.059 to 0.108)
All medications – quantity (number)	11.711 (201)	46.065	7.903 (206)	21.570	3.809 (-3.174 to 10.791)
Total out-of-pocket items	2.027 (188)	2.735	1.939 (196)	2.438	0.088 (-0.432 to 0.607)
Lost patient work time (number reporting)	0.020 (197)	0.141	0.034 (205)	0.182	-0.014 (-0.046 to 0.018)
Lost carer work time (number reporting)	0.015 (194)	0.124	0.030 (203)	0.170	-0.014 (-0.044 to 0.015)

Supplementary Table S2 | Cost utility analyses and cost-effectiveness analyses results, for additional sub-group analysis

CUA Analysis (N s, N p)	Incremental cost (95% CI)	Incremental QALYs (95% CI)	ICER	CEAC at £20,000 (£30,000) threshold*
Sub-group analysis: <25 years, CCA, adjusted: (28,29)	108.23 (89.09 to 127.37)	0.0004 (-0.0141 to 0.0150)	£263,871	25% (33%)
Sub-group analysis: ≥25 years, CCA, adjusted: (90,72)	133.06 (114.97 to 151.16)	0.0067 (-0.0079 to 0.0213)	£19,994	50% (62%)

95% CI=95% confidence interval; ICER =incremental cost-effectiveness ratio; N s / N p =Number randomised to spironolactone / Placebo who were included in the analysis; CCA = complete case analysis; SA refers to the different sensitivity analyses described in the Methods; QALY=Quality Adjusted Life Years; *probability of being cost-effective at a the threshold (λ) of £20,000 and £30,000 per QALY. Adjusted analyses, adjusted for stratification variables (centre, baseline severity [IGA<3 vs. ≥3]) and baseline variables (Acne QoL symptom subscale score, use of topical treatments, utility score based on EQ-5D, total costs)

Supplementary Table S3 | 1Proportion of Missing values (%) for key variables

Variable	Spironolactone	No active systemic treatment	Total
**Baseline variables**			
Treatment allocation	0	0	0
Centre	0	0	0
Baseline severity (IGA)	0	0	0
Acne-QoL symptom subscale score at baseline	0	0	0
Use of topical treatments (y/n)	1.00	0.48	0.73
EQ-5D at baseline	0.50	0.00	0.24
Costs at baseline	4.48	5.74	5.12
**Cost variables***			
Costs at 6 weeks	17.91	18.18	18.05
Costs at 12 weeks	14.43	23.44	19.02
Costs at 24 weeks	23.88	38.76	31.46
Costs at 52 weeks	92.54	94.26	93.41
**Outcome variables for health-related quality of life**			
EQ-5D at 6 weeks	12.44	14.35	13.41
EQ-5D at 12 weeks	13.43	20.57	17.07
EQ-5D at 24 weeks	20.40	33.49	27.07
EQ-5D at 52 weeks	54.73	61.72	58.29
**Outcome variables for Acne-related quality of life**			
Acne-QoL at 6 weeks	12.44	14.35	13.41
Acne-QoL at 12 weeks	12.44	20.57	16.59
Acne-QoL at 24 weeks	18.91	34.93	27.07
Acne-QoL at 52 weeks	52.74	61.24	57.07
**Outcomes for cost-utility and cost-effectiveness analyses***			
Total costs (treatment period)	36.32	47.38	41.95
Total QALYS (treatment period)	20.90	33.49	27.32
Change Acne-QoL symptoms (treatment period)	18.91	34.93	27.07

Treatment period = baseline to 24 weeks

*For base-case, i.e. NHS-related costs only

Supplementary Table S4 | Mean (Standard Deviation) Cost and Cost Difference (95% Confidence Interval) Per Patient up to 25-–52 weeks for the Intervention arm compared to usual care arm (in 2021 UK pounds sterling)

Resource	Spironolactone (N=201)		No active systemic treatment (N=209)		Mean difference
	Mean (n)	Std dev	Mean (n)	Std dev	(95% CI)
**Costs**					
All community-based HCP costs	19.64 (16)	33.25	33.24 (13)	42.00	-13.60 (-42.25 to 15.05)
Total hospital contacts cost	17.10 (15)	45.13	9.87 (13)	35.57	7.23 (-24.70 to 39.17)
All medication cost	4.81 (16)	11.23	9.66 (13)	19.41	-4.85 (-16.65 to 6.96)
Total costs (NHS perspective), 25–52 weeks	39.89 (15)	67.47	54.41 (12)	79.00	-14.52 (-72.57 to 43.52)
Total costs (NHS perspective), 0–52 weeks	179.21 (13)	76.99	83.76 (10)	123.54	95.44 (8.29 to 182.60)
**Outcomes**					
52 weeks EQ-5D-5L utility	0.9208 (92)	0.1516	0.8291 (79)	0.2664	0.0918 (0.0274 to 0.1561)
QALYs at 52 weeks	0.9158 (88)	0.1364	0.8515 (74)	0.2154	0.0644 (0.0093 to 0.1194)
52 weeks symptom Acne-QoL	21.634 (95)	6.257	19.963 (81)	5.697	1.671 (-0.122 to 3.464)
Symptom Acne QoL change at 52 weeks compared to baseline	8.613 (95)	7.154	6.951 (81)	6.500	1.663 (-0.385 to 3.710)
