# Supplementary material for: Cost-effectiveness of Spironolactone for Adult Female Acne (SAFA): economic evaluation alongside a randomised controlled trial
Source: BMJ Open. 2023 Dec 10;13(12):e073245. doi: 10.1136/bmjopen-2023-073245 (PMC10729081; doi:10.1136/bmjopen-2023-073245)
Supplement: Author's manuscript [file bmjopen-2023-073245.draft_revisions.pdf]

BMJ Open

BMJ Open is committed to open peer review. As part of this commitment we make the peer review history of every article we publish publicly available.

When an article is published we post the peer reviewers' comments and the authors' responses online. We also post the versions of the paper that were used during peer review. These are the versions that the peer review comments apply to.

The versions of the paper that follow are the versions that were submitted during the peer review process. They are not the versions of record or the final published versions. They should not be cited or distributed as the published version of this manuscript.

BMJ Open is an open access journal and the full, final, typeset and author-corrected version of record of the manuscript is available on our site with no access controls, subscription charges or pay-per-view fees (http://bmjopen.bmj.com).

If you have any questions on BMJ Open's open peer review process please email info.bmjopen@bmj.com

Cost-effectiveness of Spironolactone for Adult Female Acne (SAFA): Economic evaluation alongside a randomised controlled trial

Journal:	*BMJ Open*
Manuscript ID	bmjopen-2023-073245
Article Type:	Original research
Date Submitted by the Author:	28-Feb-2023
Complete List of Authors:	Pyne, Sarah; University of East Anglia, Health Economics Group, Norwich Medical School<br>Sach, Tracey; University of East Anglia, Health Economics Group, Norwich Medical School<br>Lawrence, Megan; University of Southampton<br>Renz, Susanne; University Hospital Southampton NHS Foundation Trust<br>Eminton, Zina; University Hospital Southampton NHS Foundation Trust<br>Stuart, Beth; Queen Mary University of London, Centre for Evaluation and Methods Wolfson Institute of Population Health<br>Thomas, Kim; University of Nottingham, Dermatology<br>Francis, Nick; University of Southampton, Primary Care Research Centre<br>Soulsby, Irene; University of Southampton Faculty of Medicine, Primary Care and Population Sciences<br>Thomas, Karen ; University of Southampton, Public contributor, Primary Care Research Centre<br>Permyakova, Natalia; University of Southampton; University Hospital Southampton NHS Foundation Trust<br>Ridd, Matthew; University of Bristol, Population Health Sciences<br>Little, Paul; University of Southampton<br>Muller, Ingrid; University of Southampton<br>Nuttall, Jacqui; University of Southampton<br>Griffiths, Gareth; University of Southampton<br>Layton, Alison M; University of York, Skin Research Centre, Hull York Medical School<br>Santer, Miriam; University of Southampton, Primary Medical Care
Keywords:	Clinical Trial, Dermatology < INTERNAL MEDICINE, Health economics < HEALTH SERVICES ADMINISTRATION & MANAGEMENT, HEALTH ECONOMICS, Acne < DERMATOLOGY, Adult dermatology < DERMATOLOGY

SCHOLARONE™
Manuscripts

I, the Submitting Author has the right to grant and does grant on behalf of all authors of the Work (as defined in the below author licence), an exclusive licence and/or a non-exclusive licence for contributions from authors who are: i) UK Crown employees; ii) where BMJ has agreed a CC-BY licence shall apply, and/or iii) in accordance with the terms applicable for US Federal Government officers or employees acting as part of their official duties; on a worldwide, perpetual, irrevocable, royalty-free basis to BMJ Publishing Group Ltd ("BMJ") its licensees and where the relevant Journal is co-owned by BMJ to the co-owners of the Journal, to publish the Work in this journal and any other BMJ products and to exploit all rights, as set out in our licence.

The Submitting Author accepts and understands that any supply made under these terms is made by BMJ to the Submitting Author unless you are acting as an employee on behalf of your employer or a postgraduate student of an affiliated institution which is paying any applicable article publishing charge ("APC") for Open Access articles. Where the Submitting Author wishes to make the Work available on an Open Access basis (and intends to pay the relevant APC), the terms of reuse of such Open Access shall be governed by a Creative Commons licence – details of these licences and which Creative Commons licence will apply to this Work are set out in our licence referred to above.

Other than as permitted in any relevant BMJ Author's Self Archiving Policies, I confirm this Work has not been accepted for publication elsewhere, is not being considered for publication elsewhere and does not duplicate material already published. I confirm all authors consent to publication of this Work and authorise the granting of this licence.

For peer review only

TITLE:

Cost-effectiveness of Spironolactone for Adult Female Acne (SAFA): Economic evaluation alongside a randomised controlled trial

manuscript word count: 3,084 Words
Table count: 3
Figure count: 2

AUTHORS:

Sarah Pyne,[1] Tracey H Sach*,[1] Megan Lawrence,[2] Susanne Renz,[2] Zina Eminton,[2] Beth Stuart,[3,4] Kim S Thomas,[5] Nick Francis,[4] Irene Soulsby,[6] Karen Thomas,[6] Natalia Permyakova,[2] Matthew J Ridd,[7] Paul Little,[4] Ingrid Muller,[4] Jacqueline Nuttall,[2] Gareth Griffiths,[2] Alison Layton,[8] Miriam Santer,[4]

[1]Health Economics Group, Norwich Medical School, University of East Anglia, Norwich, UK

[2]Southampton Clinical Trials Unit, University of Southampton and University Hospital Southampton NHS Foundation Trust, Southampton, UK

[3]Centre for Evaluation and Methods, Wolfson Institute of Population Health, Faculty of Medicine and Dentistry, Queen Mary University of London, London, UK

[4]Primary Care Research Centre, School of Primary Care, Population Sciences and Medical Education, Faculty of Medicine, University of Southampton, Southampton, UK

[5]Centre of Evidence Based Dermatology, School of Medicine, University of Nottingham, Nottingham, UK

[6]Public contributor, UK

[7]Population Health Sciences, Bristol Medical School, University of Bristol, Bristol, UK

[8]Skin Research Centre, Hull York Medical School, University of York, York, UK

***Corresponding author:** Professor Tracey Sach, Health Economics Group, Norwich Medical School, University of East Anglia, Norwich Research Park, Norwich NR4 7TJ. Email: T.sach@uea.ac.uk

FUNDING SOURCES

This study presents independent research funded by the National Institute for Health and Care Research (NIHR) under its Health Technology Assessment programme (16/13/02). The views expressed are those of the author(s) and not necessarily those of the NHS, the NIHR or the Department of Health and Social Care.

This trial was registered prospectively with the ISRCTN registry (ISRCTN12892056) and EudraCT (2018-003630-33).

For peer review only - http://bmjopen.bmj.com/site/about/guidelines.xhtml

CONFLICTS OF INTEREST

We declare no support from any organisation other than the NIHR for the submitted work; no financial relationships with any organisations that might have an interest in the submitted work; no other relationships or activities that could appear to have influenced the submitted work.

LH has received consultancy fees from the University of Oxford on an educational grant funded by Pfizer, unrelated to the submitted work. THS was a member of NIHR HTA Efficient Study Designs - 2, HTA Efficient Study Designs Board, HTA End of Life Care and Add-on-Studies, HTA Primary Care Themed Call Board and the HTA Commissioning Board between 2013 to Dec 2019. She is a steering committee member of the UK Dermatology Clinical Trials Network and Chair of the NIHR Research for Patient Benefit Regional Advisory Panel for the East of England. THS had no part in the decision making for funding this study.

STRENGTHS AND LIMITATIONS

- Our study is based on individual patient level data collected alongside a large pragmatic, parallel, double-blind, randomised trial.
- The base case set out to answer the question of whether spironolactone is cost-effective compared with no active systemic treatment (both groups could use routine topical medications) to align with the clinical question funded. In practice, clinicians are unlikely to send women away with no active treatment if they consulted with acne persisting beyond 6 months. We undertook sensitivity analysis to explore the impact of comparing to standard of care (oral antibiotics).
- There was differential rates of missing data over time between groups. We addressed this by undertaking both a complete case analysis and multiple imputation to explore the impact of missing data on the study conclusions.
- We also undertook sensitivity analysis to assess the impact on our findings of a wider perspective (including participant and family out of pocket and productivity costs) and delivery of the intervention in a different setting (secondary care as opposed to primary care).
- A sub-group analysis estimates the cost-effectiveness of spironolactone for those aged above and below age 25 years.

For peer review only - http://bmjopen.bmj.com/site/about/guidelines.xhtml

ABSTRACT (300/300 WORDS)

Background

Acne is common, can cause significant negative impacts and is frequently treated with prolonged courses of oral antibiotics. Despite little clinical and economic evidence spironolactone is prescribed for persistent acne in adult women.

Objective

To estimate the cost effectiveness of oral spironolactone for persistent acne in adult women from an NHS perspective over 24-weeks.

Design

Economic evaluation undertaken alongside a pragmatic, parallel, double-blind, randomised trial.

Setting

Primary and secondary healthcare, community and social media advertising.

Participants

Women ≥18 years with persistent facial acne judged to warrant oral antibiotic treatment.

Interventions

Participants were randomised 1:1 to 50 mg/day spironolactone (increasing to 100mg/day after 6 weeks) or matched placebo until week-24. Participants in both groups could continue topical treatment use.

Main outcome measures

Cost utility analysis assessed incremental cost per Quality-Adjusted Life Year (QALY) using the EQ-5D-5L. Secondary cost effectiveness analysis estimated incremental cost per unit change on the Acne-QoL symptom subscale.

Results

Spironolactone did not appear cost-effective in the complete case analysis (n=126 spironolactone and n=109 control), compared with placebo (adjusted incremental cost per QALY £67,191; unadjusted £34,770). Incremental cost per QALY was £27,879 (adjusted), just below the upper NICE threshold value, where multiple imputation took account of missing data. Sensitivity analysis comparing spironolactone to oral antibiotic control gave an adjusted incremental cost per QALY of £9,169. The adjusted incremental cost per point change on the Acne-QoL symptom subscale for spironolactone compared to placebo was £38.21 (complete case analysis).

Conclusions

Compared to placebo (no active systemic treatment), spironolactone was estimated to be marginally cost-effective where multiple imputation was performed but cost-ineffective in complete case analysis. Participants entering the trial all required systemic treatment, making standard of care (oral antibiotics) a more appropriate comparator; against which cost-effectiveness of spironolactone appears favourable, and avoids wider societal costs of over-use of antibiotics.

For peer review only - http://bmjopen.bmj.com/site/about/guidelines.xhtml

For peer review only

INTRODUCTION

Acne (acne vulgaris) is a common condition, affecting >80% of people at some point in their life.[1] Its impact on the NHS is considerable, being responsible for around 3.5 million consultations with a GP [1] and 70,000 referrals for specialist care[2] in the UK annually. As well as direct burdens to the NHS, adults (18–30 years) with severe acne in the UK have higher unemployment rates[3] and a small study by Jowett and Ryan (1985)[4] showed that 45% (13/29) of acne patients reported interpersonal difficulties at work.

The National Institute of Health and Care Excellence (NICE) guidelines on the management of acne vulgaris recommend a fixed combination topical preparation containing retinoids, benzoyl peroxide or antibiotics as first-line treatment for mild-to-moderate acne, whilst a fixed combination topical agent plus oral lymecycline or doxycycline once daily is recommended for moderate-to-severe acne.[5] The guidance states that treatment options including an antibiotic (topical or oral) should only be continued for more than 6 months in exceptional circumstances (other guidelines limit oral antibiotic duration to 3 months)[6–8] and that clinicians should be aware of the associated risks of antimicrobial resistance. Doctors, however, report many challenges when trying to discontinue oral antibiotics.[9]

Spironolactone is already used off license for women with acne, is an inexpensive treatment choice and could play a role in reducing antibiotic use.[10] Literature searches did not, however, find any previously published economic evaluations on the cost-effectiveness of spironolactone in this group of patients, although there are two other ongoing studies of spironolactone in France and the USA, the former of which includes an economic evaluation.[11,12] In this paper we estimate the cost-effectiveness of spironolactone plus routine topical treatment compared to no active systemic treatment plus topical treatment for persistent acne in adult women.

PATIENTS AND METHODS

The Spironolactone for Adult Female Acne (SAFA) trial was a pragmatic, multicentre, participant-led, and clinician-blind, superiority, randomised trial with two parallel treatment groups: spironolactone compared to placebo in women aged 18 years and older with facial acne judged to warrant oral antibiotics. The economic evaluation was nested within this trial.

Participants were recruited in primary care, secondary care and through advertising (community and social media). Baseline assessment was conducted by a research nurse and/or dermatologist in secondary care clinics to ensure standard clinical assessments, as the Investigator's Global Assessment (IGA) for acne was an inclusion criterion and an important secondary outcome. Baseline appointments included a pregnancy test, blood test (to exclude renal impairment or raised serum potassium), participant photo to aid recall about changes in acne and contraceptive counselling. The first participant was recruited in June 2019 and the last in August 2021, whilst follow-up finished February 2022. The SAFA trial is described in more detail in the clinical paper.[13,14]

Participants were randomised 1:1 using online software to either 50 mg/day spironolactone or

For peer review only - http://bmjopen.bmj.com/site/about/guidelines.xhtml

matched placebo until week-6, increasing to 100 mg/day spironolactone or matched placebo until week-24, assuming treatment was tolerated. Participants were stratified by recruitment centre and baseline acne severity (IGA<3 vs IGA≥ 3). In both groups participants could continue using topical treatment. Between baseline and week-12 participants were asked not to take oral treatment for acne other than study medication, except for oral contraception taken for over 3 months previously. At week-12, participants in both groups could receive usual care, including oral treatments, such as oral antibiotics, hormonal treatment or isotretinoin. In both groups participants were followed up face-to-face (or by video call or telephone due to COVID-19) at week-6 and week-12 in secondary care, with primary outcome assessment at week-12, and longer-term follow-up by questionnaires at week-24.

Measuring costs

In keeping with an NHS perspective, all acne-related resource use data, including intervention, primary and secondary care visits, and prescription medication use, were collected. Personal Social Services (PSS) resource use was not collected, as patient and clinician contributors did not anticipate these being incurred by participants.

Resource use data was collected via case report forms and participant questionnaires, designed with the input of public contributors, at baseline (collecting the preceding 6 weeks), week-6, week-12 and week-24 for the intervention phase.

Resource uses were valued using UK unit costs (£ Sterling) for the most current price year available at the start of analysis (financial year 2021) and identified from published sources.

The intervention was costed as described in

Figure 1, which assumes that standard care, if adopted, will be delivered in primary care, including two GP visits (unless >45 years of age), baseline blood test and the cost of spironolactone (50 mg 6 weeks, 100 mg 18 weeks).[8,15–17] No intervention costs (placebo tablets, GP visits or blood tests) were included for the no active systemic treatment group as these would not occur if no intervention was being given (the comparator for this economic evaluation).

Acne-related resource use data related to visits to community-based healthcare professionals (HCP), visits to hospital out-patient and in-patient services (including accident and emergency) and prescribed medication costs were self-reported via participant questionnaires at all time-points, including baseline. Unit costs for each visit-type were combined with this data to estimate the total community-based HCP visit costs and the total hospital contact costs. Participants were also asked for details of prescribed acne-related medication including type, strength and quantity. Unit costs for all medication types[18] were used to estimate the prescription costs over the 24-week treatment period.

The mean (sd) cost per participant per intervention group was estimated for the 24-week treatment period, for each of the cost types described above and mean difference (95% CI) in NHS cost was estimated.

Measuring outcomes

The primary economic outcome measure was incremental cost per QALY over 24 weeks, as measured by the generic preference-based EQ-5D-5L questionnaire.[19] Responses were converted to utility scores using the EQ-5D-5L Crosswalk UK preference weights, as this was in line with

For peer review only - http://bmjopen.bmj.com/site/about/guidelines.xhtml

recommendations at the point analysis started, where utility ranges from -0.594 to 1.[20,21] Utility values were used to estimate QALYs over 24 weeks, using both linear interpolation and area under the curve analysis.[22]

A secondary economic outcome was the Acne-QoL symptom sub-scale score (five questions with seven responses to each)[23,24] at week-24, used as an estimate of effectiveness.

Economic analysis

The base-case cost-utility analysis (CUA) and secondary cost-effectiveness analysis (CEA) incorporated all randomised participants with complete cost and outcome data. Given the 24-week time-horizon, costs and benefits were not discounted.[21]

The base-case CUA estimated the incremental cost per QALY (incremental cost-effectiveness ratio, ICER) to enable comparison with the cost-utility of other interventions. The incremental cost (95% CI) and QALY change (95% CI) between groups was estimated unadjusted and adjusted for randomisation stratification variables (centre, baseline severity [IGA <3 versus ≥3]), and baseline variables (including Acne-QoL symptom subscale score, resource use costs, EQ-5D score and use of topical treatments (Y/N)). In line with NICE guidance,[21] we estimated whether the intervention was cost-effective by comparing the ICER with a cost-effectiveness threshold of £20,000 to £30,000 per QALY.

A CEA estimated the incremental cost per unit change on the Acne-QoL symptom sub-scale score. The incremental cost (95% CI) and Acne-QoL symptom sub-scale change (95% CI) between groups was estimated unadjusted and adjusted as described for the base-case CUA. The CUA and CEA were undertaken using a regression-based approach (seemingly unrelated regression equations).[25]

Published guidelines for the economic evaluation of health care interventions were followed as appropriate.[26,27]

To estimate the level of uncertainty associated with the decision regarding cost-effectiveness, Fieller's theorem was used to calculate [28] the probability of being cost-effective at the £20,000 and £30,000 willingness-to-pay threshold values.[21] Non-parametric bootstrapping was conducted to generate 10,000 estimates of incremental costs and benefits. From this Cost-Effectiveness Acceptability Curves (CEACs) were generated to show the probability that the intervention is estimated to be cost-effective at different willingness-to-pay values.

Several sensitivity analyses were agreed before analysis and undertaken to explore key uncertainties around important parameters in the economic evaluation. The impact of missing data on cost-effectiveness estimates was explored by undertaking multiple imputation, assuming that the data was missing at random and using chained equations to handle the missing cost and outcome data.[28] Secondly, the CUA was repeated assuming the NICE guidance[29] on acne was followed, meaning all women in the placebo group took oral antibiotics (lymecycline or doxycycline, 1 tablet daily for 24 weeks), in addition to topical treatment, as all had persistent acne. To cost this intervention the weighted mean cost per dose of doxycycline/lymecycline was used (Table 1) and two GP visits assumed. We assumed that the health-related quality of life remained unchanged from what was collected in the SAFA trial, even though incremental QALYs are likely to be lower with effective antibiotic use. A threshold analysis was performed to ascertain what level of incremental QALYs

For peer review only - http://bmjopen.bmj.com/site/about/guidelines.xhtml

would switch the intervention between cost-effective and cost-ineffective. The societal costs of over prescribing of oral antibiotics were not included. Further sensitivity analyses in which perspective and delivery of intervention are varied and a sub-group analysis based on age is presented in supplementary material. Further details and additional sensitivity analyses and sub-group analysis are presented in online supplementary material.

Stata MP version 17 was used to conduct the analyses. A health economic analysis plan was written and followed; a copy is available from the corresponding author.

Patient and public involvement (PPI)

Key questions relating to research design were explored with a virtual acne-specific patient panel and patient survey carried out via the UK Dermatology Clinical Trials Network (UKDCTN). Two public contributors (IS and KaT) with experience of acne were members of the Trial Management group as part of this role they helped identify relevant resources and outcomes and how this data should be collected. They also contributed to the interpretation and write-up if the health economics component.

RESULTS

Participant characteristics

The clinical trial results, including details on sample size and participant characteristics, are reported elsewhere.[14,30] Of the 410 women recruited to the trial, 201 were randomly assigned to spironolactone alongside routine topical treatment and 209 allocated to placebo alongside routine topical treatment at the start of the trial. At week-24 126 women in the spironolactone group and 109 women in the placebo group had complete cost and outcome data, and these formed the base-case unadjusted CUA. Mean age was 29.2 years, mean BMI was 26.1, topical acne treatments were used by 83% (340/410) and the majority (75% [306/410]) had acne for two or more years. There were no significant differences in characteristics between groups.[14]

Costs

The Unit costs used in the analysis are presented in Table 1. The levels of resource use in each group were very similar prior to randomisation (Table S1).

The majority of responding women in the spironolactone group (182/184, 99%) increased to two tablets of spironolactone at week-6. The 'standard of care' approach used in the base-case economic evaluation, gave rise to a mean total intervention resource use cost of £122.87 (SD £13.04) per participant in the spironolactone group (Table 2).

Using available case data, when intervention use was combined with other health resource use, the unadjusted mean incremental cost per participant was £126.35 (95% CI, £112.88 to £139.82) for women receiving spironolactone compared to women receiving placebo in the base-case (Table 2). Excluding intervention costs, the difference was not significant between groups. While patients were asked about in-patient visits, none were reported.

Outcomes

The mean (sd) QALYs in the spironolactone group were 0.417 (0.058) per participant compared to 0.404 (0.079) per participant in the placebo group, giving an incremental difference of 0.013 (95% CI -0.0024 to 0.0289) QALYs using unadjusted available case data (Table 2).

For peer review only - http://bmjopen.bmj.com/site/about/guidelines.xhtml

The mean (sd) change from baseline in Acne-QoL symptom subscale score at 24 weeks was 8.15 (6.12) in the spironolactone group compared to 4.46 (6.34) in the placebo group. Thus, the incremental difference in score was 3.68 (95% CI 2.26 to 5.11) in favour of the spironolactone group (Table 2).

Base-case Cost Utility Analysis

In the complete case analysis, the incremental cost for the spironolactone (n=118) compared to the no active intervention (n=101) was £125.36 (95% CI, £111.13 to £139.58) (unadjusted this was £125.53 [95% CI £112.15 to £138.91]) (Table 3). The adjusted incremental QALYs for the spironolactone group compared with the no active intervention was 0.0019 (95% -0.0096 to 0.0133) (unadjusted was 0.0036, 95% CI -0.0117 to 0.0189). The ICER was £67,191 (unadjusted £34,770) per QALY. At a willingness to pay of £30,000 per QALY there was a 35% (unadjusted 47%) chance of spironolactone being cost-effective in this population of women with persistent acne.

The cost-effectiveness acceptability curves (**Error! Reference source not found.**) of the adjusted and unadjusted base-case analysis, show that the probability of spironolactone being cost-effective only approaches 50% as the threshold value approaches £120,000 (adjusted), demonstrating a high degree of uncertainty associated with the decision under these conditions.

Secondary Cost Effectiveness analysis

The adjusted incremental difference in cost per point change on the Acne-QoL symptom subscale for the spironolactone (n=119) compared to no active intervention (n=102) was £38.21 (unadjusted £35.91) based on a complete case analysis (Table). How much a decision maker would be willing to pay for a point change on the Acne-Qol symptom subscale is unknown.

Sensitivity analyses

There were differential rates of attrition with greater missing data in the placebo group, compared to spironolactone group, by 24-weeks follow-up, for costs (39% vs. 24%, respectively) and EQ-5D-5L (33% vs. 20%, respectively). When using multiple imputation in a sensitivity analysis the ICER was less than in the complete case, adjusted analysis (£27,879 compared with £67,191 per QALY) with a 53% probability of spironolactone being cost effective at £30,000 per QALY (Table 3).

There were 10 participants who reported taking an oral antibiotic (lymecycline/oxytetracycline/ doxycycline) between baseline and week 24, 6 participants in the placebo group and 4 participants in the spironolactone group. In a sensitivity analysis assuming all women in the placebo group took an oral antibiotic for the 24-week treatment period,[29] an ICER of £9,169 per QALY was estimated in a complete case analysis with a 59% probability of spironolactone being cost-effective at a £30,000 per QALY threshold. With multiple imputation, the ICER was £2,683 per QALY with an 82% probability that spironolactone would be cost effective at a £30,000 threshold (Table 3). Threshold analysis using the complete case analysis data found that incremental QALYs would have to decrease to 0.00057 in order to switch the ICER from cost-effective to cost-ineffective at a £30,000 threshold.

The results of additional sensitivity analyses and a subgroup analysis based on age are reported in online supplementary material. In particular, a wider perspective is taken including participant and carer out of pocket and productivity costs (see Table S2) and a sensitivity analysis is taken costing the intervention resource use as delivered via secondary care per trial protocol (see Figure S1). A sub-group analysis is undertaken for women aged <25 years and ⩾25 years. See Table S3 for results.

For peer review only - http://bmjopen.bmj.com/site/about/guidelines.xhtml

DISCUSSION

Our economic evaluation provides a range of estimates for the cost effectiveness of spironolactone used alongside routine topical treatment. The base-case analysis, where the comparator is that used in the trial (no active systemic treatment plus routine topical treatment) and the delivery of the intervention is costed as via primary care, spironolactone was estimated to be cost ineffective in the unadjusted and adjusted complete case analyses. However, in the adjusted analysis using multiple imputation (MI) the ICER was estimated to be just under the £30,000 per QALY threshold. This divergence in conclusion between the complete case and MI analysis demonstrates the impact of missing data (attrition bias) and suggests more weight ought to be placed on the MI analysis.[31] In sensitivity analyses, where a more realistic comparator (standard of care based upon NICE guidelines) is used, spironolactone is found to be highly cost-effective. Threshold analysis around the level of incremental QALYs assumed in this analysis, demonstrates that the conclusion is likely to hold true even if the incremental QALYs gained are less than those observed in the trial.

The strength of this economic evaluation is that it can provide reliable estimates of cost effectiveness based on individual participant level data, collected at little marginal cost, alongside a randomised controlled trial. This is, however, also a limitation in that within trial health economic evaluations are constrained by the question, timeframe, and data collected, particularly in placebo-controlled trials. Placebos are not used in routine practice, but some evidence of placebo effects has been documented in acne.[32] This may result in underestimation of the QALY gain with spironolactone compared with not providing spironolactone, and hence underestimate its cost-effectiveness. The base-case set out to answer the question of whether spironolactone is cost-effective compared with no active systemic treatment (both groups could use routine topical medications) to align with the clinical question funded. In practice, this analysis may not be the most informative for clinicians who would be unlikely to send women away with no active treatment if they consulted with acne persisting beyond 6 months. To address this a sensitivity analysis assuming, for cost purposes, all women in the placebo group received an oral antibiotic for 24 weeks was performed. The limitations of this analysis were acknowledged above but despite these limitations the analysis serves to provide a lower range estimate for the cost effectiveness of spironolactone that better reflects accepted standard-of-care, based upon current NICE guidelines.[29]

In our study the conclusion reached about cost-effectiveness was sensitive to the estimates of QALYs generated from EQ-5D-5L, despite 46% in the intervention group and 43% in the control group reporting perfect health (EQ-5D-5L health state 11111) at baseline. For these participants, the EQ-5D-5L had no potential to measure improvements in health-related quality of life. At design stage, there was discussion about the possible use of other instruments, however, the limited published evidence supported the use of the EQ-5D for acne.[33,34] Like Klassen et al [34] we find that women with persistent acne report most problems on the pain/discomfort and anxiety/depression dimensions of the EQ-5D.

Further research using the EQ-5D data generated in this study alongside that elicited in other studies of acne would help inform future studies about the validity and responsiveness of this instrument for acne.

Whether spironolactone is considered a cost-effective treatment for persistent acne in women, depends upon the assumptions and methods utilised. On balance, when dealing with missing data or

For peer review only - http://bmjopen.bmj.com/site/about/guidelines.xhtml

considering a more realistic comparator (providing antibiotic treatment), the likely conclusion is that spironolactone is cost-effective. This is particularly credible, given that this study plausibly underestimates the economic benefit of spironolactone as, given the timeframe, it was not feasible to capture the potential benefits of reduced prescribing of antibiotics on antimicrobial resistance.

For peer review only

For peer review only - http://bmjopen.bmj.com/site/about/guidelines.xhtml

ACKNOWLEDGEMENTS

We would like to thank all PPI contributors, participants, research and clinical staff, the NIHR Clinical Research Network, and the members of the Trial Steering Committee and Data Monitoring Committee for their support.

MS, AL, BS, THS, MJR, NF, KST, PL, JN, GG and IM conceived the study idea and initial study design in response to a NIHR HTA call, with later input from KLT, IS, ZE, SR, ML, NP and SP. Specific advice was given by BS on trial design and medical statistics; and THS on health economic evaluation. Economic analyses were conducted by SP and THS. All authors contributed to the drafting of this paper, led by SP and THS, and approved the final manuscript.

The study was developed with support from the UK Dermatology Clinical Trials Network (UK DCTN). The UK DCTN is grateful to the British Association of Dermatologists and the University of Nottingham for financial support of the Network.

The University of Southampton was the research sponsor for this trial.

DATA AVAILABILITY

Consent was not obtained from participants for data sharing but authors will consider reasonable requests to make relevant anonymised participant level data available.

ETHICS STATEMENT

This study was conducted in compliance with the ethical principles of the Declaration of Helsinki and in compliance with all International Council for Harmonisation Good Clinical Practice guidelines. The study protocol was reviewed and approved by the Institutional Review board and/or Independent Ethics Committee at each participating centre. All participants provided written informed consent.

Ethical approval for the trial was given by Wales Research Ethics Committee (REC) 3 in January 2019 (18/WA/0420).

SUPPORTING INFORMATION

Additional supporting information may be found in the online version of this article at the publisher's website:

Appendix S1 Supplementary material: further sensitivity and sub-group analyses

Figure S1 Intervention resource use as delivered via secondary care, per trial protocol

Table S1 Estimates of mean baseline resource use by treatment group (available case data)

Table S2 Estimates of mean change in cost (UK£ 2021/22) including wider costs, by treatment group

Table S3 Cost utility analyses and cost-effectiveness analyses results, for additional sensitivity analyses and sub-group analysis

For peer review only

REFERENCES

1. Purdy S, de Berker D. Acne. *BMJ* 2006; 333: 949–953.

2. Schofield J, Grindlay D, Williams H. *Skin conditions in the UK: a Health Care Needs Assessment*. University of Nottingham, 2009.

3. Cunliffe WJ. Acne and unemployment. *Br J Dermatol* 1986; 115: 386–386.

4. Jowett S, Ryan T. Skin disease and handicap: an analysis of the impact of skin conditions. *Soc Sci Med* 1985; 20: 425–9.

5. NICE. *Acne vulgaris: management NICE guideline*, www.nice.org.uk/guidance/ng198 (2021, accessed 16 November 2022).

6. le Cleach L, Lebrun-Vignes B, Bachelot A, et al. Guidelines for the management of acne: recommendations from a French multidisciplinary group. *Br J Dermatol* 2017; 177: 908–913.

7. Thiboutot DM, Dréno B, Abanmi A, et al. Practical management of acne for clinicians: An international consensus from the Global Alliance to Improve Outcomes in Acne. *J Am Acad Dermatol* 2018; 78: S1-S23.e1.

8. Zaenglein AL, Pathy AL, Schlosser BJ, et al. Guidelines of care for the management of acne vulgaris. *J Am Acad Dermatol* 2016; 74: 945-973.e33.

9. Platt D, Muller I, Sufraz A, et al. GPs' perspectives on acne management in primary care: a qualitative interview study. *Br J Gen Pract* 2020; 71: E78–E84.

10. Layton AM, Eady EA, Whitehouse H, et al. Oral Spironolactone for Acne Vulgaris in Adult Females: A Hybrid Systematic Review. *Am J Clin Dermatol* 2017; 18: 169–191.

11. Barbieri Lab. Spironolactone versus doxycycline for acne: a comparative non-inferiority evaluation (SD-ACNE) research study, https://barbierilab.bwh.harvard.edu/clinical-trial-opportunities/ (accessed 17 November 2022).

12. Poinas A, Lemoigne M, le Naour S, et al. FASCE, the benefit of spironolactone for treating acne in women: study protocol for a randomized double-blind trial. *Trials* 2020; 21: 571.

13. Renz S, Chinnery F, Stuart B, et al. Spironolactone for adult female acne (SAFA): protocol for a double-blind, placebo-controlled, phase III randomised study of spironolactone as systemic therapy for acne in adult women. *BMJ Open* 2021; 11: e053876.

14. Santer M, Lawrence M, Eminton Z, et al. Spironolactone for Adult Female Acne (SAFA): multicentre double-blind randomised trial. *BMJ* 2023; BMJ-2022-074349: (Submitted).

15. Thiede RM, Rastogi S, Nardone B, et al. Hyperkalemia in women with acne exposed to oral spironolactone: A retrospective study from the RADAR (Research on Adverse Drug Events and Reports) program. *Int J Womens Dermatol* 2019; 5: 155–157.

For peer review only - http://bmjopen.bmj.com/site/about/guidelines.xhtml

16. Plovanich M, YuWeng Q, Mostaghimi A. Low Usefulness of Potassium Monitoring Among Healthy Young Women Taking Spironolactone for Acne. *JAMA Dermatol* 2015; 151: 941–944.

17. Wang Y, Lipner SR. Retrospective analysis of adverse events with spironolactone in females reported to the United States Food and Drug Administration. *Int J Womens Dermatol* 2020; 6: 272.

18. NHS Business Service Authority. *Prescription Cost Analysis - England 2020/21*, https://www.nhsbsa.nhs.uk/statistical-collections/prescription-cost-analysis-england/prescription-cost-analysis-england-202021 (2021, accessed 30 November 2022).

19. Herdman M, Gudex C, Lloyd A, et al. Development and preliminary testing of the new five-level version of EQ-5D (EQ-5D-5L). *Qual Life Res* 2011; 20: 1727–36.

20. van Hout B, Janssen MF, Feng YS, et al. Interim scoring for the EQ-5D-5L: Mapping the EQ-5D-5L to EQ-5D-3L value sets. *Value in Health* 2012; 15: 708–715.

21. NICE. Guide to the methods of technology appraisal 2013. *National Institute for Health and Care Excellence* 2013; 1–93.

22. Manca A, Hawkins N, Sculpher MJ. Estimating mean QALYs in trial-based cost-effectiveness analysis: The importance of controlling for baseline utility. *Health Econ* 2005; 14: 487–496.

23. Fehnel SE, McLeod LD, Brandman J, et al. Responsiveness of the Acne-Specific Quality of Life Questionnaire (Acne-QoL) to treatment for acne vulgaris in placebo-controlled clinical trials. *Qual Life Res* 2002; 11: 809–816.

24. Martin AR, Lookingbill DP, Botek A, et al. Health-related quality of life among patients with facial acne -- assessment of a new acne-specific questionnaire. *Clin Exp Dermatol* 2001; 26: 380–385.

25. Willan AR, Briggs AH, Hoch JS. Regression methods for covariate adjustment and subgroup analysis for non-censored cost-effectiveness data. *Health Econ* 2004; 13: 461–475.

26. Drummond M, Sculpher M, Claxton K, et al. *Methods for the economic evaluation of health care programmes*. 4th ed. Oxford, UK: Oxford University Press, 2015.

27. Ramsey SD, Willke RJ, Glick H, et al. Cost-effectiveness analysis alongside clinical trials II - An ISPOR good research practices task force report. *Value in Health* 2015; 18: 161–172.

28. Faria R, Gomes M, Epstein D, et al. A Guide to Handling Missing Data in Cost-Effectiveness Analysis Conducted Within Randomised Controlled Trials. *Pharmacoeconomics* 2014; 32: 1157–1170.

29. Xu J, Mavranezouli I, Kuznetsov L, et al. Management of acne vulgaris: summary of NICE guidance. *BMJ* 2021; 374: n1800.

30. Santer M, Layton A, Muller I, et al. Spironolactone for Adult Female Acne (SAFA): pragmatic multicentre double-blind randomised superiority trial to investigate the clinical and cost-effectiveness of spironolactone for moderate or severe persistent acne in women - NIHR

For peer review only - http://bmjopen.bmj.com/site/about/guidelines.xhtml

Funding and Awards, https://www.fundingawards.nihr.ac.uk/award/16/13/02 (accessed 14 December 2022).

31. Leurent B, Gomes M, Faria R, et al. Sensitivity Analysis for Not-at-Random Missing Data in Trial-Based Cost-Effectiveness Analysis: A Tutorial. *Pharmacoeconomics* 2018; 36: 889–901.

32. Mavranezouli I, Daly CH, Welton NJ, et al. A systematic review and network meta-analysis of topical pharmacological, oral pharmacological, physical and combined treatments for acne vulgaris. *Br J Dermatol* 2022; 187: 639–649.

33. Yang Y, Brazier J, Longworth L. EQ-5D in skin conditions: an assessment of validity and responsiveness. *European Journal of Health Economics* 2015; 16: 927–939.

34. Klassen AF, Newton JN, Mallon E. Measuring quality of life in people referred for specialist care of acne: Comparing generic and disease-specific measures. *J Am Acad Dermatol* 2000; 43: 229–233.

35. Jones K, Burns A. Unit Costs of Health and Social Care 2021. *PSSRU*, https://www.pssru.ac.uk/project-pages/unit-costs/unit-costs-of-health-and-social-care-2021/ (2021, accessed 5 December 2022).

36. NHS England. *2019/2020 National Cost Collection Data Publication*. London, https://www.england.nhs.uk/publication/2019-20-national-cost-collection-data-publication/ (2021, accessed 5 December 2022).

37. Curtis L, Burns A. *Unit costs of health and social care 2015*. Personal Social Services Research Unit, University of Kent, Canterbury, 2015.

38. UKHCA. *UKHCA Commisioning Survey 2012: Care is not a Commodity*. 2012.

39. Brown R. *PSNC Pharmacy Advice Audit 2021*. 2021.

40. Office for National Statistics. Annual Survey of Hours and Earnings time series of selected estimates, https://www.ons.gov.uk/employmentandlabourmarket/peopleinwork/earningsandworkinghours/datasets/ashe1997to2015selectedestimates (2021, accessed 5 December 2022).

For peer review only

For peer review only - http://bmjopen.bmj.com/site/about/guidelines.xhtml

FIGURES

Figure 1 | Intervention resource use as per standard of care (base-case)

Figure 2 | Cost Effectiveness Acceptability Curve (CEAC), complete case analysis, adjusted and unadjusted QALYs

For peer review only

For peer review only - http://bmjopen.bmj.com/site/about/guidelines.xhtml

TABLES

Table 1 | Unit costs (UK£ sterling, 2020/21 financial year)

Cost Item	Unit Cost (£)	Unit	Source, assumptions
**Intervention**			
Spironolactone with dose escalation	£49.37	Total	Prescription Cost Analysis 2021.[18]
GP visit related to intervention	£33.00	Total	PSSRU Unit costs 2021.[35]
Blood test for renal function (eGFR) and potassium level (K serum)	£5.22	Total	National Cost Collection 2020.[36] *
**Medication costs**	Mean cost per quantity		
Topical preparations for acne	£0.96	gram/ml	Prescription Cost Analysis 2021.[18] Mean across all medications in each medication type. Weighted averages taken where listed >1x. Weighted average for estimating oral antibiotic control for SA (see table 3). Assumes 1x100 mg (doxycycline)/408 mg (lymecycline) per day for 24 weeks.
Other topical preparation	£0.03	gram/ml	
Oral contraceptives	£0.08	tablet	
Oral antibiotics	£0.22	capsule/tablet	
Anti-depressants	£0.20	capsule/tablet	
Analgesics	£0.04	capsule/tablet	
PCOS/diabetes medication	£0.03	tablet	
Other medications	£0.40	various	
Doxycycline/lymecycline weighted average	£0.25	Capsule	
**Community-based HCP contacts**			
GP visit unrelated to intervention	£33.00	Visit	PSSRU Unit costs 2021.[35]
Practice Nurse	£14.13	Visit	PSSRU Unit costs 2021 & 2015.[35,37]
NHS Walk-in centre	£71.99	Visit	National Cost Collection 2020.[36] Weighted average of all community health services.*
Community dermatology service	£121.01	Visit	National Cost Collection 2020.[36] *
Healthcare assistant	£14.44	Visit	PSSRU Unit Costs 2021[35] & UKHCA Commissioning Survey 2012.[38]
Pharmacist	£6.99	Visit	PSSRU Unit costs 2021 & 2015[35,37] & PSNC Pharmacy Advice Audit 2021.[39]
Physiotherapist	£66.82	Visit	National Cost Collection 2020.[36] *
Dietician	£82.46	Visit	National Cost Collection 2020.[36] *
Other (community)	£33.00	Visit	PSSRU Unit costs 2021. Used most common visit: GP visit.[35]
**Hospital out-patient contacts**			
Dermatologist	£128.25	Visit	National Cost Collection 2020.[36] *

For peer review only - http://bmjopen.bmj.com/site/about/guidelines.xhtml

Dermatology Nurse	£100.71	Visit	National Cost Collection 2020.[36] *
Ear, nose and throat (ENT)	£116.11	Visit	National Cost Collection 2020.[36] *
Interventional radiology	£137.64	Visit	National Cost Collection 2020.[36] *
Trauma and orthopaedics	£125.67	Visit	National Cost Collection 2020.[36] *
Respiratory medicine	£161.07	Visit	National Cost Collection 2020.[36] *
Other (out-patient)	£137.10	Visit	National Cost Collection 2020.[36] *
**Hospital admission**			
Accident and emergency	£182.28	Visit	National Cost Collection 2020. Index/Accident & Emergency.[36] *
**Wider costs**			
Personal out-of-pocket expenses	Various	Per item	Participant reported.
Lost work time	£18.01	Hour	ONS 2021.[40] Mean hourly earnings, excluding overtime (£).

SA, sensitivity analysis; ONS, Office for National Statistics; PSSRU, Personal Social Services Research Unit.

* Inflated to 2021 prices as per NHSCII Pay & Prices.[35]

For peer review only

Table 2 | Estimates of mean change in resource use and cost (UK£ 2021/22) and mean utility and QALY gain by treatment group

Resource	Spironolactone (N=201)		Placebo (N=209)		Mean difference
	Mean (n)	SD	Mean (n)	SD	(95% CI)
Resource use over 24-week period:					
Spironolactone (number)	294 (201)	0	0 (209)	0	-
GP visits related to intervention (no. of visits)*	2.06 (201)	0.34	0 (209)	0	-
Blood tests – renal function (eGFR) and potassium level (number)	1.06 (201)	0.34	0 (209)	0	-
Total community-based HCP visits (number)	0.15 (150)	0.51	0.10 (124)	0.43	0.05 (-0.064 to 0.164)
Total hospital contacts (number)	0.06 (132)	0.30	0.05 (115)	0.26	0.01 (-0.062 to 0.079)
All prescription medications (number)	11.42 (147)	29.65	23.36 (124)	96.80	-11.94 (-28.507 to 4.625)
Costs over 24-week period:	UK£2021/22				
All intervention costs	122.87 (201)	13.04	0 (209)	0	122.87 (121.09 to 124.64)
All community-based HCP costs	6.28 (150)	24.83	3.75 (124)	16.46	2.53 (-2.60 to 7.66)
All hospital contact costs	7.28 (132)	36.42	5.73 (115)	28.09	1.55 (-6.70 to 9.79)
All prescription medication costs	4.37 (147)	11.77	5.91 (124)	18.93	-1.54 (-5.25 to 2.17)
**Total costs**	**141.99 (128)**	**57.90**	**15.64 (110)**	**45.62**	**126.35 (112.88 to 139.82)**
**Total costs excluding intervention**	**19.61 (128)**	**56.65**	**15.64 (110)**	**45.62**	**3.98 (-9.30 to 17.26)**
EQ-5D score (CUA)					
Baseline	0.887 (200)	0.148	0.860 (209)	0.200	0.027 (-0.008 to 0.061)
6 weeks	0.894 (176)	0.135	0.863 (179)	0.168	0.031 (-0.001 to 0.063)
12 weeks	0.904 (174)	0.138	0.877 (166)	0.177	0.027 (-0.007 to 0.061)
24 weeks	0.909 (163)	0.153	0.890 (136)	0.180	0.019 (-0.019 to 0.057)
**Total QALY score**	**0.417 (162)**	**0.058**	**0.404 (136)**	**0.079**	**0.013 (-0.002 to 0.029)**
Acne-QoL symptom sub-scale score (CEA)					
Baseline	13.22 (201)	4.94	12.87 (209)	4.55	0.35 (-0.57 to 1.27)
6 weeks	16.97 (176)	5.72	15.65 (179)	5.69	1.32 (0.13 to 2.51)
12 weeks	19.21 (176)	6.12	17.76 (166)	5.58	1.45 (0.20 to 2.69)
24 weeks	21.22 (163)	5.86	17.39 (136)	5.80	3.83 (2.49 to 5.16)
**Change at 24-weeks from baseline**	**8.15 (163)**	**6.12**	**4.46 (136)**	**6.34**	**3.68 (2.26 to 5.11)**

*Assumes that if spironolactone is found effective it would be prescribed in primary care.

For peer review only

For peer review only - http://bmjopen.bmj.com/site/about/guidelines.xhtml

Table 3 | Cost utility analyses and cost-effectiveness analyses results, including sensitivity analyses and sub-group analysis

CUA Analysis (N s, N p)	Incremental cost (95% CI)	Incremental QALYs (95% CI)	ICER	CEAC at £20,000 (£30,000) threshold*
Base-case, CCA, adjusted (118,101)	125.36 (111.13 to 139.58)	0.0019 (-0.0096 to 0.0133)	£67,191	23% (35%)
Base-case, CCA, unadjusted (126,109)	125.53 (112.15 to 138.91)	0.0036 (-0.0117 to 0.0189)	£34,770	37% (47%)
SA, Multiple imputation, adjusted (201,209)	119.78 (107.99 to 131.57)	0.0043 (-0.0041 to 0.0127)	£27,879	35% (53%)
SA, oral antibiotic control, CCA, adjusted (118,101)	17.11 (2.88 to 31.33)	0.0019 (-0.096 to 0.0133)	£9,169	57% (59%)
SA, oral antibiotic control, MI, adjusted (201, 209)	11.53 (-0.26 to 23.32)	0.0043 (-0.0041 to 0.0127)	£2,683	81% (82%)
**CEA Analysis (N s, N p)**	**Incremental cost (95% CI)**	**Incremental Acne-QoL symptom (95% CI)**	**Incremental cost per unit change**	-
Secondary analysis, CCA, adjusted: (119,102)	126.57 (112.35 to 140.78)	3.31 (1.90 to 4.72)	£38.21	-
Secondary analysis, CCA, unadjusted (127,110)	126.52 (113.00 to 140.04)	3.52 (1.94 to 5.11)	£35.91	-

95% CI=95% confidence interval; ICER =incremental cost-effectiveness ratio; N s / N p =Number randomised to spironolactone / Placebo who were included in the analysis; CCA = complete case analysis; SA refers to the different sensitivity analyses described in the Methods; QALY=Quality Adjusted Life Years; *probability of being cost-effective at a the threshold (λ) of £20,000 and £30,000 per QALY. Adjusted analyses, adjusted for stratification variables (centre, baseline severity [IGA<3 vs. ≥3]) and baseline variables (Acne QoL symptom subscale score, use of topical treatments, utility score based on EQ-5D, total costs)

For peer review only - http://bmjopen.bmj.com/site/about/guidelines.xhtml

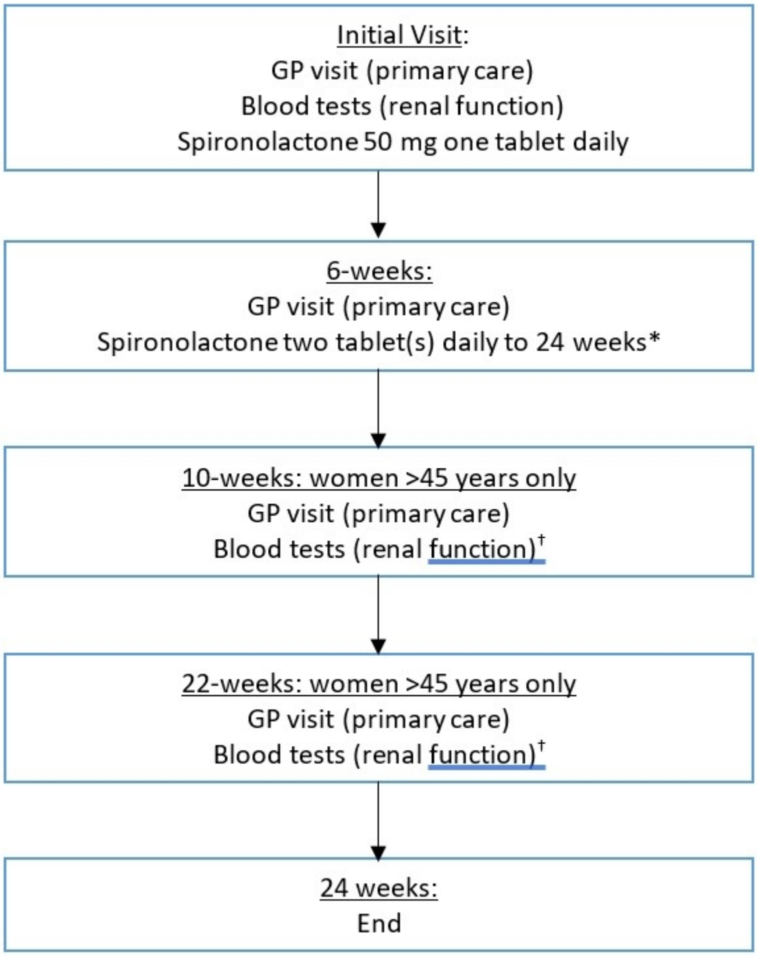

*Assumes all patients escalated to two 50 mg tablets spironolactone at 6-week visit. Based on the data from the trial, this was the case for 182/184 (99%) in the spironolactone group at 6 weeks (question response rate 184/201 in spironolactone group).

†As those with relevant comorbidities or on treatments with increased risk were not included in the trial, it is not possible to estimate the proportion of such patients that might receive spironolactone and need blood test monitoring.

Figure 1 Intervention resource use as per standard of care (base-case)

200x241mm (96 x 96 DPI)

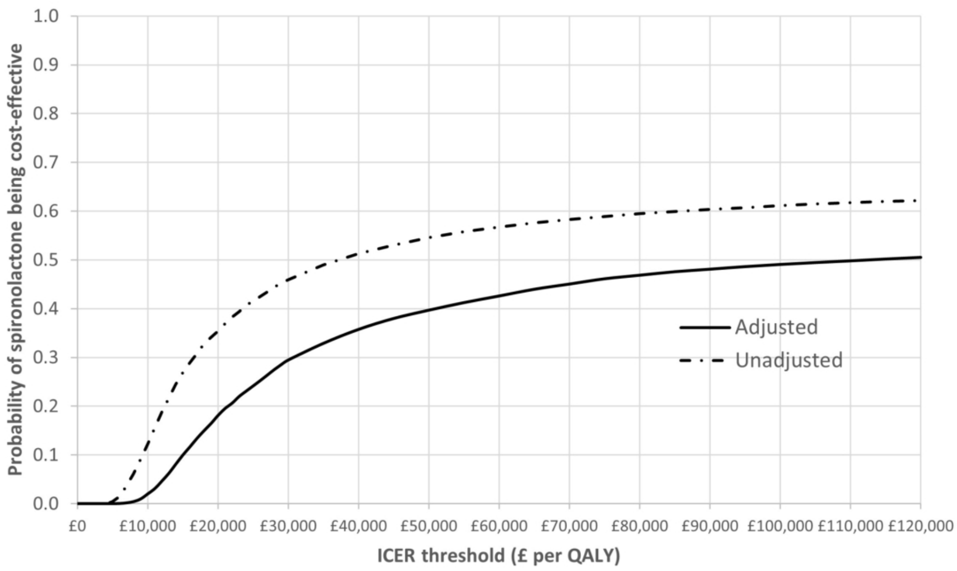

Figure 2 Cost Effectiveness Acceptability Curve (CEAC), complete case analysis, adjusted and unadjusted QALYs

182x116mm (300 x 300 DPI)

SUPPORTING INFORMATION

APPENDIX S1: FURTHER SENSITIVITY AND SUBGROUP ANALYSES

The following information is presented in addition to the main paper, "Cost-effectiveness of Spironolactone for Adult Female Acne (SAFA): Economic evaluation alongside a randomised controlled trial", published in XXXX. In addition to the sensitivity analyses presented in the main paper, a further two sensitivity analyses and a sub-group analysis were agreed before analysis and conducted to explore key uncertainties around the parameters of the economic evaluation. The details of these are outlined below.

Baseline Resource use

Table S1 presents the levels of resource use, at baseline, prior to randomisation (Table S1).

Sensitivity analysis: wider resource use

In addition to the resource use data outlined in the main paper and to enable further sensitivity analysis exploring a wider perspective than that limited to the NHS, wider resource use related to acne, were collected via the participant questionnaires at all time points. These included out-of-pocket expenses (including, complementary therapist visits, cosmetic skin care products, non-NHS-prescribed medication, parking and travel costs for healthcare appointments and other) and productivity losses (including lost patient and carer productivity). These were valued using participant self-reported values and unit costs identified from published sources and reported in Table 1.

The mean (sd) cost per participant per intervention group was estimated for the 24-week treatment period for personal out-of-pocket expenses and productivity costs and summed along with NHS costs to estimate the mean difference (95% CI) in total costs (wider perspective) (Table S2). The cost utility analysis was repeated as described in the main paper with this exception.

Taking a wider cost perspective including participants' and family/friend's out of pocket costs, productivity costs and non-official services costs, found spironolactone to be dominated by placebo in the complete case analysis, that is both more costly and less effective but this is likely to reflect the amount of missing data. With multiple imputation, the ICER was estimated as £30,249 per QALY, with a 50% probability of spironolactone being cost-effective at a £30,000 per QALY (Table S3). The majority of women (97%) reported no impact on their employment as a result of their acne and thus it is mainly out-of-pocket expenses driving change from the base-case.

Sensitivity analysis: costing the intervention as per the SAFA trial protocol

The impact of costing the intervention as per the SAFA trial protocol (i.e. intervention was accessed via secondary care, excluding any research related costs) was explored in a 'secondary care delivery' sensitivity analysis. The cost utility analysis was repeated but with the intervention costed as described in Figure S1, while the placebo group was costed as in the base-case analysis, i.e. assumed no intervention costs.

Costing the intervention as it was delivered in the trial, via secondary care (see **Error! Reference source not found.**) instead of primary care (the expected standard of care if delivered in practice), the mean cost of the spironolactone intervention was £264.94 (SD £36.14). The ICER was estimated

For peer review only - http://bmjopen.bmj.com/site/about/guidelines.xhtml

as £141,955 per QALY, with a 12% probability of spironolactone being cost-effective at a £30,000 threshold (Table S3).

Subgroup analysis by age

A single sub-group analysis was undertaken for age (categorised as below 25 years and 25 years and over) as the clinical analysis found age significantly interacts with the outcome.[1]

The ICER was £263,871 per QALY for women under 25 years compared to £19,994 for women over 25 years of age (see Table S3). This result suggests that spironolactone is likely to be cost effective for women aged over 25 years. Whilst this finding is in line with the clinical findings, it ought to be interpreted with caution given the small sample sizes necessitated by splitting the dataset into subgroups.

Interpretation of sensitivity and subgroup analyses

The results of these sensitivity analyses reinforce the conclusion given in the main paper, which stated that whether 'spironolactone is considered a cost-effective treatment for persistent acne in women, depends upon the assumptions and methods utilised'. As would be expected, when delivery of the intervention was assumed to take place in secondary care (as delivered in the trial protocol) spironolactone did not appear cost-effective. This is not, however, a realistic reflection of what is likely to occur in practice. When a wider perspective was taken (instead of an NHS perspective) spironolactone was again estimated not to be cost-effective. However, there was a large proportion of missing data and when this was considered the probability of the intervention being cost-effective increased, but the ICER was not quite below the £30,000 willingness to pay threshold. With regards to the sub-group analysis by age, further research to understand if and why there may be a difference in (cost) effectiveness of spironolactone by age is warranted before drawing any conclusions.

For peer review only

SUPPLEMENTARY FIGURES

Supplementary Figure S1 | Intervention resource use as delivered via secondary care per trial protocol

For peer review only

SUPPLEMENTARY TABLES

Supplementary Table S1 | Estimates of mean baseline resource use by treatment group (available case data)

Resource	Spironolactone (N=201)		Placebo (N=209)		Mean difference
	Mean (n)	Std dev	Mean (n)	Std dev	(95% CI)
Total community-based HCP visits	0.27 (200)	0.616	0.225 (209)	0.590	0.045 (-0.072 to 0.162)
Total hospital contacts	0.119 (193)	0.446	0.095 (200)	0.396	0.024 (-0.059 to 0.108)
All medications – quantity (number)	11.711 (201)	46.065	7.903 (206)	21.570	3.809 (-3.174 to 10.791)
Total out-of-pocket items	2.027 (188)	2.735	1.939 (196)	2.438	0.088 (-0.432 to 0.607)
Lost patient work time (number reporting)	0.020 (197)	0.141	0.034 (205)	0.182	-0.014 (-0.046 to 0.018)
Lost carer work time (number reporting)	0.015 (194)	0.124	0.030 (203)	0.170	-0.014 (-0.044 to 0.015)

For peer review only

Supplementary Table S2 | Estimates of mean (sd) cost and mean (95% CI) difference in cost (UK£ 2021/22) including wider costs, by treatment group

Resource	Spironolactone (N=201)		Placebo (N=209)		Mean difference
	**Mean (n)**	**SD**	**Mean (n)**	**SD**	**(95% CI)**
Resource use costs over 24-week period:					
All intervention costs	122.87 (201)	13.04	0 (209)	0	122.87 (121.09 to 124.64)
All community-based HCP costs	6.28 (150)	24.83	3.75 (124)	16.46	2.53 (-2.60 to 7.66)
All hospital contact costs	7.28 (132)	36.42	5.73 (115)	28.09	1.55 (-6.70 to 9.79)
All prescription medication costs	4.37 (147)	11.77	5.91 (124)	18.93	-1.54 (-5.25 to 2.17)
**Total costs (NHS perspective)**	**141.99 (128)**	**57.90**	**15.64 (110)**	**45.62**	**126.35 (112.88 to 139.82)**
All personal out-of-pocket costs	69.41 (139)	113.05	82.57 (120)	148.60	-13.15 (-45.23 to 18.92)
Lost patient and carer productivity	27.87 (177)	354.76	15.95 (179)	183.54	11.93 (-46.86 to 70.71)
**Total costs (wider perspective)**	**252.67 (113)**	**490.19**	**93.53 (100)**	**144.02**	**159.14 (58.86 to 259.41)**

For peer review only

Supplementary Table S3 | Cost utility analyses and cost-effectiveness analyses results, for additional sensitivity analyses and sub-group analysis

CUA Analysis (N s, N p)	Incremental cost (95% CI)	Incremental QALYs (95% CI)	ICER	CEAC at £20,000 (£30,000) threshold*
SA: Secondary care delivery, CCA, adjusted (118,101)	265.67 (250.52 to 280.82)	0.0019 (-0.0096 to 0.0133)	£141,955	3% (12%)
SA: Wider perspective, CCA, adjusted (97,85)	102.07 (64.21 to 139.92)	-0.0027 (-0.0139 to 0.0085)	Dominated	9% (15%)
SA: Wider perspective, MI, adjusted (201,209)	133.25 (72.52 to 193.93)	0.0044 (-0.0041 to 0.0129)	£30,249	31% (50%)
Sub-group analysis: <25 years, CCA, adjusted: (28,29)	108.23 (89.09 to 127.37)	0.0004 (-0.0141 to 0.0150)	£263,871	25% (33%)
Sub-group analysis: ≥25 years, CCA, adjusted: (90,72)	133.06 (114.97 to 151.16)	0.0067 (-0.0079 to 0.0213)	£19,994	50% (62%)

95% CI=95% confidence interval; ICER =incremental cost-effectiveness ratio; N s / N p =Number randomised to spironolactone / Placebo who were included in the analysis; CCA = complete case analysis; SA refers to the different sensitivity analyses described in the Methods; QALY=Quality Adjusted Life Years; *probability of being cost-effective at a the threshold (λ) of £20,000 and £30,000 per QALY. Adjusted analyses, adjusted for stratification variables (centre, baseline severity [IGA<3 vs. ≥3]) and baseline variables (Acne QoL symptom subscale score, use of topical treatments, utility score based on EQ-5D, total costs)

For peer review only

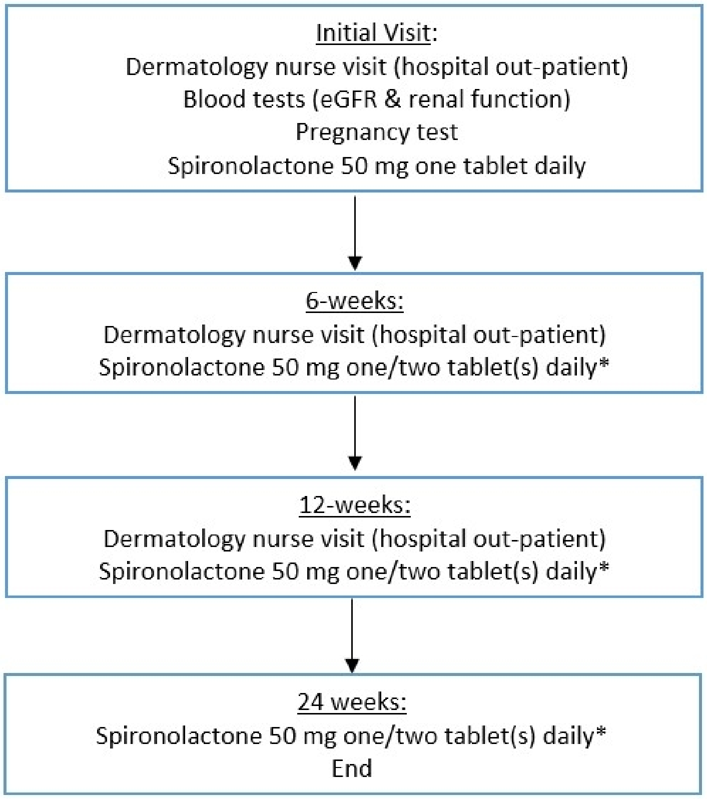

*Number of tablets taken to be informed by results of study

Figure S1 | Intervention resource use as delivered via secondary care per trial protocol (SA2)

186x187mm (96 x 96 DPI)

BMJ Open

Cost-effectiveness of Spironolactone for Adult Female Acne (SAFA): Economic evaluation alongside a randomised controlled trial

Journal:	*BMJ Open*
Manuscript ID	bmjopen-2023-073245.R1
Article Type:	Original research
Date Submitted by the Author:	19-May-2023
Complete List of Authors:	Pyne, Sarah; University of East Anglia, Health Economics Group, Norwich Medical School<br>Sach, Tracey; University of East Anglia, Health Economics Group, Norwich Medical School; University of Southampton, School of Primary Care, Population Sciences and Medical Education<br>Lawrence, Megan; University of Southampton<br>Renz, Susanne; University Hospital Southampton NHS Foundation Trust<br>Eminton, Zina; University Hospital Southampton NHS Foundation Trust<br>Stuart, Beth; Queen Mary University of London, Centre for Evaluation and Methods Wolfson Institute of Population Health<br>Thomas, Kim; University of Nottingham, Dermatology<br>Francis, Nick; University of Southampton, Primary Care Research Centre<br>Soulsby, Irene; University of Southampton Faculty of Medicine, Primary Care and Population Sciences<br>Thomas, Karen ; University of Southampton, Public contributor, Primary Care Research Centre<br>Permyakova, Natalia; University of Southampton; University Hospital Southampton NHS Foundation Trust<br>Ridd, Matthew; University of Bristol, Population Health Sciences<br>Little, Paul; University of Southampton<br>Muller, Ingrid; University of Southampton<br>Nuttall, Jacqui; University of Southampton<br>Griffiths, Gareth; University of Southampton<br>Layton, Alison M; University of York, Skin Research Centre, Hull York Medical School<br>Santer, Miriam; University of Southampton, Primary Medical Care
**Primary Subject Heading**:	Health economics
Secondary Subject Heading:	Dermatology
Keywords:	Clinical Trial, Dermatology < INTERNAL MEDICINE, Health economics < HEALTH SERVICES ADMINISTRATION & MANAGEMENT, HEALTH ECONOMICS, Acne < DERMATOLOGY, Adult dermatology < DERMATOLOGY

For peer review only - http://bmjopen.bmj.com/site/about/guidelines.xhtml

I, the Submitting Author has the right to grant and does grant on behalf of all authors of the Work (as defined in the below author licence), an exclusive licence and/or a non-exclusive licence for contributions from authors who are: i) UK Crown employees; ii) where BMJ has agreed a CC-BY licence shall apply, and/or iii) in accordance with the terms applicable for US Federal Government officers or employees acting as part of their official duties; on a worldwide, perpetual, irrevocable, royalty-free basis to BMJ Publishing Group Ltd ("BMJ") its licensees and where the relevant Journal is co-owned by BMJ to the co-owners of the Journal, to publish the Work in this journal and any other BMJ products and to exploit all rights, as set out in our licence.

The Submitting Author accepts and understands that any supply made under these terms is made by BMJ to the Submitting Author unless you are acting as an employee on behalf of your employer or a postgraduate student of an affiliated institution which is paying any applicable article publishing charge ("APC") for Open Access articles. Where the Submitting Author wishes to make the Work available on an Open Access basis (and intends to pay the relevant APC), the terms of reuse of such Open Access shall be governed by a Creative Commons licence – details of these licences and which Creative Commons licence will apply to this Work are set out in our licence referred to above.

Other than as permitted in any relevant BMJ Author's Self Archiving Policies, I confirm this Work has not been accepted for publication elsewhere, is not being considered for publication elsewhere and does not duplicate material already published. I confirm all authors consent to publication of this Work and authorise the granting of this licence.

For peer review only

TITLE:

Cost-effectiveness of Spironolactone for Adult Female Acne (SAFA): Economic evaluation alongside a randomised controlled trial

manuscript word count: 4,085 Words
Table count: 3
Figure count: 2

AUTHORS:

Sarah Pyne,[1] Tracey H Sach*,[1, 2] Megan Lawrence,[3] Susanne Renz,[3] Zina Eminton,[3] Beth Stuart,[2, 4] Kim S Thomas,[5] Nick Francis,[2] Irene Soulsby,[6] Karen Thomas,[6] Natalia Permyakova,[3] Matthew J Ridd,[7] Paul Little,[4] Ingrid Muller,[2] Jacqueline Nuttall,[3] Gareth Griffiths,[3] Alison Layton,[8] Miriam Santer,[2]

[1] Health Economics Group, Norwich Medical School, University of East Anglia, Norwich, UK

[2] Primary Care Research Centre, School of Primary Care, Population Sciences and Medical Education, Faculty of Medicine, University of Southampton, Southampton, UK

[3] Southampton Clinical Trials Unit, University of Southampton and University Hospital Southampton NHS Foundation Trust, Southampton, UK

[4] Centre for Evaluation and Methods, Wolfson Institute of Population Health, Faculty of Medicine and Dentistry, Queen Mary University of London, London, UK

[5] Centre of Evidence Based Dermatology, School of Medicine, University of Nottingham, Nottingham, UK

[6] Public contributor, UK

[7] Population Health Sciences, Bristol Medical School, University of Bristol, Bristol, UK

[8] Skin Research Centre, Hull York Medical School, University of York, York, UK

***Corresponding author:** Professor Tracey Sach, Professor in Health Economics, School of Primary Care, Population Sciences and Medical Education, Faculty of Medicine, University of Southampton, Southampton, UK. Email: T.sach@Soton.ac.uk

FUNDING SOURCES

This study presents independent research funded by the National Institute for Health and Care Research (NIHR) under its Health Technology Assessment programme (16/13/02). The views expressed are those of the author(s) and not necessarily those of the NHS, the NIHR or the Department of Health and Social Care.

This trial was registered prospectively with the ISRCTN registry (ISRCTN12892056) and EudraCT (2018-003630-33).

For peer review only - http://bmjopen.bmj.com/site/about/guidelines.xhtml

CONFLICTS OF INTEREST

We declare no support from any organisation other than the NIHR for the submitted work; no financial relationships with any organisations that might have an interest in the submitted work; no other relationships or activities that could appear to have influenced the submitted work.

LH has received consultancy fees from the University of Oxford on an educational grant funded by Pfizer, unrelated to the submitted work. THS was a member of NIHR HTA Efficient Study Designs - 2, HTA Efficient Study Designs Board, HTA End of Life Care and Add-on-Studies, HTA Primary Care Themed Call Board and the HTA Commissioning Board between 2013 to Dec 2019. She is a steering committee member of the UK Dermatology Clinical Trials Network and Chair of the NIHR Research for Patient Benefit Regional Advisory Panel for the East of England. THS had no part in the decision making for funding this study.

ABSTRACT (300/300 WORDS)

Objective

Acne is common, can cause significant negative impacts and is frequently treated with prolonged courses of oral antibiotics. Despite little clinical and economic evidence spironolactone is prescribed for persistent acne in adult women. This study aims to estimate the cost-effectiveness of oral spironolactone for persistent acne in adult women from a British NHS perspective over 24-weeks.

Design

Economic evaluation undertaken alongside a pragmatic, parallel, double-blind, randomised trial.

Setting

Primary and secondary healthcare, community and social media advertising.

Participants

Women ≥18 years with persistent facial acne judged to warrant oral antibiotic treatment.

Interventions

Participants were randomised 1:1 to 50 mg/day spironolactone (increasing to 100mg/day after 6 weeks) or matched placebo until week-24. Participants in both groups could continue topical treatment use. This economic evaluation compared spironolactone plus routine topical treatment to no active systemic treatment plus routine topical treatment.

Main outcome measures

Cost utility analysis assessed incremental cost per Quality-Adjusted Life Year (QALY) using the EQ-5D-5L. Secondary cost effectiveness analysis estimated incremental cost per unit change on the Acne-QoL symptom subscale. Adjusted analysis included randomisation stratification variables (centre, baseline severity [IGA <3 versus ≥3]), and baseline variables (including Acne-QoL symptom subscale score, resource use costs, EQ-5D score and use of topical treatments (Y/N)).

For peer review only - http://bmjopen.bmj.com/site/about/guidelines.xhtml

Results

Spironolactone did not appear cost-effective in the complete case analysis (n=126 spironolactone and n=109 control), compared with no active systemic treatment (adjusted incremental cost per QALY £67,191; unadjusted £34,770). Incremental cost per QALY was £27,879 (adjusted), just below the upper National Institute of Health and Care Excellence's (NICE) threshold value of £30,000, where multiple imputation took account of missing data. Sensitivity analysis comparing spironolactone to oral antibiotic control gave an adjusted incremental cost per QALY of £9,169. The adjusted incremental cost per point change on the Acne-QoL symptom subscale for spironolactone compared with no active systemic treatment was £38.21 (complete case analysis).

Conclusions

The results demonstrate a high level of uncertainty, particularly with respect to estimates of incremental QALYs. Compared with no active systemic treatment, spironolactone was estimated to be marginally cost-effective where multiple imputation was performed but was not cost-effective in complete case analysis. Participants entering the trial all warranted systemic treatment, making standard of care (oral antibiotics) a more appropriate comparator; against which cost-effectiveness of spironolactone appears favourable, and avoids wider societal costs of over-use of antibiotics.

STRENGTHS AND LIMITATIONS

- Our study is based on individual patient level data collected alongside the first large pragmatic, parallel, double-blind, randomised trial of spironolactone for acne.
- In addition to the base-case analysis seeking to answer the question of whether spironolactone is cost-effective compared with no active systemic treatment (both groups could use routine topical treatments) in women with persistent acne, a number of sensitivity analyses were undertaken to provide a range on estimates of cost-effectiveness under different scenarios.
- Differential rates of missing data between groups over time were addressed by undertaking both a complete case analysis and multiple imputation to explore the impact of missing data on the study conclusions.
- As the study was constrained by the design of the clinical trial, the base-case did not reflect real-world prescribing in the comparator group and evidence to support our sensitivity analysis (comparing to oral antibiotics plus routine topical treatment) is lacking, limiting interpretation of the results.
- The results reflect the method of data collection and may have been limited as a consequence of resource-use under-reporting, short time-frame and limited sensitivity of the EQ-5D outcome measure in patients with acne.

INTRODUCTION

Acne (acne vulgaris) is a common condition, affecting >80% of people at some point in their life.[1] Its impact on the NHS is considerable, being responsible for around 3.5 million consultations with a GP [1] and 70,000 referrals for specialist care[2] in the UK annually. As well as direct burdens to the NHS,

For peer review only - http://bmjopen.bmj.com/site/about/guidelines.xhtml

adults (18–30 years) with severe acne in the UK have higher unemployment rates[3] and a small study by Jowett and Ryan (1985)[4] showed that 45% (13/29) of acne patients reported interpersonal difficulties at work.

There are many treatment options for women with moderate-to-severe acne, but a recent network meta-analysis (NMA) demonstrated paucity of good quality evidence and the complexity of choice.[5] Informed in large part by this NMA and the associated economic model,[6] the National Institute of Health and Care Excellence (NICE) guidelines on the management of acne vulgaris recommend a fixed combination topical preparation containing retinoids, benzoyl peroxide or antibiotics as first-line treatment for any severity of acne, whilst a fixed combination topical agent plus oral lymecycline or doxycycline once daily is recommended for moderate-to-severe acne. The latter is also recommended for moderate-to-severe acne that does not respond adequately to a 12-week course of treatment that does not include an oral antibiotic.[7] The guidance states that treatment options including an antibiotic (topical or oral) should only be continued for more than 6 months in exceptional circumstances (other guidelines limit oral antibiotic duration to 3 months)[8–10] and that clinicians should be aware of the associated risks of antimicrobial resistance. Doctors, however, report many challenges when trying to discontinue oral antibiotics.[11]

Spironolactone is already used off license for women with acne, is an inexpensive treatment choice and could play a role in reducing antibiotic use.[12] Literature searches did not, however, find any previously published economic evaluations on the cost-effectiveness of spironolactone in this group of patients, although there are two other ongoing studies of spironolactone in France and the USA, the former of which includes an economic evaluation.[13,14] In this paper we estimate the cost-effectiveness of spironolactone plus routine topical treatment compared to no active systemic treatment plus routine topical treatment for persistent acne in adult women.

PATIENTS AND METHODS

The Spironolactone for Adult Female Acne (SAFA) trial was a pragmatic, multicentre, participant-led, and clinician-blind, superiority, randomised trial with two parallel treatment groups: spironolactone compared to placebo in women aged 18 years and older with facial acne judged to warrant oral antibiotics. The economic evaluation was nested within this trial.

Participants were recruited in primary care, secondary care and through advertising (community and social media). Baseline assessment was conducted by a research nurse and/or dermatologist in secondary care clinics to ensure standard clinical assessments, as the Investigator's Global Assessment (IGA) for acne was an inclusion criterion and an important secondary outcome. Baseline appointments included a pregnancy test, blood test (to exclude renal impairment or raised serum potassium), participant photo to aid recall about changes in acne and contraceptive counselling. The first participant was recruited in June 2019 and the last in August 2021, whilst follow-up finished February 2022. The SAFA trial is described in more detail in the clinical paper.[15,16]

Participants were randomised 1:1 using online software to either 50 mg/day spironolactone or matched placebo until week-6, increasing to 100 mg/day spironolactone or matched placebo until week-24, assuming treatment was tolerated. Participants were stratified by recruitment centre and baseline acne severity (IGA<3 vs IGA≥ 3). In both groups participants could continue using topical treatment. Between baseline and week-12 participants were asked not to take oral treatment for

For peer review only - http://bmjopen.bmj.com/site/about/guidelines.xhtml

acne other than study medication, except for oral contraception taken for over 3 months previously. After 12 weeks, participants in both groups could receive usual care, including oral treatments, such as oral antibiotics, hormonal treatment or isotretinoin. In both groups participants were followed up face-to-face (or by video call or telephone due to COVID-19) at week-6 and week-12 in secondary care, with primary outcome assessment at week-12, and longer-term follow-up by questionnaires at week-24.

Although in the clinical trial, spironolactone plus routine topical treatment was compared to placebo plus routine topical treatment, it is most appropriate in economic evaluations to compare an active treatment to current usual care.[17] Therefore, to utilise the data collected in the trial whilst reflecting a useful analysis to decision makers in practice, this economic evaluation compared spironolactone plus routine topical treatment to not active systemic treatment plus routine topical treatment.

Measuring costs

In keeping with an NHS perspective, all acne-related resource use data, including intervention, primary and secondary care visits, and prescription medication use, were collected for participants in both groups. Personal Social Services (PSS) resource use was not collected, as patient and clinician contributors did not anticipate these being incurred by participants.

Resource use data was collected via case report forms and participant questionnaires (see supplementary material Appendix S1 for a copy), designed with the input of public contributors, at baseline (collecting the preceding 6 weeks), week-6, week-12 and week-24 for the intervention phase.

Resource uses were valued using UK unit costs (£ Sterling) for the most current price year available at the start of analysis (financial year 2021) and identified from published sources.

The intervention was costed as described in

Figure 1, which assumes that standard treatment with spironolactone, if adopted, will be delivered in primary care, including two GP visits (unless >45 years of age), baseline blood test and the cost of spironolactone (50 mg 6 weeks, 100 mg 18 weeks).[10,18–20] No intervention costs (placebo tablets, GP visits to prescribe placebo tablets or blood tests) were included for the no active systemic treatment group as these would not occur if no intervention was being given (the comparator for this economic evaluation).

Acne-related resource use data related to visits to community-based healthcare professionals (HCP), visits to hospital out-patient and in-patient services (including accident and emergency) and prescribed medication costs were self-reported via participant questionnaires at all time-points, including baseline for participants in both groups. When asked about medication use, participants were asked to report only what they had been prescribed since the previous follow-up visit. Unit costs for each visit-type were combined with this data to estimate the total community-based HCP visit costs and the total hospital contact costs. Participants were also asked for details of prescribed acne-related medication including type, strength and quantity. Unit costs for all medication types[21] were used to estimate the prescription costs over the 24-week treatment period.

The mean (sd) cost per participant per intervention group was estimated for the 24-week treatment period, for each of the cost types described above and mean difference (95% CI) in NHS cost was estimated.

For peer review only - http://bmjopen.bmj.com/site/about/guidelines.xhtml

Measuring outcomes

The primary economic outcome measure was QALYs over the trial period of 24 weeks, as measured by the generic preference-based EQ-5D-5L questionnaire.[22] Responses were converted to utility scores using the EQ-5D-5L Crosswalk UK preference weights, as this was in line with recommendations at the point analysis started, where utility ranges from -0.594 to 1.[23,24] Utility values were used to estimate QALYs over 24 weeks, using both linear interpolation and area under the curve analysis.[25]

A secondary economic outcome was the Acne-QoL symptom sub-scale score (five questions with seven responses to each)[26,27] at week-24, used as an estimate of effectiveness, which enables comparison with future economic studies in acne.

Economic analysis

The base-case cost-utility analysis (CUA) and secondary cost-effectiveness analysis (CEA) incorporated all randomised participants with complete cost and outcome data. Given the 24-week time-horizon, costs and benefits were not discounted.[24]

The base-case CUA estimated the incremental cost per QALY (incremental cost-effectiveness ratio, ICER) to enable comparison with the cost-utility of other interventions. The incremental cost (95% CI) and QALY change (95% CI) between groups was estimated unadjusted and adjusted for randomisation stratification variables (centre, baseline severity [IGA <3 versus ≥3]), and baseline variables (including Acne-QoL symptom subscale score, resource use costs, EQ-5D score and use of topical treatments (Y/N)). In line with NICE guidance,[24] we estimated whether the intervention was cost-effective by comparing the ICER with a cost-effectiveness threshold of £20,000 to £30,000 per QALY.

A CEA estimated the incremental cost per unit change on the Acne-QoL symptom sub-scale score. The incremental cost (95% CI) and Acne-QoL symptom sub-scale change (95% CI) between groups was estimated unadjusted and adjusted as described for the base-case CUA. The CUA and CEA were undertaken using a regression-based approach (seemingly unrelated regression equations).[28]

Published guidelines for the economic evaluation of health care interventions were followed as appropriate.[29,30]

To estimate the level of uncertainty associated with the decision regarding cost-effectiveness, Fieller's theorem was used to calculate [31] the probability of being cost-effective at the £20,000 and £30,000 willingness-to-pay threshold values.[24] Non-parametric bootstrapping was conducted to generate 10,000 estimates of incremental costs and benefits. From this, Cost-Effectiveness Acceptability Curves (CEACs) were generated to show the probability that the intervention is estimated to be cost-effective at different willingness-to-pay values.

Several sensitivity analyses were agreed upon before analysis and undertaken to explore key uncertainties around important parameters in the economic evaluation. The impact of missing data on cost-effectiveness estimates was explored by undertaking multiple imputation, assuming that the data was missing at random (MAR) and using chained equations to handle the missing cost and outcome data.[31] Secondly, the CUA was repeated assuming that, as this patient population had persistent acne of sufficient severity to warrant treatment with oral antibiotics, all women in the no

For peer review only - http://bmjopen.bmj.com/site/about/guidelines.xhtml

active systemic treatment group took oral antibiotics (lymecycline or doxycycline, 1 tablet daily for 24 weeks) as per NICE guidance[32] , in addition to topical treatment. To cost this intervention the weighted mean cost per dose of doxycycline/lymecycline was used (Table 1) and two GP visits assumed. We assumed that the health-related quality of life remained unchanged from what was collected in the SAFA trial, even though incremental QALYs are likely to be lower with effective antibiotic use. A threshold analysis was, therefore, performed to ascertain what level of incremental QALYs would switch the intervention between cost-effective and not cost-effective. The societal costs of over prescribing of oral antibiotics were not included. Further sensitivity analyses in which perspective and delivery of intervention are varied and a sub-group analysis based on age is presented in supplementary material appendix S2. Further details and additional sensitivity analyses and sub-group analysis are presented in online supplementary material appendix S2.

Stata MP version 17 was used to conduct the analyses. A health economic analysis plan was written and followed; a copy is available from the corresponding author.

Patient and public involvement (PPI)

Key questions relating to research design were explored with a virtual acne-specific patient panel and patient survey carried out via the UK Dermatology Clinical Trials Network (UKDCTN). Two public contributors (IS and KaT) with experience of acne were members of the Trial Management group as part of this role they helped identify relevant resources and outcomes and how this data should be collected. They also contributed to the interpretation and write-up if the health economics component.

RESULTS

Participant characteristics

The clinical trial results, including details on sample size and participant characteristics, are reported elsewhere.[16] Of the 410 women recruited to the trial, 201 were randomly assigned to spironolactone and 209 allocated to placebo at the start of the trial. All were allowed to continue routine topical treatment. At week-24 126 women in the spironolactone group and 109 women in the placebo group had complete cost and outcome data, and these formed the base-case unadjusted CUA. Mean age was 29.2 years, mean BMI was 26.1, at baseline 83% (340/410) participants were using or had used topical treatments, and the majority (75% [306/410]) had acne for two or more years. There were no significant differences in characteristics between groups.[16]

Costs

The Unit costs used in the analysis are presented in Table 1. The levels of resource use in each group were very similar prior to randomisation (Table S1).

The majority of responding women in the spironolactone group (182/184, 99%) increased to two tablets of spironolactone at week 6. The ‘standard treatment’ approach used in the base-case economic evaluation, gave rise to a mean total intervention resource use cost of £122.87 (SD £13.04) per participant in the spironolactone group (Table 2).

Using available case data, when intervention use was combined with other health resource use, the unadjusted mean incremental cost per participant was £126.35 (95% CI, £112.88 to £139.82) for women receiving spironolactone compared to women receiving no active systemic treatment in the

For peer review only - http://bmjopen.bmj.com/site/about/guidelines.xhtml

base-case (Table 2). Excluding intervention costs, the difference was not significant between groups. While patients were asked about in-patient visits, none were reported.

Outcomes

The mean (sd) QALYs over 24 weeks in the spironolactone group were 0.417 (0.058) per participant compared to 0.404 (0.079) per participant in the no active systemic treatment group, giving an incremental difference of 0.013 (95% CI -0.0024 to 0.0289) QALYs using unadjusted available case data (Table 2). The wide 95% confidence intervals around mean estimates demonstrates a high degree of uncertainty.

The mean (sd) change from baseline in Acne-QoL symptom subscale score at 24 weeks was 8.15 (6.12) in the spironolactone group compared to 4.46 (6.34) in the no active systemic treatment group. Thus, the incremental difference in score was 3.68 (95% CI 2.26 to 5.11) in favour of the spironolactone group (Table 2).

Base-case Cost Utility Analysis

In the complete case analysis, the incremental cost for the spironolactone group (n=118) compared to the no active systemic treatment group (n=101) was £125.36 (95% CI, £111.13 to £139.58) (unadjusted this was £125.53 [95% CI £112.15 to £138.91]) (Table 3). The adjusted incremental QALYs for the spironolactone group compared with the no active systemic treatment group was 0.0019 (95% -0.0096 to 0.0133) (unadjusted was 0.0036, 95% CI -0.0117 to 0.0189). The ICER was £67,191 (unadjusted £34,770) per QALY. At a willingness to pay of £30,000 per QALY there was a 35% (unadjusted 47%) chance of spironolactone being cost-effective in this population of women with persistent acne.

The cost-effectiveness acceptability curves (**Error! Reference source not found.**) of the adjusted and unadjusted base-case analysis, show that the probability of spironolactone being cost-effective only approaches 50% as the threshold value approaches £120,000 (adjusted), demonstrating a high degree of uncertainty associated with the decision under these conditions.

Secondary Cost Effectiveness analysis

The adjusted incremental difference in cost per point change on the Acne-QoL symptom subscale for the spironolactone group (n=119) compared to no active systemic treatment group (n=102) was £38.21 (unadjusted £35.91) based on a complete case analysis (Table). How much a decision maker would be willing to pay for a point change on the Acne-Qol symptom subscale is unknown.

Sensitivity analyses

There were differential rates of attrition with greater missing data in the no active systemic treatment group, compared to spironolactone group, by 24-weeks follow-up, for costs (39% vs. 24%, respectively) and EQ-5D-5L (33% vs. 20%, respectively). When using multiple imputation in a sensitivity analysis the ICER was less than in the complete case, adjusted analysis (£27,879 compared with £67,191 per QALY) with a 53% probability of spironolactone being cost effective at £30,000 per QALY (Table 3).

There were 10 participants who reported taking an oral antibiotic (lymecycline/oxytetracycline/ doxycycline) between baseline and week 24, 6 participants in the no active systemic treatment group and 4 participants in the spironolactone group. In a sensitivity analysis assuming all women in

For peer review only - http://bmjopen.bmj.com/site/about/guidelines.xhtml

the no active systemic treatment group took an oral antibiotic for the 24-week treatment period,[32] an ICER of £9,169 per QALY was estimated (complete case analysis) with a 59% probability of spironolactone being cost-effective at a £30,000 per QALY threshold. With multiple imputation, the ICER was £2,683 per QALY with an 82% probability that spironolactone would be cost effective at a £30,000 threshold (Table 3). Threshold analysis using the complete case analysis data found that incremental QALYs would have to decrease to 0.00057 to switch the ICER from being cost-effective to not cost-effective at a £30,000 threshold.

The results of additional sensitivity analyses and a subgroup analysis based on age are reported in online supplementary material appendix S2. In particular, a wider perspective is taken including participant and carer out of pocket and productivity costs (see Table S2) and a sensitivity analysis is taken costing the intervention resource use as delivered via secondary care per trial protocol (see Figure S1). A sub-group analysis is undertaken for women aged <25 years and ≥25 years. See Table S3 for results.

DISCUSSION

This economic study finds a high degree of uncertainty about whether spironolactone is likely to be cost-effective. Our economic evaluation provides a range of estimates for the cost effectiveness of spironolactone used alongside routine topical treatment. The base-case analysis, where the comparator is no active systemic treatment plus routine topical treatment, and the delivery of the intervention is costed as via primary care, spironolactone was not estimated to be cost-effective in the unadjusted and adjusted complete case analyses. However, in the adjusted analysis using multiple imputation (MI) the ICER was estimated to be just under the £30,000 per QALY threshold. This divergence in conclusion between the complete case and MI analysis demonstrates the impact of missing data (attrition bias) and suggests more weight ought to be placed on the MI analysis.[33] In sensitivity analyses, where spironolactone plus routine topical treatment is compared to oral antibiotic plus routine topical treatment (standard of care based upon NICE guidelines) based on the assumptions outlined in the methods section, spironolactone is found to be cost-effective. Threshold analysis around the level of incremental QALYs assumed in this analysis, demonstrates that the conclusion is likely to hold true even if the incremental QALYs gained in the spironolactone group compared with the oral antibiotic group are decreased to 0.00057. We await further evidence to determine whether this is a likely scenario.[13]

This economic evaluation followed a Health Economic Analysis Plan finalised before data was received for analysis reducing bias in the results from selective reporting or cherry-picked analyses.[34] Another strength of this economic evaluation is that it can provide reliable estimates of cost effectiveness based on individual participant level data, collected at little marginal cost, alongside a randomised controlled trial. This is, however, also a limitation in that within trial health economic evaluations are constrained by the question, timeframe, and data collected, particularly in placebo-controlled trials. In particular there are four main limitations to acknowledge: (1) the assumptions required to compare spironolactone to inactive systemic treatment; (2) the assumptions required to undertake a sensitivity analysis using oral antibiotics as the comparator; (3) the sensitivity and validity of the EQ-5D-5L in patients with acne; (4) the time frame of the analysis; and (5) the use of complete case analysis rather than the analysis using multiple imputation to take account of missing

For peer review only - http://bmjopen.bmj.com/site/about/guidelines.xhtml

data as the base case analysis. We look at these in turn below, but all should be borne in mind when interpreting the results.

Firstly, ideally economic evaluations should compare an active treatment to current usual care. The funder for this trial preferred the placebo comparator to current usual care.[17] We wanted our primary analysis to reflect as closely as possible the data collected in the actual trial whilst reflecting a useful analysis to decision making in practice. We therefore felt the most appropriate comparator would be no active systemic treatment, rather than placebo, which would not reflect reality. Placebos are not used in routine practice, but some evidence of placebo effects has been documented in acne.[5] Therefore, the base-case set out to answer the question of whether spironolactone is cost-effective compared with no active systemic treatment (both groups could use routine topical treatments) to align with the clinical question funded. A limitation of this is that, because it does not account for the potential impact of a placebo effect, it may result in underestimation of the QALY gain with spironolactone compared with not providing spironolactone, and hence underestimate its cost-effectiveness.. We also excluded the research costs associated with administering the placebo (costs of the pills and appointments to administer them) but did including ongoing costs associated with NHS resource use related to acne in both arms of the study. It is not possible to know how costs and effects would differ between our placebo group and a group without any active systemic treatment because we did not have the latter group in the study. We feel the assumptions made are required to make the analysis most useful to practice but acknowledge they may mean the estimates of the cost-effectiveness of spironolactone are conservative.

Secondly, in practice clinicians are unlikely to send women away with no active treatment if they consulted with acne persisting beyond 6 months. The clinically important comparator may be another systemic treatment rather than no active systemic treatment. To address this a sensitivity analysis assuming, for cost purposes, all women in the no active systemic treatment group received an oral antibiotic (in addition to topical treatments) for 24 weeks was performed. This analysis assumed that incremental QALYs remain the same as in the base-case analysis, which we acknowledge is unlikely. There is limited economic evidence comparing oral antibiotics in combination with routine topical treatment compared with routine topical treatment alone, but a network meta-analysis[5] did not rank oral tetracycline treatment (alone or in combination) higher than topical combination treatment. Despite these limitations and while the results of this sensitivity analysis should be interpreted with caution, considering the assumptions made, the analysis serves to provide a lower range estimate for the cost effectiveness of spironolactone that better reflects accepted standard-of-care, based upon current NICE guidelines.[32]

Thirdly, the uncertainty highlighted by this study may be impacted, in part, by the method of measuring utility, an area where further research would be valuable. The conclusion reached about cost-effectiveness was sensitive to the estimates of QALYs generated from EQ-5D-5L, despite 46% in the intervention group and 43% in the control group reporting perfect health (EQ-5D-5L health state 11111) at baseline. For these participants, the EQ-5D-5L had no potential to measure improvements in health-related quality of life. This likely contributes to the wide 95% confidence intervals around the incremental QALY estimates in this study, which means we cannot be certain spironolactone improves QALYs rather than have no difference or worsen QALYs. At design stage, there was discussion about the possible use of other instruments, however, the limited published evidence

For peer review only - http://bmjopen.bmj.com/site/about/guidelines.xhtml

supported the use of the EQ-5D for acne.[35,36] Like Klassen et al [36] we find that women with persistent acne report most problems on the pain/discomfort and anxiety/depression dimensions of the EQ-5D. Further research using the EQ-5D data generated in this study alongside that elicited in other studies of acne would help inform future studies about the validity and responsiveness of this instrument for acne.

Fourthly, we acknowledge that the analysis was conducted for a 24-week timeframe and that were a longer timeframe taken the cost-effectiveness of spironolactone may improve if, for instance, there is a sustained effect once treatment stops. We sought to collect resource use and utility data up to 52 weeks but due to reduced data completion at 52 weeks (see supplementary material for details) it was not feasible to analyse results to a longer time horizon. [32]

Finally, a complete case analysis was specified in the Health Economic Analysis Plan as the base case analysis (with multiple imputation as a sensitivity analysis) reflecting a desire to be consistent with the approach undertaken in the Statistical Analysis Plan for the clinical primary outcome. With the benefit of hindsight primary concern ought to have been around the level of missing economic data, which is known to often be greater than that for clinical outcomes. However, both complete case and multiple imputation analyses are reported, as planned, so that the impact of missing data on the results can be clearly seen.

Our study provides estimates of the cost-effectiveness of spironolactone in women with persistent acne using the trial data and a range of scenarios. It highlights that there is uncertainty about whether spironolactone is cost effective and the need for further research with comparators more akin to clinical practice. The complete case analysis estimated ICERs in excess of the upper NICE threshold of £30,000 per QALY but this analysis took a conservative approach since it may be that incremental QALYs for spironolactone would have been greater had we been able to control for any placebo effect and had more complete data beyond 24 weeks. When taking into account missing data the ICER was below the upper NICE threshold suggesting spironolactone may be considered cost-effective. However, all analyses show a high degree of uncertainty suggestive of a need for further research to reduce the uncertainties.

ACKNOWLEDGEMENTS

We would like to thank all PPI contributors, participants, research and clinical staff, the NIHR Clinical Research Network, and the members of the Trial Steering Committee and Data Monitoring Committee for their support.

The study was developed with support from the UK Dermatology Clinical Trials Network (UK DCTN). The UK DCTN is grateful to the British Association of Dermatologists and the University of Nottingham for financial support of the Network.

The University of Southampton was the research sponsor for this trial.

CONTRIBUTORS

MS, AL, BS, THS, MJR, NF, KST, PL, JN, GG and IM conceived the study idea and initial study design in response to a NIHR HTA call, with later input from KLT, IS, ZE, SR, ML, NP and SP. All authors contributed to the acquisition of data. Specific advice was given by BS on trial design and medical

For peer review only - http://bmjopen.bmj.com/site/about/guidelines.xhtml

statistics; and THS on health economic evaluation. Economic analyses were conducted by SP and THS. All authors contributed to the interpretation of data and drafting of this paper, led by SP and THS, and approved the final manuscript.

DATA AVAILABILITY

Consent was not obtained from participants for data sharing but authors will consider reasonable requests to make relevant anonymised participant level data available via the Southampton Clinical Trials Unit Data Sharing Committee.

ETHICS STATEMENT

This study was conducted in compliance with the ethical principles of the Declaration of Helsinki and in compliance with all International Council for Harmonisation Good Clinical Practice guidelines. The study protocol was reviewed and approved by the Institutional Review board and/or Independent Ethics Committee at each participating centre. All participants provided written informed consent.

Ethical approval for the trial was given by Wales Research Ethics Committee (REC) 3 in January 2019 (18/WA/0420).

SUPPORTING INFORMATION

Additional supporting information may be found in the online version of this article at the publisher's website:

Appendix S1: Participant Resource Use Questionnaire

Appendix S2 Supplementary material: further sensitivity and sub-group analyses

Figure S1 Intervention resource use as delivered via secondary care, per trial protocol

Table S1 Estimates of mean baseline resource use by treatment group (available case data)

Table S2 Estimates of mean change in cost (UK£ 2021/22) including wider costs, by treatment group

Table S3 Cost utility analyses and cost-effectiveness analyses results, for additional sensitivity analyses and sub-group analysis

For peer review only - http://bmjopen.bmj.com/site/about/guidelines.xhtml

REFERENCES

1. Purdy S, de Berker D. Acne. *BMJ* 2006; 333: 949–953.

2. Schofield J, Grindlay D, Williams H. *Skin conditions in the UK: a Health Care Needs Assessment*. University of Nottingham, 2009.

3. Cunliffe WJ. Acne and unemployment. *Br J Dermatol* 1986; 115: 386–386.

4. Jowett S, Ryan T. Skin disease and handicap: an analysis of the impact of skin conditions. *Soc Sci Med* 1985; 20: 425–9.

5. Mavranezouli I, Daly CH, Welton NJ, et al. A systematic review and network meta-analysis of topical pharmacological, oral pharmacological, physical and combined treatments for acne vulgaris. *Br J Dermatol* 2022; 187: 639–649.

6. Mavranezouli I, Welton NJ, Daly CH, et al. Cost-effectiveness of topical pharmacological, oral pharmacological, physical and combined treatments for acne vulgaris. *Clin Exp Dermatol*. Epub ahead of print 30 July 2022. DOI: 10.1111/ced.15356.

7. NICE. *Acne vulgaris: management NICE guideline*, www.nice.org.uk/guidance/ng198 (2021, accessed 16 November 2022).

8. le Cleach L, Lebrun-Vignes B, Bachelot A, et al. Guidelines for the management of acne: recommendations from a French multidisciplinary group. *Br J Dermatol* 2017; 177: 908–913.

9. Thiboutot DM, Dréno B, Abanmi A, et al. Practical management of acne for clinicians: An international consensus from the Global Alliance to Improve Outcomes in Acne. *J Am Acad Dermatol* 2018; 78: S1-S23.e1.

10. Zaenglein AL, Pathy AL, Schlosser BJ, et al. Guidelines of care for the management of acne vulgaris. *J Am Acad Dermatol* 2016; 74: 945-973.e33.

11. Platt D, Muller I, Sufraz A, et al. GPs' perspectives on acne management in primary care: a qualitative interview study. *Br J Gen Pract* 2020; 71: E78–E84.

12. Layton AM, Eady EA, Whitehouse H, et al. Oral Spironolactone for Acne Vulgaris in Adult Females: A Hybrid Systematic Review. *Am J Clin Dermatol* 2017; 18: 169–191.

13. Barbieri Lab. Spironolactone versus doxycycline for acne: a comparative non-inferiority evaluation (SD-ACNE) research study, https://barbierilab.bwh.harvard.edu/clinical-trial-opportunities/ (accessed 17 November 2022).

14. Poinas A, Lemoigne M, le Naour S, et al. FASCE, the benefit of spironolactone for treating acne in women: study protocol for a randomized double-blind trial. *Trials* 2020; 21: 571.

15. Renz S, Chinnery F, Stuart B, et al. Spironolactone for adult female acne (SAFA): protocol for a double-blind, placebo-controlled, phase III randomised study of spironolactone as systemic therapy for acne in adult women. *BMJ Open* 2021; 11: e053876.

For peer review only - http://bmjopen.bmj.com/site/about/guidelines.xhtml

16. Santer M, Lawrence M, Renz S, et al. Effectiveness of spironolactone for women with acne vulgaris (SAFA) in England and Wales: pragmatic, multicentre, phase 3, double blind, randomised controlled trial. *BMJ 2023;381:e074349.*

17. Drummond M, Sculpher M. Common methodological flaws in economic evaluations. *Med Care* 2005; 43: 5–14.

18. Thiede RM, Rastogi S, Nardone B, et al. Hyperkalemia in women with acne exposed to oral spironolactone: A retrospective study from the RADAR (Research on Adverse Drug Events and Reports) program. *Int J Womens Dermatol* 2019; 5: 155–157.

19. Plovanich M, YuWeng Q, Mostaghimi A. Low Usefulness of Potassium Monitoring Among Healthy Young Women Taking Spironolactone for Acne. *JAMA Dermatol* 2015; 151: 941–944.

20. Wang Y, Lipner SR. Retrospective analysis of adverse events with spironolactone in females reported to the United States Food and Drug Administration. *Int J Womens Dermatol* 2020; 6: 272.

21. NHS Business Service Authority. *Prescription Cost Analysis - England 2020/21*, https://www.nhsbsa.nhs.uk/statistical-collections/prescription-cost-analysis-england/prescription-cost-analysis-england-202021 (2021, accessed 30 November 2022).

22. Herdman M, Gudex C, Lloyd A, et al. Development and preliminary testing of the new five-level version of EQ-5D (EQ-5D-5L). *Qual Life Res* 2011; 20: 1727–36.

23. van Hout B, Janssen MF, Feng YS, et al. Interim scoring for the EQ-5D-5L: Mapping the EQ-5D-5L to EQ-5D-3L value sets. *Value in Health* 2012; 15: 708–715.

24. NICE. Guide to the methods of technology appraisal 2013. *National Institute for Health and Care Excellence* 2013; 1–93.

25. Manca A, Hawkins N, Sculpher MJ. Estimating mean QALYs in trial-based cost-effectiveness analysis: The importance of controlling for baseline utility. *Health Econ* 2005; 14: 487–496.

26. Fehnel SE, McLeod LD, Brandman J, et al. Responsiveness of the Acne-Specific Quality of Life Questionnaire (Acne-QoL) to treatment for acne vulgaris in placebo-controlled clinical trials. *Qual Life Res* 2002; 11: 809–816.

27. Martin AR, Lookingbill DP, Botek A, et al. Health-related quality of life among patients with facial acne -- assessment of a new acne-specific questionnaire. *Clin Exp Dermatol* 2001; 26: 380–385.

28. Willan AR, Briggs AH, Hoch JS. Regression methods for covariate adjustment and subgroup analysis for non-censored cost-effectiveness data. *Health Econ* 2004; 13: 461–475.

29. Drummond M, Sculpher M, Claxton K, et al. *Methods for the economic evaluation of health care programmes*. 4th ed. Oxford, UK: Oxford University Press, 2015.

30. Ramsey SD, Willke RJ, Glick H, et al. Cost-effectiveness analysis alongside clinical trials II - An ISPOR good research practices task force report. *Value in Health* 2015; 18: 161–172.

For peer review only - http://bmjopen.bmj.com/site/about/guidelines.xhtml

For peer review only

31. Faria R, Gomes M, Epstein D, et al. A Guide to Handling Missing Data in Cost-Effectiveness Analysis Conducted Within Randomised Controlled Trials. *Pharmacoeconomics* 2014; 32: 1157–1170.

32. Xu J, Mavranezouli I, Kuznetsov L, et al. Management of acne vulgaris: summary of NICE guidance. *BMJ* 2021; 374: n1800.

33. Leurent B, Gomes M, Faria R, et al. Sensitivity Analysis for Not-at-Random Missing Data in Trial-Based Cost-Effectiveness Analysis: A Tutorial. *Pharmacoeconomics* 2018; 36: 889–901.

34. Thorn JC, Davies CF, Brookes ST, et al. Content of Health Economics Analysis Plans (HEAPs) for Trial-Based Economic Evaluations: Expert Delphi Consensus Survey. *Value in Health* 2021; 24: 539–547.

35. Yang Y, Brazier J, Longworth L. EQ-5D in skin conditions: an assessment of validity and responsiveness. *European Journal of Health Economics* 2015; 16: 927–939.

36. Klassen AF, Newton JN, Mallon E. Measuring quality of life in people referred for specialist care of acne: Comparing generic and disease-specific measures. *J Am Acad Dermatol* 2000; 43: 229–233.

37. Jones K, Burns A. Unit Costs of Health and Social Care 2021. *PSSRU*, https://www.pssru.ac.uk/project-pages/unit-costs/unit-costs-of-health-and-social-care-2021/ (2021, accessed 5 December 2022).

38. NHS England. *2019/2020 National Cost Collection Data Publication*. London, https://www.england.nhs.uk/publication/2019-20-national-cost-collection-data-publication/ (2021, accessed 5 December 2022).

39. Curtis L, Burns A. *Unit costs of health and social care 2015*. Personal Social Services Research Unit, University of Kent, Canterbury, 2015.

40. UKHCA. *UKHCA Commisioning Survey 2012: Care is not a Commodity*. 2012.

41. Brown R. *PSNC Pharmacy Advice Audit 2021*. 2021.

42. Office for National Statistics. Annual Survey of Hours and Earnings time series of selected estimates, https://www.ons.gov.uk/employmentandlabourmarket/peopleinwork/earningsandworkinghours/datasets/ashe1997to2015selectedestimates (2021, accessed 5 December 2022).

For peer review only

For peer review only - http://bmjopen.bmj.com/site/about/guidelines.xhtml

FIGURES

Figure 1 | Intervention resource use as per standard treatment with spironolactone (base-case)

Figure 2 | Cost Effectiveness Acceptability Curve (CEAC), complete case analysis, adjusted and unadjusted QALYs

For peer review only

For peer review only - http://bmjopen.bmj.com/site/about/guidelines.xhtml

TABLES

Table 1 | Unit costs (UK£ sterling, 2020/21 financial year)

Cost Item	Unit Cost (£)	Unit	Source, assumptions
**Intervention**			
Spironolactone with dose escalation	£49.37	Total	Prescription Cost Analysis 2021.[21]
GP visit related to intervention	£33.00	Total	PSSRU Unit costs 2021.[37]
Blood test for renal function (eGFR) and potassium level (K serum)	£5.22	Total	National Cost Collection 2020.[38] *
**Medication costs**	Mean cost per quantity		
Topical preparations for acne	£0.96	gram/ml	Prescription Cost Analysis 2021.[21]<br>Mean across all medications in each medication type. Weighted averages taken where listed >1x.<br>Weighted average for estimating oral antibiotic control for SA (see table 3). Assumes 1x100 mg (doxycycline)/408 mg (lymecycline) per day for 24 weeks.
Other topical preparation	£0.03	gram/ml	
Oral contraceptives	£0.08	tablet	
Oral antibiotics	£0.22	capsule/tablet	
Anti-depressants	£0.20	capsule/tablet	
Analgesics	£0.04	capsule/tablet	
PCOS/diabetes medication	£0.03	tablet	
Other medications	£0.40	various	
Doxycycline/lymecycline weighted average	£0.25	Capsule	
**Community-based HCP contacts**			
GP visit unrelated to intervention	£33.00	Visit	PSSRU Unit costs 2021.[37]
Practice Nurse	£14.13	Visit	PSSRU Unit costs 2021 & 2015. [37,39]
NHS Walk-in centre	£71.99	Visit	National Cost Collection 2020.[38] Weighted average of all community health services.*
Community dermatology service	£121.01	Visit	National Cost Collection 2020.[38] *
Healthcare assistant	£14.44	Visit	PSSRU Unit Costs 2021[37] & UKHCA Commissioning Survey 2012.[40]
Pharmacist	£6.99	Visit	PSSRU Unit costs 2021 & 2015[37,39] & PSNC Pharmacy Advice Audit 2021.[41]
Physiotherapist	£66.82	Visit	National Cost Collection 2020.[38] *
Dietician	£82.46	Visit	National Cost Collection 2020.[38] *
Other (community)	£33.00	Visit	PSSRU Unit costs 2021. Used most common visit: GP visit.[37]
**Hospital out-patient contacts**			
Dermatologist	£128.25	Visit	National Cost Collection 2020.[38] *

For peer review only - http://bmjopen.bmj.com/site/about/guidelines.xhtml

Dermatology Nurse	£100.71	Visit	National Cost Collection 2020.[38] *
Ear, nose and throat (ENT)	£116.11	Visit	National Cost Collection 2020.[38] *
Interventional radiology	£137.64	Visit	National Cost Collection 2020.[38] *
Trauma and orthopaedics	£125.67	Visit	National Cost Collection 2020.[38] *
Respiratory medicine	£161.07	Visit	National Cost Collection 2020.[38] *
Other (out-patient)	£137.10	Visit	National Cost Collection 2020.[38] *
**Hospital admission**			
Accident and emergency	£182.28	Visit	National Cost Collection 2020. Index/Accident & Emergency.[38] *
**Wider costs**			
Personal out-of-pocket expenses	Various	Per item	Participant reported.
Lost work time	£18.01	Hour	ONS 2021.[42] Mean hourly earnings, excluding overtime (£).

SA, sensitivity analysis; ONS, Office for National Statistics; PSSRU, Personal Social Services Research Unit.

* Inflated to 2021 prices as per NHSCII Pay & Prices.[37]

For peer review only - http://bmjopen.bmj.com/site/about/guidelines.xhtml

Table 2 | Estimates of mean change in resource use and cost (UK£ 2021/22) and mean utility and QALY gain by treatment group (based on available case data)

Resource	Spironolactone (N=201)		No active systemic treatment (N=209)		Mean difference
	**Mean (n)**	**SD**	**Mean (n)**	**SD**	**(95% CI)**
Resource use over 24-week period:					
Spironolactone (number)	294 (201)	0	0 (209)	0	-
GP visits related to intervention (no. of visits)*	2.06 (201)	0.34	0 (209)	0	-
Blood tests – renal function (eGFR) and potassium level (number)	1.06 (201)	0.34	0 (209)	0	-
Total community-based HCP visits (number)	0.15 (150)	0.51	0.10 (124)	0.43	0.05 (-0.064 to 0.164)
Total hospital contacts (number)	0.06 (132)	0.30	0.05 (115)	0.26	0.01 (-0.062 to 0.079)
All prescription medications (number)	11.42 (147)	29.65	23.36 (124)	96.80	-11.94 (-28.507 to 4.625)
Costs over 24-week period:	UK£2021/22				
All intervention costs	122.87 (201)	13.04	0 (209)	0	122.87 (121.09 to 124.64)
All community-based HCP costs	6.28 (150)	24.83	3.75 (124)	16.46	2.53 (-2.60 to 7.66)
All hospital contact costs	7.28 (132)	36.42	5.73 (115)	28.09	1.55 (-6.70 to 9.79)
All prescription medication costs	4.37 (147)	11.77	5.91 (124)	18.93	-1.54 (-5.25 to 2.17)
**Total costs**	**141.99 (128)**	**57.90**	**15.64 (110)**	**45.62**	**126.35 (112.88 to 139.82)**
**Total costs excluding intervention**	**19.61 (128)**	**56.65**	**15.64 (110)**	**45.62**	**3.98 (-9.30 to 17.26)**
EQ-5D score (CUA)					
Baseline	0.887 (200)	0.148	0.860 (209)	0.200	0.027 (-0.008 to 0.061)
6 weeks	0.894 (176)	0.135	0.863 (179)	0.168	0.031 (-0.001 to 0.063)
12 weeks	0.904 (174)	0.138	0.877 (166)	0.177	0.027 (-0.007 to 0.061)
24 weeks	0.909 (163)	0.153	0.890 (136)	0.180	0.019 (-0.019 to 0.057)
**Total QALY score over 24 weeks**	**0.417 (162)**	**0.058**	**0.404 (136)**	**0.079**	**0.013 (-0.002 to 0.029)**
Acne-QoL symptom sub-scale score (CEA)					
Baseline	13.22 (201)	4.94	12.87 (209)	4.55	0.35 (-0.57 to 1.27)
6 weeks	16.97 (176)	5.72	15.65 (179)	5.69	1.32 (0.13 to 2.51)
12 weeks	19.21 (176)	6.12	17.76 (166)	5.58	1.45 (0.20 to 2.69)
24 weeks	21.22 (163)	5.86	17.39 (136)	5.80	3.83 (2.49 to 5.16)
**Change at 24-weeks from baseline**	**8.15 (163)**	**6.12**	**4.46 (136)**	**6.34**	**3.68 (2.26 to 5.11)**

*Assumes that if spironolactone is found effective it would be prescribed in primary care.

For peer review only

Table 3 | Cost utility analyses and cost-effectiveness analyses results, including sensitivity analyses and sub-group analysis

CUA Analysis (N s, N p)	Incremental cost (95% CI)	Incremental QALYs (95% CI)	ICER	CEAC at £20,000 (£30,000) threshold*
Base-case^, CCA, adjusted (118,101)	125.36 (111.13 to 139.58)	0.0019 (-0.0096 to 0.0133)	£67,191	23% (35%)
Base-case^, CCA, unadjusted (126,109)	125.53 (112.15 to 138.91)	0.0036 (-0.0117 to 0.0189)	£34,770	37% (47%)
SA^, Multiple imputation, adjusted (201,209)	119.78 (107.99 to 131.57)	0.0043 (-0.0041 to 0.0127)	£27,879	35% (53%)
SA, oral antibiotic control, CCA, adjusted (118,101)	17.11 (2.88 to 31.33)	0.0019 (-0.096 to 0.0133)	£9,169	57% (59%)
SA, oral antibiotic control, MI, adjusted (201, 209)	11.53 (-0.26 to 23.32)	0.0043 (-0.0041 to 0.0127)	£2,683	81% (82%)
**CEA Analysis (N s, N p)**	**Incremental cost (95% CI)**	**Incremental Acne-QoL symptom (95% CI)**	**Incremental cost per unit change**	-
Secondary analysis^, CCA, adjusted: (119,102)	126.57 (112.35 to 140.78)	3.31 (1.90 to 4.72)	£38.21	-
Secondary analysis^, CCA, unadjusted (127,110)	126.52 (113.00 to 140.04)	3.52 (1.94 to 5.11)	£35.91	-

^ comparing spironolactone plus routine topical treatment to no active systemic treatment plus routine topical treatment; 95% CI=95% confidence interval; ICER =incremental cost-effectiveness ratio; N s / N p =Number randomised to spironolactone / Placebo who were included in the analysis; CCA = complete case analysis; SA refers to the different sensitivity analyses described in the Methods; QALY=Quality Adjusted Life Years; *probability of being cost-effective at a the threshold (λ) of £20,000 and £30,000 per QALY. Adjusted analyses, adjusted for stratification variables (centre, baseline severity [IGA<3 vs. ≥3]) and baseline variables (Acne QoL symptom subscale score, use of topical treatments, utility score based on EQ-5D, total costs)

For peer review only

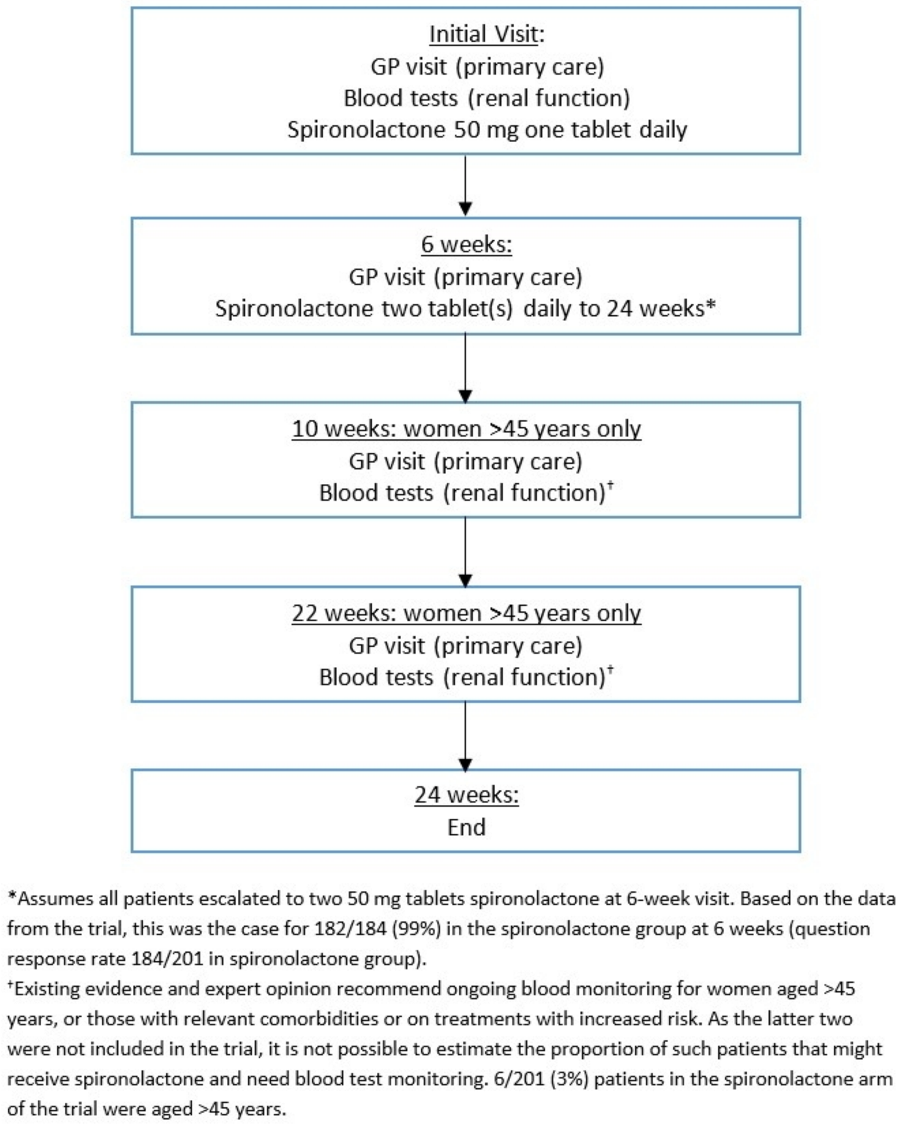

Figure 1: Intervention resource use as per standard treatment with spironolactone (base-case)

190x232mm (96 x 96 DPI)

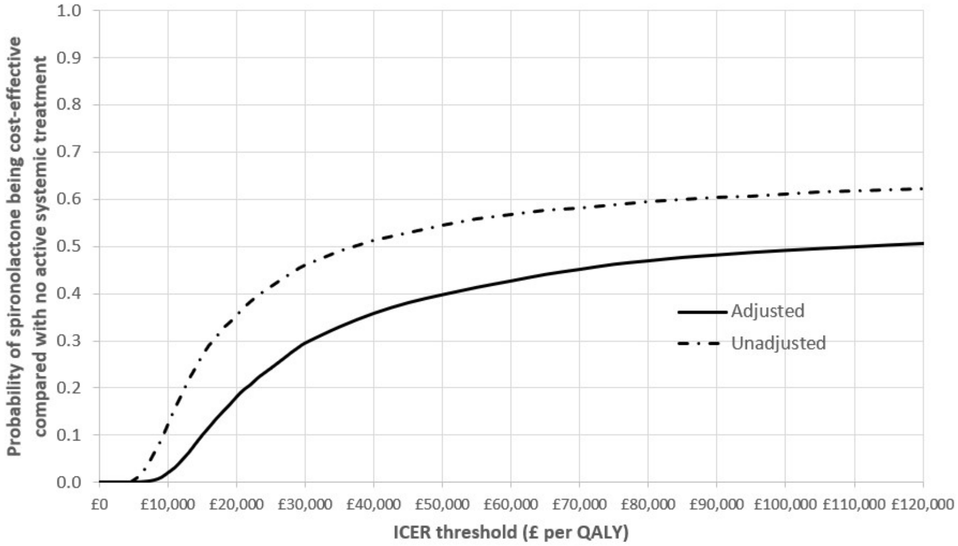

Figure 2: Cost Effectiveness Acceptability Curve (CEAC), complete case analysis, adjusted and unadjusted QALYs

246x145mm (96 x 96 DPI)

SUPPORTING INFORMATION

APPENDIX S1: PARTICIPANT RESOURCE USE QUESTIONNAIRE

The following information is presented in addition to the main paper, "Cost-effectiveness of Spironolactone for Adult Female Acne (SAFA): Economic evaluation alongside a randomised controlled trial", published in XXXX.

The example given below is taken from the SAFA 6-week questionnaire. These questions were part of a wider questionnaire used at 6 weeks.

For peer review only

Participant's initials: | | | |

Participant's study identifier: | | | | | | | | |

SAFA 6 week Questionnaire – Participant

Services received

These questions are about your health and care needs. In the **last 6 weeks** what publically provided services (i.e. those you do not have to pay for out of your own pocket) have you received because of your acne?

If you are unsure, please put in your best estimate.

Question 1: Community-based NHS services

1a. In the **last 6 weeks** have you seen any community-based health professionals (e.g. GP, practice nurse, dietician etc) because of your acne?

☐ Yes ☐ No, if 'No' please go to question 2

Have you seen any of the following health professionals in the **last 6 weeks**? If 'Yes', please tell us how many times. There is space for you to name other professionals you have seen via the NHS and how many time you have visited them. If you did not see any other professionals please tick 'No' in the "Other" rows.

General Practitioner	☐ No	☐ yes	If yes, how many times?	___________
Practice nurse	☐ No	☐ Yes	If yes, how many times?	___________
Health care assistant	☐ No	☐ Yes	If yes, how many times?	___________
NHS Walk in centre	☐ No	☐ Yes	If yes, how many times?	___________
Community dermatology service	☐ No	☐ Yes	If yes, how many times?	___________
Other, please specify: ______________________	☐ No	☐ Yes	If yes, how many times?	___________
Other, please specify: ______________________	☐ No	☐ Yes	If yes, how many times?	___________
Other, please specify: ______________________	☐ No	☐ Yes	If yes, how many times?	___________

For peer review only - http://bmjopen.bmj.com/site/about/guidelines.xhtml

Funded by NIHR

Participant's initials: | | | |

Participant's study identifier: | | | | | | | | |

Question 2: Medication

2. In the **last 6 weeks** have you been **prescribed** any medications because of your acne? (Please include anything that you feel is related to your acne, for example if you take anti-depressants and your depression is mainly because of your acne you would include this).

☐ Yes ☐ No, if 'No' please go to question 3a

If 'Yes', please give the name of the medication, the strength, and size of the item.

Name of medication (item)	Strength	Unit	Number of items	Type of item (e.g. pack, bottle, tube, etc)	Number in item	Size of item
Example 1: Epiduo Gel	2.5	%	2	tubes	12	grams per tube
Example 2: Tetracyclin	250	mg	1	pack	28	tablets per pack

Question 3: Hospital-based services

3a. **In the last 6 weeks** have you visited a hospital as an outpatient because of your acne or side effects from treatment for your acne?

☐ Yes ☐ No, if 'No' please go to question 3b

If 'Yes', for **each outpatient visit** you had at the hospital as a result of your acne, please tell us which health professional you saw and how many times. Please enter '0' if you did not visit the health professional or in 'Other' if there were no other visits.

Please do not include visits with any professionals that took place outside of the hospital. These should be included in question 1 above. Please do not include visits made as part of this study in your answers below.

Funded by NIHR

For peer review only - http://bmjopen.bmj.com/site/about/guidelines.xhtml

For peer review only

SAFA Spironolactone for Adult Female Acne

UNIVERSITY OF Southampton

Participant's initials: | | | |

Participant's study identifier: | | | | | | | | |

Health professional you saw<br>*(If unknown, please write the department in which you saw them)*	Number of outpatient visits
Example: Dermatology nurse	*2* visits
Dermatologist	visits
Dermatology nurse	visits
Other, please specify:	visits
Other, please specify:	visits
Other, please specify:	visits

3b. Did you attend Accident and Emergency Services in the **last 6 weeks** because of your acne or side effects from treatment for your acne?

☐ Yes ☐ No, if 'No' please go to question 3c

If 'Yes', how many visits in the last 6 weeks: ______________________________

3c. In **the last 6 weeks**, have you been admitted to hospital as an inpatient as a result of your acne or side effects from treatment for your acne?

☐ Yes ☐ No, if 'No' please go to question 4

If 'Yes', for **each inpatient** visit you have had, please tell us the type of ward you were admitted to and for how many nights.

Please include any day case procedures

Visit number	The type of department or ward or reason for admission	Duration of each stay (number of nights)
Example	*Dermatology*	*2* nights

For peer review only - http://bmjopen.bmj.com/site/about/guidelines.xhtml

Funded by NIHR

SAFA
Spironolactone for Adult Female Acne

University of Southampton

Participant's initials:				Participant's study identifier:								

1		nights
2		nights

Question 4: Other services

4. In the **last 6 weeks** have you received any other publically provided services because of your acne?

☐ Yes ☐ No, if 'No' please go to question 5a

If 'Yes', please give details including type and how many times received:

Details of service	Type of service	Number of times received

Costs incurred by yourself or family

These next few questions are about the costs incurred by you and your family/friends because of your acne.

Question 5: Personal Costs

5a. In the **last 6 weeks** have you or your family/friends incurred any other costs because of your acne? Please do not include visits made as part of this study in your answers below.

☐ Yes ☐ No, if 'No' please go to question 5b

If 'Yes', please give the details below and the approximate cost of items purchased as a result of your acne.

Item	Number of items or visits	Overall cost
Example: Homeopath	*2 visits*	*£80 (2 x £40)*
Complementary therapists		
Non-prescribed medication		
Travel costs to health care appointments		

For peer review only - http://bmjopen.bmj.com/site/about/guidelines.xhtml

Funded by NIHR

For peer review only

SAFA
Spironolactone for Adult Female Acne

UNIVERSITY OF Southampton

Participant's initials: | | | |

Participant's study identifier: | | | | | | | | |

Parking costs at health care appointments		
Cosmetic and skin care products		
Other, please specify:		
Other, please specify:		
Other, please specify:		

5b. What is your current **primary occupation**? Please tick one:

☐ Paid employment ☐ self-employment ☐ Voluntary work ☐ Education/studying

☐ None of the above (i.e. retired, unemployed)

In the **last 6 weeks** has your acne had an impact on your primary occupation?

☐ Yes ☐ No, if 'No' please go to question 5c.

If 'Yes', please fill in each row of the table below **about how your acne has affected your primary occupation** in the last 6 weeks. This asks only about your acne, so if, for example, you reduced your hours worked to look after a dependent please do not put this in this table. Please do not include visits made as part of this study in your answers below.

I have had to take leave	☐ No	☐ Yes	If yes, how much leave have you taken in the last 6 weeks?<br>_____weeks ________days _______hours<br>If in paid employment or self-employment, was this paid leave?<br>☐ Yes ☐ No ☐ Mixture of paid and unpaid<br>If a mixture of paid and unpaid leave, how much of the leave was paid leave?<br>_____weeks ________days _______hours
I have reduced the hours I undertake my primary occupation each week	☐ No	☐ Yes	If yes, how many hours per week did you used to undertake?

Funded by NIHR

For peer review only - http://bmjopen.bmj.com/site/about/guidelines.xhtml

Participant's initials: | | | |

Participant's study identifier: | | | | | | | | |

			How many hours per week do you undertake now?<br><br>How long ago did this change:<br>_____weeks ________days _______hours
I have increased the hours I undertake my primary occupation each week	☐ No	☐ Yes	If yes, how many hours per week did you used to undertake?<br><br>How many hours per week do you undertake now?<br><br>How long ago did this change:<br>_____weeks ________days _______hours
I have completely stopped my primary occupation and will not be going back to it	☐ No	☐ Yes	How long ago did this change:<br>_____weeks ________days _______hours
I have changed my role within my primary occupation	☐ No	☐ Yes	If yes, what was your old role title:<br><br>What is your new role title:<br><br>How long ago did this change:<br>_____weeks ________days _______hours

5c. Have you had a family member or friend who has had to take time off paid work to accompany you to health care appointments related to your acne?

☐ Yes ☐ No, if 'No' please go to question 5d

If yes, how much leave have they had to take in the last 6 weeks to accompany you to appointments related to your acne?

________hours

For peer review only - http://bmjopen.bmj.com/site/about/guidelines.xhtml

Funded by NIHR

Participant's initials: ☐☐☐ Participant's study identifier: ☐☐☐☐☐☐☐☐

5d. **Support outside of official services** (For example, charity support groups such as The Acne and Rosacea Association, helplines etc)

In the **last 6 weeks**, have you received support or attended support groups?

☐ Yes ☐ No

If 'Yes', please list what support you have accessed and state whether you incurred any costs as a result (e.g. membership fee, participation fee, telephone cost etc)

**Type of Support**	**Cost Incurred (£)**
	£
	£
	£

Thank you for completing this questionnaire.

For peer review only

For peer review only - http://bmjopen.bmj.com/site/about/guidelines.xhtml
Funded by NIHR

SUPPORTING INFORMATION

APPENDIX S2: FURTHER SENSITIVITY AND SUBGROUP ANALYSES

The following information is presented in addition to the main paper, "Cost-effectiveness of Spironolactone for Adult Female Acne (SAFA): Economic evaluation alongside a randomised controlled trial", published in XXXX. In addition to the sensitivity analyses presented in the main paper, a further two sensitivity analyses and a sub-group analysis were agreed before analysis and conducted to explore key uncertainties around the parameters of the economic evaluation. The details of these are outlined below.

Baseline Resource use
Table S1 presents the levels of resource use, at baseline, prior to randomisation (Table S1).

Sensitivity analysis: wider resource use
In addition to the resource use data outlined in the main paper and to enable further sensitivity analysis exploring a wider perspective than that limited to the NHS, wider resource use related to acne, were collected via the participant questionnaires at all time points. These included out-of-pocket expenses (including, complementary therapist visits, cosmetic skin care products, non-NHS-prescribed medication, parking and travel costs for healthcare appointments and other) and productivity losses (including lost patient and carer productivity). These were valued using participant self-reported values and unit costs identified from published sources and reported in Table 1.

The mean (sd) cost per participant per intervention group was estimated for the 24-week treatment period for personal out-of-pocket expenses and productivity costs and summed along with NHS costs to estimate the mean difference (95% CI) in total costs (wider perspective) (Table S2). The cost utility analysis was repeated as described in the main paper with this exception.

Taking a wider cost perspective including participants' and family/friend's out of pocket costs, productivity costs and non-official services costs, found spironolactone to be dominated by placebo in the complete case analysis, that is both more costly and less effective but this is likely to reflect the amount of missing data. With multiple imputation, the ICER was estimated as £30,249 per QALY, with a 50% probability of spironolactone being cost-effective at a £30,000 per QALY (Table S3). The majority of women (97%) reported no impact on their employment as a result of their acne and thus it is mainly out-of-pocket expenses driving change from the base-case.

Sensitivity analysis: costing the intervention as per the SAFA trial protocol
The impact of costing the intervention as per the SAFA trial protocol (i.e. intervention was accessed via secondary care, excluding any research related costs) was explored in a 'secondary care delivery' sensitivity analysis. The cost utility analysis was repeated but with the intervention costed as described in Figure S1, while the placebo group was costed as in the base-case analysis, i.e. assumed no intervention costs.

Costing the intervention as it was delivered in the trial, via secondary care (see **Error! Reference source not found.**) instead of primary care (the expected standard of care if delivered in practice), the mean cost of the spironolactone intervention was £264.94 (SD £36.14). The ICER was estimated

as £141,955 per QALY, with a 12% probability of spironolactone being cost-effective at a £30,000 threshold (Table S3).

Subgroup analysis by age

A single sub-group analysis was undertaken for age (categorised as below 25 years and 25 years and over) as the clinical analysis found age significantly interacts with the outcome.[1]

The ICER was £263,871 per QALY for women under 25 years compared to £19,994 for women over 25 years of age (see Table S3). This result suggests that spironolactone is likely to be cost effective for women aged over 25 years. Whilst this finding is in line with the clinical findings, it ought to be interpreted with caution given the small sample sizes necessitated by splitting the dataset into subgroups combined with missing data.

Interpretation of sensitivity and subgroup analyses

The results of these sensitivity analyses reinforce the conclusion given in the main paper, which stated that whether 'spironolactone is considered a cost-effective treatment for persistent acne in women, depends upon the assumptions and methods utilised'. As would be expected, when delivery of the intervention was assumed to take place in secondary care (as delivered in the trial protocol) spironolactone did not appear cost-effective. This is not, however, a realistic reflection of what is likely to occur in practice. When a wider perspective was taken (instead of an NHS perspective) spironolactone was again estimated not to be cost-effective. However, there was a large proportion of missing data and when this was considered the probability of the intervention being cost-effective increased, but the ICER was not quite below the £30,000 willingness to pay threshold. With regards to the sub-group analysis by age, further research to understand if and why there may be a difference in (cost) effectiveness of spironolactone by age is warranted before drawing any conclusions.

Costs and outcomes over 52 weeks

Data was also collected beyond the treatment period (24 weeks) for up to 52 weeks. Response rates were, however, significantly lower at this time point, with 58% of participants missing EQ-5D data and 93% missing resource use data (see Supplementary Table 4). It is difficult to draw conclusions from these data, but incremental QALYs over 52 weeks was 0.0644 (95%CI 0.0093 to 0.1194) and incremental cost (NHS perspective) (see Supplementary Table 5) over the same period was £95.44 (95% CI 8.29 to 182.70).

Reference:

[1] Santer M, Lawrence M, Renz S, et al. Effectiveness of spironolactone for women with acne vulgaris (SAFA) in England and Wales: pragmatic, multicentre, phase 3, double blind, randomised controlled trial. *BMJ 2023;381:e074349*

For peer review only

SUPPLEMENTARY FIGURES

Supplementary Figure S1 | Intervention resource use as delivered via secondary care per trial protocol

For peer review only

SUPPLEMENTARY TABLES

Supplementary Table S1 | Estimates of mean baseline resource use by treatment group (available case data)

Resource	Spironolactone (N=201)		No active systemic treatment (N=209)		Mean difference
	Mean (n)	Std dev	Mean (n)	Std dev	(95% CI)
Total community-based HCP visits	0.27 (200)	0.616	0.225 (209)	0.590	0.045 (-0.072 to 0.162)
Total hospital contacts	0.119 (193)	0.446	0.095 (200)	0.396	0.024 (-0.059 to 0.108)
All medications – quantity (number)	11.711 (201)	46.065	7.903 (206)	21.570	3.809 (-3.174 to 10.791)
Total out-of-pocket items	2.027 (188)	2.735	1.939 (196)	2.438	0.088 (-0.432 to 0.607)
Lost patient work time (number reporting)	0.020 (197)	0.141	0.034 (205)	0.182	-0.014 (-0.046 to 0.018)
Lost carer work time (number reporting)	0.015 (194)	0.124	0.030 (203)	0.170	-0.014 (-0.044 to 0.015)

For peer review only

Supplementary Table S2 | Estimates of mean (sd) cost and mean (95% CI) difference in cost (UK£ 2021/22) including wider costs, by treatment group

**Resource**	**Spironolactone (N=201)**		**No active systemic treatment (N=209)**		**Mean difference**
	**Mean (n)**	**SD**	**Mean (n)**	**SD**	**(95% CI)**
Resource use costs over 24-week period:					
All intervention costs	122.87 (201)	13.04	0 (209)	0	122.87 (121.09 to 124.64)
All community-based HCP costs	6.28 (150)	24.83	3.75 (124)	16.46	2.53 (-2.60 to 7.66)
All hospital contact costs	7.28 (132)	36.42	5.73 (115)	28.09	1.55 (-6.70 to 9.79)
All prescription medication costs	4.37 (147)	11.77	5.91 (124)	18.93	-1.54 (-5.25 to 2.17)
**Total costs (NHS perspective)**	**141.99 (128)**	**57.90**	**15.64 (110)**	**45.62**	**126.35 (112.88 to 139.82)**
All personal out-of-pocket costs	69.41 (139)	113.05	82.57 (120)	148.60	-13.15 (-45.23 to 18.92)
Lost patient and carer productivity	27.87 (177)	354.76	15.95 (179)	183.54	11.93 (-46.86 to 70.71)
**Total costs (wider perspective)**	**252.67 (113)**	**490.19**	**93.53 (100)**	**144.02**	**159.14 (58.86 to 259.41)**

For peer review only

Supplementary Table S3 | Cost utility analyses and cost-effectiveness analyses results, for additional sensitivity analyses and sub-group analysis

CUA Analysis (N s, N p)	Incremental cost (95% CI)	Incremental QALYs (95% CI)	ICER	CEAC at £20,000 (£30,000) threshold*
SA: Secondary care delivery, CCA, adjusted (118,101)	265.67 (250.52 to 280.82)	0.0019 (-0.0096 to 0.0133)	£141,955	3% (12%)
SA: Wider perspective, CCA, adjusted (97,85)	102.07 (64.21 to 139.92)	-0.0027 (-0.0139 to 0.0085)	Dominated	9% (15%)
SA: Wider perspective, MI, adjusted (201,209)	133.25 (72.52 to 193.93)	0.0044 (-0.0041 to 0.0129)	£30,249	31% (50%)
Sub-group analysis: <25 years, CCA, adjusted: (28,29)	108.23 (89.09 to 127.37)	0.0004 (-0.0141 to 0.0150)	£263,871	25% (33%)
Sub-group analysis: ≥25 years, CCA, adjusted: (90,72)	133.06 (114.97 to 151.16)	0.0067 (-0.0079 to 0.0213)	£19,994	50% (62%)

95% CI=95% confidence interval; ICER =incremental cost-effectiveness ratio; N s / N p =Number randomised to spironolactone / Placebo who were included in the analysis; CCA = complete case analysis; SA refers to the different sensitivity analyses described in the Methods; QALY=Quality Adjusted Life Years; *probability of being cost-effective at a the threshold (λ) of £20,000 and £30,000 per QALY. Adjusted analyses, adjusted for stratification variables (centre, baseline severity [IGA<3 vs. ≥3]) and baseline variables (Acne QoL symptom subscale score, use of topical treatments, utility score based on EQ-5D, total costs)

For peer review only

Supplementary Table S4 | 1Proportion of Missing values (%) for key variables

Variable	Spironolactone	No active systemic treatment	Total
**Baseline variables**			
Treatment allocation	0	0	0
Centre	0	0	0
Baseline severity (IGA)	0	0	0
Acne-QoL symptom subscale score at baseline	0	0	0
Use of topical treatments (y/n)	1.00	0.48	0.73
EQ-5D at baseline	0.50	0.00	0.24
Costs at baseline	4.48	5.74	5.12
**Cost variables***			
Costs at 6 weeks	17.91	18.18	18.05
Costs at 12 weeks	14.43	23.44	19.02
Costs at 24 weeks	23.88	38.76	31.46
Costs at 52 weeks	92.54	94.26	93.41
**Outcome variables for health-related quality of life**			
EQ-5D at 6 weeks	12.44	14.35	13.41
EQ-5D at 12 weeks	13.43	20.57	17.07
EQ-5D at 24 weeks	20.40	33.49	27.07
EQ-5D at 52 weeks	54.73	61.72	58.29
**Outcome variables for Acne-related quality of life**			
Acne-QoL at 6 weeks	12.44	14.35	13.41
Acne-QoL at 12 weeks	12.44	20.57	16.59
Acne-QoL at 24 weeks	18.91	34.93	27.07
Acne-QoL at 52 weeks	52.74	61.24	57.07
**Outcomes for cost-utility and cost-effectiveness analyses***			
Total costs (treatment period)	36.32	47.38	41.95
Total QALYS (treatment period)	20.90	33.49	27.32
Change Acne-QoL symptoms (treatment period)	18.91	34.93	27.07

Treatment period = baseline to 24 weeks

*For base-case, i.e. NHS-related costs only

For peer review only

Supplementary Table S5 | Mean (Standard Deviation) Cost and Cost Difference (95% Confidence Interval) Per Patient up to 25-–52 weeks for the Intervention arm compared to usual care arm (in 2021 UK pounds sterling)

Resource	Spironolactone (N=201)		No active systemic treatment (N=209)		Mean difference
	Mean (n)	Std dev	Mean (n)	Std dev	(95% CI)
**Costs**					
All community-based HCP costs	19.64 (16)	33.25	33.24 (13)	42.00	-13.60 (-42.25 to 15.05)
Total hospital contacts cost	17.10 (15)	45.13	9.87 (13)	35.57	7.23 (-24.70 to 39.17)
All medication cost	4.81 (16)	11.23	9.66 (13)	19.41	-4.85 (-16.65 to 6.96)
Total costs (NHS perspective), 25–52 weeks	39.89 (15)	67.47	54.41 (12)	79.00	-14.52 (-72.57 to 43.52)
Total costs (NHS perspective), 0–52 weeks	179.21 (13)	76.99	83.76 (10)	123.54	95.44 (8.29 to 182.60)
**Outcomes**					
52 weeks EQ-5D-5L utility	0.9208 (92)	0.1516	0.8291 (79)	0.2664	0.0918 (0.0274 to 0.1561)
QALYs at 52 weeks	0.9158 (88)	0.1364	0.8515 (74)	0.2154	0.0644 (0.0093 to 0.1194)
52 weeks symptom Acne-QoL	21.634 (95)	6.257	19.963 (81)	5.697	1.671 (-0.122 to 3.464)
Symptom Acne QoL change at 52 weeks compared to baseline	8.613 (95)	7.154	6.951 (81)	6.500	1.663 (-0.385 to 3.710)

For peer review only

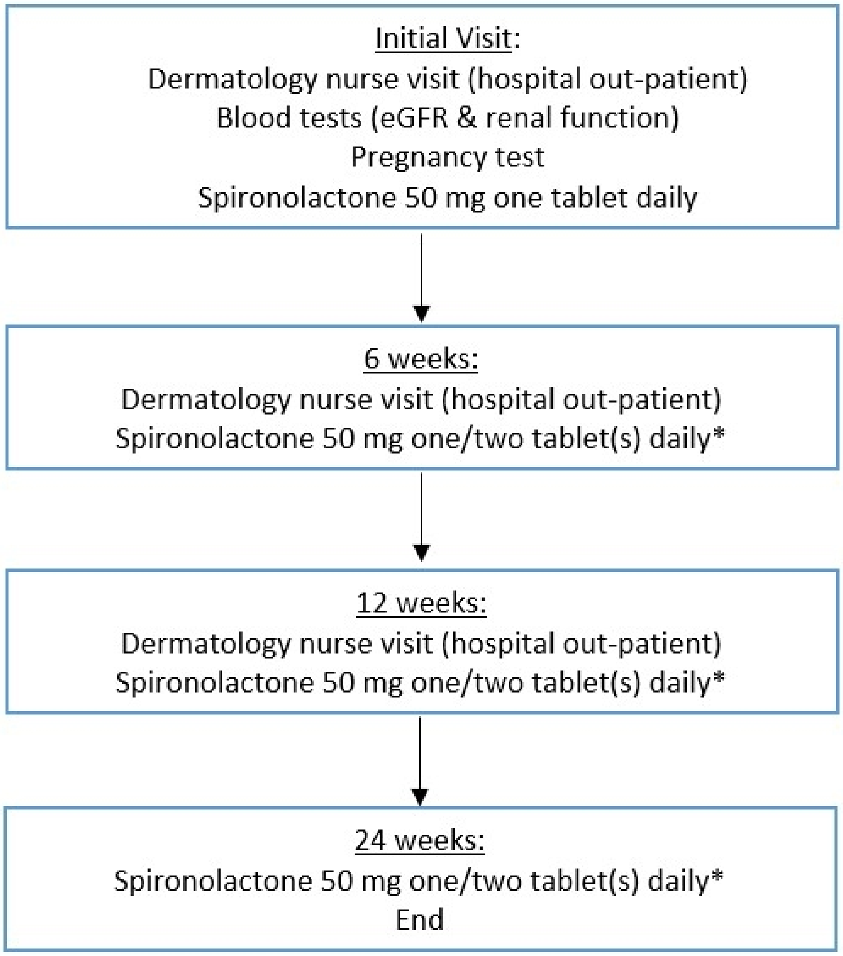

*At 6 weeks, dose was increased to 100 mg/day, assuming treatment was tolerated, which was the case for 182/184 (99%) of available patients in the spironolactone arm of the study

152x187mm (96 x 96 DPI)

CHEERS 2022 Checklist – For SAFA Trial

	Item	Guidance for Reporting	Reported in section
**TITLE**			
Title	1	Identify the study as an economic evaluation and specify the interventions being compared.	Title and abstract
**ABSTRACT**			
Abstract	2	Provide a structured summary that highlights context, key methods, results and alternative analyses.	Abstract
**INTRODUCTION**			
Background and objectives	3	Give the context for the study, the study question and its practical relevance for decision making in policy or practice.	Introduction
**METHODS**			
Health economic analysis plan	4	Indicate whether a health economic analysis plan was developed and where available.	Methods
Study population	5	Describe characteristics of the study population (such as age range, demographics, socioeconomic, or clinical characteristics).	Results - Participant characteristics
Setting and location	6	Provide relevant contextual information that may influence findings.	Abstract, methods and results
Comparators	7	Describe the interventions or strategies being compared and why chosen.	Methods - Intervention and comparator. Sensitivity analysis 2 and 3.
Perspective	8	State the perspective(s) adopted by the study and why chosen.	Abstract and Methods
Time horizon	9	State the time horizon for the study and why appropriate.	Abstract and methods
Discount rate	10	Report the discount rate(s) and reason chosen.	Methods – Incremental analysis
Selection of outcomes	11	Describe what outcomes were used as the measure(s) of benefit(s) and harm(s).	Methods – measuring outcomes
Measurement of outcomes	12	Describe how outcomes used to capture benefit(s) and harm(s) were measured.	Methods – measuring outcomes
Valuation of outcomes	13	Describe the population and methods used to measure and value outcomes.	Methods – measuring outcomes
Measurement and valuation of resources and costs	14	Describe how costs were valued.	Methods – Measuring costs
Currency, price date, and conversion	15	Report the dates of the estimated resource quantities and unit costs, plus the currency and year of conversion.	Methods – Measuring costs
Rationale and description of model	16	If modelling is used, describe in detail and why used. Report if the model is publicly available and where it can be accessed.	N/A
Analytics and assumptions	17	Describe any methods for analysing or statistically transforming data, any extrapolation methods, and approaches for validating any model used.	Methods – Incremental analysis
Characterizing heterogeneity	18	Describe any methods used for estimating how the results of the study vary for sub-groups.	Supplementary material – Sub-group
Characterizing distributional effects	19	Describe how impacts are distributed across different individuals or adjustments made to reflect priority populations.	Not reported – this is a new item of the checklist which was not published at the time the study was designed.
Characterizing uncertainty	20	Describe methods to characterize any sources of uncertainty in the analysis.	Methods and supplementary analyses – sensitivity analyses
Approach to engagement with patients and others affected by the study	21	Describe any approaches to engage patients or service recipients, the general public, communities, or stakeholders (e.g., clinicians or payers) in the design of the study.	Methods - Patient and public involvement (PPI) and measuring costs
**RESULTS**			
Study parameters	22	Report all analytic inputs (e.g., values, ranges, references) including uncertainty or distributional assumptions.	Results
Summary of main results	23	Report the mean values for the main categories of costs and outcomes of interest and summarise them in the most appropriate overall measure.	Results
Effect of uncertainty	24	Describe how uncertainty about analytic judgments, inputs, or projections affect findings. Report the effect of choice of discount rate and time horizon, if applicable.	Results
Effect of engagement with patients and others affected by the study	25	Report on any difference patient/service recipient, general public, community, or stakeholder involvement made to the approach or findings of the study	Methods - Patient and public involvement (PPI) and measuring costs
**DISCUSSION**			

For peer review only

Study findings, limitations, generalizability, and current knowledge	26	Report key findings, limitations, ethical or equity considerations not captured, and how these could impact patients, policy, or practice.	Discussion
**OTHER RELEVANT INFORMATION**			
Source of funding	27	Describe how the study was funded and any role of the funder in the identification, design, conduct, and reporting of the analysis	Funding sources
Conflicts of interest	28	Report authors conflicts of interest according to journal or International Committee of Medical Journal Editors requirements.	Conflicts of interest

Husereau D, Drummond M, Augustovski F, de Bekker-Grob E, Briggs AH, Carswell C, Caulley L, Chaiyakunapruk N, Greenberg D, Loder E, Mauskopf J, Mullins CD, Petrou S, Pwu RF, Staniszewska S; CHEERS 2022 ISPOR Good Research Practices Task Force. Consolidated Health Economic Evaluation Reporting Standards 2022 (CHEERS 2022) Statement: Updated Reporting Guidance for Health Economic Evaluations. BMJ. 2022;376:e067975.

The checklist is Open Access distributed in accordance with the terms of the Creative Commons Attribution (CC BY 4.0) license, which permits others to distribute, remix, adapt and build upon this work, for commercial use, provided the original work is properly cited. See: http://creativecommons.org/licenses/by/4.0/.

For peer review only

BMJ Open

Cost-effectiveness of Spironolactone for Adult Female Acne (SAFA): Economic evaluation alongside a randomised controlled trial

Journal:	*BMJ Open*
Manuscript ID	bmjopen-2023-073245.R2
Article Type:	Original research
Date Submitted by the Author:	07-Aug-2023
Complete List of Authors:	Pyne, Sarah; University of East Anglia, Health Economics Group, Norwich Medical School<br>Sach, Tracey; University of East Anglia, Health Economics Group, Norwich Medical School; University of Southampton, School of Primary Care, Population Sciences and Medical Education<br>Lawrence, Megan; University of Southampton<br>Renz, Susanne; University Hospital Southampton NHS Foundation Trust<br>Eminton, Zina; University Hospital Southampton NHS Foundation Trust<br>Stuart, Beth; Queen Mary University of London, Centre for Evaluation and Methods Wolfson Institute of Population Health<br>Thomas, Kim; University of Nottingham, Dermatology<br>Francis, Nick; University of Southampton, Primary Care Research Centre<br>Soulsby, Irene; University of Southampton Faculty of Medicine, Primary Care and Population Sciences<br>Thomas, Karen ; University of Southampton, Public contributor, Primary Care Research Centre<br>Permyakova, Natalia; University of Southampton; University Hospital Southampton NHS Foundation Trust<br>Ridd, Matthew; University of Bristol, Population Health Sciences<br>Little, Paul; University of Southampton<br>Muller, Ingrid; University of Southampton<br>Nuttall, Jacqui; University of Southampton<br>Griffiths, Gareth; University of Southampton<br>Layton, Alison M; University of York, Skin Research Centre, Hull York Medical School<br>Santer, Miriam; University of Southampton, Primary Medical Care
**Primary Subject Heading**:	Health economics
Secondary Subject Heading:	Dermatology
Keywords:	Clinical Trial, Dermatology < INTERNAL MEDICINE, Health economics < HEALTH SERVICES ADMINISTRATION & MANAGEMENT, HEALTH ECONOMICS, Acne < DERMATOLOGY, Adult dermatology < DERMATOLOGY

SCHOLARONE™
Manuscripts

I, the Submitting Author has the right to grant and does grant on behalf of all authors of the Work (as defined in the below author licence), an exclusive licence and/or a non-exclusive licence for contributions from authors who are: i) UK Crown employees; ii) where BMJ has agreed a CC-BY licence shall apply, and/or iii) in accordance with the terms applicable for US Federal Government officers or employees acting as part of their official duties; on a worldwide, perpetual, irrevocable, royalty-free basis to BMJ Publishing Group Ltd ("BMJ") its licensees and where the relevant Journal is co-owned by BMJ to the co-owners of the Journal, to publish the Work in this journal and any other BMJ products and to exploit all rights, as set out in our licence.

The Submitting Author accepts and understands that any supply made under these terms is made by BMJ to the Submitting Author unless you are acting as an employee on behalf of your employer or a postgraduate student of an affiliated institution which is paying any applicable article publishing charge ("APC") for Open Access articles. Where the Submitting Author wishes to make the Work available on an Open Access basis (and intends to pay the relevant APC), the terms of reuse of such Open Access shall be governed by a Creative Commons licence – details of these licences and which Creative Commons licence will apply to this Work are set out in our licence referred to above.

Other than as permitted in any relevant BMJ Author's Self Archiving Policies, I confirm this Work has not been accepted for publication elsewhere, is not being considered for publication elsewhere and does not duplicate material already published. I confirm all authors consent to publication of this Work and authorise the granting of this licence.

For peer review only

TITLE:

Cost-effectiveness of Spironolactone for Adult Female Acne (SAFA): Economic evaluation alongside a randomised controlled trial

manuscript word count: 4,205/4,000 (recommended) Words
Table count: 3
Figure count: 2

AUTHORS:

Sarah Pyne,[1] Tracey H Sach*,[1,2] Megan Lawrence,[3] Susanne Renz,[3] Zina Eminton,[3] Beth Stuart,[2,4] Kim S Thomas,[5] Nick Francis,[2] Irene Soulsby,[6] Karen Thomas,[6] Natalia Permyakova,[3] Matthew J Ridd,[7] Paul Little,[4] Ingrid Muller,[2] Jacqueline Nuttall,[3] Gareth Griffiths,[3] Alison Layton,[8] Miriam Santer,[2]

[1] Health Economics Group, Norwich Medical School, University of East Anglia, Norwich, UK

[2] Primary Care Research Centre, School of Primary Care, Population Sciences and Medical Education, Faculty of Medicine, University of Southampton, Southampton, UK

[3] Southampton Clinical Trials Unit, University of Southampton and University Hospital Southampton NHS Foundation Trust, Southampton, UK

[4] Centre for Evaluation and Methods, Wolfson Institute of Population Health, Faculty of Medicine and Dentistry, Queen Mary University of London, London, UK

[5] Centre of Evidence Based Dermatology, School of Medicine, University of Nottingham, Nottingham, UK

[6] Public contributor, UK

[7] Population Health Sciences, Bristol Medical School, University of Bristol, Bristol, UK

[8] Skin Research Centre, Hull York Medical School, University of York, York, UK

***Corresponding author:** Professor Tracey Sach, Professor in Health Economics, School of Primary Care, Population Sciences and Medical Education, Faculty of Medicine, University of Southampton, Southampton, UK. Email: T.sach@Soton.ac.uk

For peer review only

ABSTRACT (299/300 WORDS)

Objective

This study aims to estimate the cost-effectiveness of oral spironolactone plus routine topical treatment compared with routine topical treatment alone for persistent acne in adult women from a British NHS perspective over 24-weeks.

Design

Economic evaluation undertaken alongside a pragmatic, parallel, double-blind, randomised trial.

Setting

Primary and secondary healthcare, community and social media advertising.

Participants

Women ≥18 years with persistent facial acne judged to warrant oral antibiotic treatment.

Interventions

Participants were randomised 1:1 to 50 mg/day spironolactone (increasing to 100mg/day after 6 weeks) or matched placebo until week-24. Participants in both groups could continue topical treatment.

Main outcome measures

Cost-utility analysis assessed incremental cost per Quality-Adjusted Life Year (QALY) using the EQ-5D-5L. Cost-effectiveness analysis estimated incremental cost per unit change on the Acne-QoL symptom subscale. Adjusted analysis included randomisation stratification variables (centre, baseline severity [IGA <3 versus ≥3]), and baseline variables (Acne-QoL symptom subscale score, resource use costs, EQ-5D score and use of topical treatments).

Results

Spironolactone did not appear cost-effective in the complete case analysis (n=126 spironolactone, n=109 control), compared with no active systemic treatment (adjusted incremental cost per QALY £67,191; unadjusted £34,770). Incremental cost per QALY was £27,879 (adjusted), just below the upper National Institute for Health and Care Excellence's (NICE) threshold value of £30,000, where multiple imputation took account of missing data. Incremental cost per QALY for other sensitivity analyses varied around the base-case, highlighting the degree of uncertainty. The adjusted incremental cost per point change on the Acne-QoL symptom subscale for spironolactone compared with no active systemic treatment was £38.21 (complete case analysis).

Conclusions

The results demonstrate a high level of uncertainty, particularly with respect to estimates of incremental QALYs. Compared with no active systemic treatment, spironolactone was estimated to be marginally cost-effective where multiple imputation was performed but was not cost-effective in complete case analysis.

For peer review only - http://bmjopen.bmj.com/site/about/guidelines.xhtml

STRENGTHS AND LIMITATIONS

- Our study is based on individual patient level data collected alongside the first large pragmatic, parallel, double-blind, randomised trial of spironolactone for acne.
- In addition to the base-case analysis seeking to answer the question of whether spironolactone is cost-effective compared with no active systemic treatment (both groups could use routine topical treatments) in women with persistent acne, a number of sensitivity analyses were undertaken to provide a range on estimates of cost-effectiveness under different scenarios.
- Differential rates of missing data between groups over time were addressed by undertaking both a complete case analysis and multiple imputation to explore the impact of missing data on the study conclusions.
- As the study was constrained by the design of the clinical trial, the base-case did not reflect real-world prescribing in the comparator group, limiting interpretation of the results.
- The results reflect the method of data collection and may have been limited as a consequence of resource-use under-reporting, short time-frame and limited sensitivity of the EQ-5D outcome measure in patients with acne.

INTRODUCTION

Acne (acne vulgaris) is a common condition, affecting >80% of people at some point in their life.[1] Its impact on the NHS is considerable, being responsible for around 3.5 million consultations with a GP[1] and 70,000 referrals for specialist care[2] in the UK annually. As well as direct burdens to the NHS, adults (18–30 years) with severe acne in the UK have higher unemployment rates[3] and a small study by Jowett and Ryan (1985)[4] showed that 45% (13/29) of acne patients reported interpersonal difficulties at work.

There are many treatment options for women with moderate-to-severe acne, but a recent network meta-analysis (NMA) demonstrated paucity of good quality evidence and the complexity of choice.[5] Informed in large part by this NMA and the associated economic model,[6] the National Institute of Health and Care Excellence (NICE) guidelines on the management of acne vulgaris recommend a fixed combination topical preparation containing retinoids, benzoyl peroxide or antibiotics as first-line treatment for any severity of acne, whilst a fixed combination topical agent plus oral lymecycline or doxycycline once daily is recommended for moderate-to-severe acne. The latter is also recommended for moderate-to-severe acne that does not respond adequately to a 12-week course of treatment that does not include an oral antibiotic.[7] The guidance states that treatment options including an antibiotic (topical or oral) should only be continued for more than 6 months in exceptional circumstances (other guidelines limit oral antibiotic duration to 3 months)[8–10] and that clinicians should be aware of the associated risks of antimicrobial resistance. Doctors, however, report many challenges when trying to discontinue oral antibiotics.[11]

Spironolactone is already used off license for women with acne, is an inexpensive treatment choice and could play a role in reducing antibiotic use.[12] Literature searches did not, however, find any previously published economic evaluations on the cost-effectiveness of spironolactone in this group of patients, although there are two other ongoing studies of spironolactone in France and the USA,

For peer review only - http://bmjopen.bmj.com/site/about/guidelines.xhtml

the former of which includes an economic evaluation.[13,14] In this paper we estimate the cost-effectiveness of spironolactone plus routine topical treatment compared with no active systemic treatment plus routine topical treatment for persistent acne in adult women from a British NHS perspective over 24-weeks.

PATIENTS AND METHODS

The Spironolactone for Adult Female Acne (SAFA) trial was a pragmatic, multicentre, participant-led, and clinician-blind, superiority, randomised trial with two parallel treatment groups: spironolactone compared to placebo in women aged 18 years and older with facial acne judged to warrant oral antibiotics. The economic evaluation was nested within this trial.

Participants were recruited in primary care, secondary care and through advertising (community and social media). Baseline assessment was conducted by a research nurse and/or dermatologist in secondary care clinics to ensure standard clinical assessments, as the Investigator's Global Assessment (IGA) for acne was an inclusion criterion and an important secondary outcome. Baseline appointments included a pregnancy test, blood test (to exclude renal impairment or raised serum potassium), participant photo to aid recall about changes in acne and contraceptive counselling. The first participant was recruited in June 2019 and the last in August 2021, whilst follow-up finished February 2022. The SAFA trial is described in more detail in the clinical paper.[15,16]

Participants were randomised 1:1 using online software to either 50 mg/day spironolactone or matched placebo until week-6, increasing to 100 mg/day spironolactone or matched placebo until week-24, assuming treatment was tolerated. Participants were stratified by recruitment centre and baseline acne severity (IGA<3 vs IGA≥ 3). In both groups participants could continue using topical treatment. Between baseline and week-12 participants were asked not to take oral treatment for acne other than study medication, except for oral contraception taken for over 3 months previously. After 12 weeks, participants in both groups could receive usual care, including oral treatments, such as oral antibiotics, hormonal treatment or isotretinoin. In both groups participants were followed up face-to-face (or by video call or telephone due to COVID-19) at week-6 and week-12 in secondary care, with primary outcome assessment at week-12, and longer-term follow-up by questionnaires at week-24.

Although in the clinical trial, spironolactone plus routine topical treatment was compared to placebo plus routine topical treatment, it is most appropriate in economic evaluations to compare an active treatment to current usual care.[17] Therefore, to utilise the data collected in the trial whilst reflecting a useful analysis to decision makers in practice, this economic evaluation compared spironolactone plus routine topical treatment to not active systemic treatment plus routine topical treatment.

Measuring costs

In keeping with an NHS perspective, all acne-related resource use data, including intervention, primary and secondary care visits, and prescription medication use, were collected for participants in both groups. Personal Social Services (PSS) resource use was not collected, as patient and clinician contributors did not anticipate these being incurred by participants.

For peer review only - http://bmjopen.bmj.com/site/about/guidelines.xhtml

Resource use data was collected via case report forms and participant questionnaires (see supplementary material Appendix S1 for a copy), designed with the input of public contributors, at baseline (collecting the preceding 6 weeks), week-6, week-12 and week-24 for the intervention phase.

Resource uses were valued using UK unit costs (£ Sterling) for the most current price year available at the start of analysis (financial year 2021) and identified from published sources.

The intervention was costed as described in
Figure 1, which assumes that standard treatment with spironolactone, if adopted, will be delivered in primary care, including two GP visits (unless >45 years of age), baseline blood test and the cost of spironolactone (50 mg 6 weeks, 100 mg 18 weeks).[10,18–20] No intervention costs (placebo tablets, GP visits to prescribe placebo tablets or blood tests) were included for the no active systemic treatment group as these would not occur if no intervention was being given (the comparator for this economic evaluation).

Acne-related resource use data related to visits to community-based healthcare professionals (HCP), visits to hospital out-patient and in-patient services (including accident and emergency) and prescribed medication costs were self-reported via participant questionnaires at all time-points, including baseline for participants in both groups. When asked about medication use, participants were asked to report only what they had been prescribed since the previous follow-up visit. Unit costs for each visit-type were combined with this data to estimate the total community-based HCP visit costs and the total hospital contact costs. Participants were also asked for details of prescribed acne-related medication including type, strength and quantity. Unit costs for all medication types[21] were used to estimate the prescription costs over the 24-week treatment period.

The mean (sd) cost per participant per intervention group was estimated for the 24-week treatment period, for each of the cost types described above and mean difference (95% CI) in NHS cost was estimated.

Measuring outcomes
The primary economic outcome measure was QALYs over the trial period of 24 weeks, as measured by the generic preference-based EQ-5D-5L questionnaire.[22] Responses were converted to utility scores using the EQ-5D-5L Crosswalk UK preference weights, as this was in line with recommendations at the point analysis started, where utility ranges from -0.594 to 1.[23,24] Utility values were used to estimate QALYs over 24 weeks, using both linear interpolation and area under the curve analysis.[25]

A secondary economic outcome was the Acne-QoL symptom sub-scale score (five questions with seven responses to each)[26,27] at week-24, used as an estimate of effectiveness, which enables comparison with future economic studies in acne.

Economic analysis
The base-case cost-utility analysis (CUA) and secondary cost-effectiveness analysis (CEA) incorporated all randomised participants with complete cost and outcome data. Given the 24-week time-horizon, costs and benefits were not discounted.[24]

For peer review only - http://bmjopen.bmj.com/site/about/guidelines.xhtml

The base-case CUA estimated the incremental cost per QALY (incremental cost-effectiveness ratio, ICER) to enable comparison with the cost-utility of other interventions. The incremental cost (95% CI) and QALY change (95% CI) between groups was estimated unadjusted and adjusted for randomisation stratification variables (centre, baseline severity [IGA <3 versus ≥3]), and baseline variables (including Acne-QoL symptom subscale score, resource use costs, EQ-5D score and use of topical treatments (Y/N)). In line with NICE guidance,[24] we estimated whether the intervention was cost-effective by comparing the ICER with a cost-effectiveness threshold of £20,000 to £30,000 per QALY.

A CEA estimated the incremental cost per unit change on the Acne-QoL symptom sub-scale score. The incremental cost (95% CI) and Acne-QoL symptom sub-scale change (95% CI) between groups was estimated unadjusted and adjusted as described for the base-case CUA. The CUA and CEA were undertaken using a regression-based approach (seemingly unrelated regression equations).[28]

Published guidelines for the economic evaluation of health care interventions were followed as appropriate.[29,30]

To estimate the level of uncertainty associated with the decision regarding cost-effectiveness, Fieller's theorem was used to calculate[31] the probability of being cost-effective at the £20,000 and £30,000 willingness-to-pay threshold values.[24] Non-parametric bootstrapping was conducted to generate 10,000 estimates of incremental costs and benefits. From this, Cost-Effectiveness Acceptability Curves (CEACs) were generated to show the probability that the intervention is estimated to be cost-effective at different willingness-to-pay values.

Several sensitivity analyses were agreed and specified in the health economic analysis plan (HEAP) before analysis to explore key uncertainties around important parameters in the economic evaluation. The impact of missing data on cost-effectiveness estimates was explored by undertaking multiple imputation (SA1), assuming that the data was missing at random (MAR) and using chained equations to handle the missing cost and outcome data.[31] Secondly, the impact of costing the intervention as per the SAFA trial protocol (i.e. intervention was accessed via secondary care, excluding any research related costs) was explored (SA2). The cost utility analysis was repeated but with the intervention costed as described in Figure S1, while the placebo group was costed as in the base-case analysis, i.e. assumed no intervention costs. Thirdly, the CUA was repeated assuming that, as this patient population had persistent acne of sufficient severity to warrant treatment with oral antibiotics, all women in the no active systemic treatment group took oral antibiotics (lymecycline or doxycycline, 1 tablet daily for 24 weeks) as per NICE guidance[32] , in addition to topical treatment (SA3). To cost this intervention the weighted mean cost per dose of doxycycline/lymecycline was used (Table 1) and two GP visits assumed. Due to a lack of evidence about the incremental QALYs between spironolactone plus topical treatment versus oral antibiotics plus topical treatment a threshold analysis was performed to ascertain what level of incremental QALYs would switch the intervention between cost-effective and not cost-effective. Incremental costs (95% CI) and the threshold value for incremental QALYs are presented in the results. Potential costs associated with antibiotic-related side-effects and the societal costs of over prescribing of oral antibiotics were not included. Lastly, a sensitivity analysis exploring a wider perspective than that limited to the NHS was conducted (SA4). In addition to NHS-related resource use data, the following was collected via participant questionnaire: out-of-pocket expenses (including, complementary therapist visits,

For peer review only - http://bmjopen.bmj.com/site/about/guidelines.xhtml

cosmetic skin care products, non-NHS-prescribed medication, parking and travel costs for healthcare appointments and other) and productivity losses (including lost patient and carer productivity). These were valued using participant self-reported values and unit costs identified from published sources, as reported in Table 1, and summed along with NHS costs to estimate the mean difference (95% CI) in total costs (wider perspective). Utility analysis was then repeated as described for the base case. A sub-group analysis based on age was also conducted and is presented in supplementary material appendix S2.

Stata MP version 17 was used to conduct the analyses. A health economic analysis plan (HEAP) was written and followed; a copy is available from the corresponding author.

Patient and public involvement (PPI)
Key questions relating to research design were explored with a virtual acne-specific patient panel and patient survey carried out via the UK Dermatology Clinical Trials Network (UKDCTN). Two public contributors (IS and KaT) with experience of acne were members of the Trial Management group as part of this role they helped identify relevant resources and outcomes and how this data should be collected. They also contributed to the interpretation and write-up if the health economics component.

RESULTS

Participant characteristics
The clinical trial results, including details on sample size and participant characteristics, are reported elsewhere.[16] Of the 410 women recruited to the trial, 201 were randomly assigned to spironolactone and 209 allocated to placebo at the start of the trial. All were allowed to continue routine topical treatment. At week-24 126 women in the spironolactone group and 109 women in the placebo group had complete cost and outcome data, and these formed the base-case unadjusted CUA. Mean age was 29.2 years, mean BMI was 26.1, at baseline 83% (340/410) participants were using or had used topical treatments, and the majority (75% [306/410]) had acne for two or more years. There were no significant differences in characteristics between groups.[16]

Costs
The Unit costs used in the analysis are presented in Table 1. The levels of resource use in each group were very similar prior to randomisation (Table S1).

The majority of responding women in the spironolactone group (182/184, 99%) increased to two tablets of spironolactone at week 6. The 'standard treatment' approach used in the base-case economic evaluation, gave rise to a mean total intervention resource use cost of £122.87 (SD £13.04) per participant in the spironolactone group (Table 2).

Using available case data, when intervention use was combined with other health resource use, the unadjusted mean incremental cost per participant was £126.35 (95% CI, £112.88 to £139.82) for women receiving spironolactone compared to women receiving no active systemic treatment in the base-case (Table 2). Excluding intervention costs, the difference was not significant between groups. While patients were asked about in-patient visits, none were reported.

For peer review only - http://bmjopen.bmj.com/site/about/guidelines.xhtml

Outcomes

The mean (sd) QALYs over 24 weeks in the spironolactone group were 0.417 (0.058) per participant compared to 0.404 (0.079) per participant in the no active systemic treatment group, giving an incremental difference of 0.013 (95% CI -0.0024 to 0.0289) QALYs using unadjusted available case data (Table 2). The wide 95% confidence intervals around mean estimates demonstrates a high degree of uncertainty.

The mean (sd) change from baseline in Acne-QoL symptom subscale score at 24 weeks was 8.15 (6.12) in the spironolactone group compared to 4.46 (6.34) in the no active systemic treatment group. Thus, the incremental difference in score was 3.68 (95% CI 2.26 to 5.11) in favour of the spironolactone group (Table 2).

Base-case Cost Utility Analysis

In the complete case analysis, the incremental cost for the spironolactone group (n=118) compared to the no active systemic treatment group (n=101) was £125.36 (95% CI, £111.13 to £139.58) (unadjusted this was £125.53 [95% CI £112.15 to £138.91]) (Table 3). The adjusted incremental QALYs for the spironolactone group compared with the no active systemic treatment group was 0.0019 (95% -0.0096 to 0.0133) (unadjusted was 0.0036, 95% CI -0.0117 to 0.0189). The ICER was £67,191 (unadjusted £34,770) per QALY. At a willingness to pay of £30,000 per QALY there was a 35% (unadjusted 47%) chance of spironolactone being cost-effective in this population of women with persistent acne.

The cost-effectiveness acceptability curves (**Error! Reference source not found.**) of the adjusted and unadjusted base-case analysis, show that the probability of spironolactone being cost-effective only approaches 50% as the threshold value approaches £120,000 (adjusted), demonstrating a high degree of uncertainty associated with the decision under these conditions.

Secondary Cost Effectiveness analysis

The adjusted incremental difference in cost per point change on the Acne-QoL symptom subscale for the spironolactone group (n=119) compared to no active systemic treatment group (n=102) was £38.21 (unadjusted £35.91) based on a complete case analysis (Table). How much a decision maker would be willing to pay for a point change on the Acne-Qol symptom subscale is unknown.

Sensitivity analyses

The results of the sensitivity analyses are presented in Table 3 and prove influential to the conclusions reached. The ICER varies around the base-case from £27,879 (with a 53% probability of being cost-effective at £30,000 threshold for the MI analysis (SA1) to spironolactone being dominated (more costly and less effective than control) for the wider perspective (CCA) analysis.

There were differential rates of attrition with greater missing data in the no active systemic treatment group, compared to spironolactone group, by 24-weeks follow-up, for costs (39% vs. 24%, respectively) and EQ-5D-5L (33% vs. 20%, respectively). This may offer some explanation for why, when using multiple imputation in a sensitivity analysis the ICER was less than in the complete case, adjusted analysis (Table 3).

[32]With regards to the oral antibiotic control analysis (SA3), the planned threshold analysis using the complete case, adjusted data found that the incremental QALY benefit for spironolactone

For peer review only - http://bmjopen.bmj.com/site/about/guidelines.xhtml

compared with oral antibiotics would have to decrease to 0.00057 (0.000384, MI adjusted) or less to switch the ICER from being cost-effective to not cost-effective at a £30,000 threshold. The plausibility of this value is unclear but research comparing spironolactone with oral antibiotics, currently underway[13] will enable an assessment of plausibility once published.

Of note regarding the wider perspective sensitivity analysis (SA4) The majority of women (97%) reported no impact on their employment as a result of their acne and thus it is mainly out-of-pocket expenses driving change from the base-case.

The results of a subgroup analysis undertaken for women aged <25 years and ≥25 years are reported in online supplementary material appendix S2. See Table S2 for results.

DISCUSSION

This economic study finds a high degree of uncertainty about whether spironolactone is likely to be cost-effective. Our economic evaluation provides a range of estimates for the cost effectiveness of spironolactone used alongside routine topical treatment. The base-case analysis, where the comparator is no active systemic treatment plus routine topical treatment, and the delivery of the intervention is costed as via primary care, spironolactone was not estimated to be cost-effective in the unadjusted and adjusted complete case analyses. However, in the adjusted analysis using multiple imputation (MI) the ICER was estimated to be just under the £30,000 per QALY threshold. This divergence in conclusion between the complete case and MI analysis demonstrates the impact of missing data (attrition bias) and suggests more weight ought to be placed on the MI analysis.[33] The results of other sensitivity analyses (Table 3) varied around the base-case, adding to the uncertainty of the results. [13]

This economic evaluation followed a Health Economic Analysis Plan finalised before data was received for analysis reducing bias in the results from selective reporting or cherry-picked analyses.[34] Another strength of this economic evaluation is that it can provide reliable estimates of cost effectiveness based on individual participant level data, collected at little marginal cost, alongside a randomised controlled trial. This is, however, also a limitation in that within trial health economic evaluations are constrained by the question, timeframe, and data collected, particularly in placebo-controlled trials. In particular there are five main limitations to acknowledge: (1) the assumptions required to compare spironolactone to inactive systemic treatment; (2) the assumptions required to undertake a sensitivity analysis using oral antibiotics as the comparator; (3) the sensitivity and validity of the EQ-5D-5L in patients with acne; (4) the time frame of the analysis; and (5) the use of complete case analysis rather than the analysis using multiple imputation to take account of missing data as the base case analysis. We look at these in turn below, but all should be borne in mind when interpreting the results.

Firstly, ideally economic evaluations should compare an active treatment to current usual care. The funder for this trial preferred the placebo comparator to current usual care.[17] We wanted our primary analysis to reflect as closely as possible the data collected in the actual trial whilst reflecting a useful analysis to decision making in practice. We therefore felt the most appropriate comparator would be no active systemic treatment, rather than placebo, which would not reflect reality. Placebos are not used in routine practice, but some evidence of placebo effects has been documented in acne.[5] Therefore, the base-case set out to answer the question of whether

For peer review only - http://bmjopen.bmj.com/site/about/guidelines.xhtml

spironolactone is cost-effective compared with no active systemic treatment (both groups could use routine topical treatments) to align with the clinical question funded. A limitation of this is that, because it does not account for the potential impact of a placebo effect, it may result in underestimation of the QALY gain with spironolactone compared with not providing spironolactone, and hence underestimate its cost-effectiveness. We also excluded the research costs associated with administering the placebo (costs of the pills and appointments to administer them) but did including ongoing costs associated with NHS resource use related to acne in both arms of the study. There is also uncertainty about how many, if any, additional GP visits might have occurred in the usual care group if they had actually received usual care as opposed to placebo during the trial. It is not possible to know how costs and effects would differ between our placebo group and a group without any active systemic treatment because we did not have the latter group in the study. We feel the assumptions made are required to make the analysis most useful to practice but acknowledge they may mean the estimates of the cost-effectiveness of spironolactone are conservative.

Secondly, in practice clinicians are unlikely to send women away with no active treatment if they consulted with acne persisting beyond 6 months. As advised by the trial clinicians, the clinically important comparator may be another systemic treatment rather than no active systemic treatment. To address this a sensitivity analysis assuming, for cost purposes, all women in the no active systemic treatment group received an oral antibiotic (in addition to topical treatments) for 24 weeks was planned. This analysis assumed that incremental QALYs remain the same as in the base-case analysis, which we acknowledge is unlikely. There is limited economic evidence comparing oral antibiotics in combination with routine topical treatment compared with routine topical treatment alone[5]. Despite these limitations and while the results of this sensitivity analysis should be interpreted with caution, considering the assumptions made, the analysis serves to provide a lower range estimate for the cost effectiveness of spironolactone that better reflects accepted standard-of-care, based upon current NICE guidelines.[32] Further evidence, from randomised controlled trials,[13,14] is required to determine whether this is a likely scenario and to draw conclusions.

Thirdly, the uncertainty highlighted by this study may be impacted, in part, by the method of measuring utility, an area where further research would be valuable. The conclusion reached about cost-effectiveness was sensitive to the estimates of QALYs generated from EQ-5D-5L, despite 46% in the intervention group and 43% in the control group reporting perfect health (EQ-5D-5L health state 11111) at baseline. For these participants, the EQ-5D-5L had no potential to measure improvements in health-related quality of life. This likely contributes to the wide 95% confidence intervals around the incremental QALY estimates in this study, which means we cannot be certain spironolactone improves QALYs rather than have no difference or worsen QALYs. At design stage, there was discussion about the possible use of other instruments, however, the limited published evidence supported the use of the EQ-5D for acne.[35,36] Like Klassen et al[36] we find that women with persistent acne report most problems on the pain/discomfort and anxiety/depression dimensions of the EQ-5D. Further research using the EQ-5D data generated in this study alongside that elicited in other studies of acne would help inform future studies about the validity and responsiveness of this instrument for acne.

Fourthly, we acknowledge that the analysis was conducted for a 24-week timeframe and that were a longer timeframe taken the cost-effectiveness of spironolactone may improve if, for instance, there

For peer review only - http://bmjopen.bmj.com/site/about/guidelines.xhtml

is a sustained effect once treatment stops. We sought to collect resource use and utility data up to 52 weeks but due to reduced data completion at 52 weeks (see supplementary material for details) it was not feasible to analyse results to a longer time horizon.[32]

Finally, a complete case analysis was specified in the Health Economic Analysis Plan as the base case analysis (with multiple imputation as a sensitivity analysis) reflecting a desire to be consistent with the approach undertaken in the Statistical Analysis Plan for the clinical primary outcome. With the benefit of hindsight primary concern ought to have been around the level of missing economic data, which is known to often be greater than that for clinical outcomes. However, both complete case and multiple imputation analyses are reported, as planned, so that the impact of missing data on the results can be clearly seen.

Our study provides estimates of the cost-effectiveness of spironolactone in women with persistent acne using the trial data and a range of scenarios. It highlights that there is considerable uncertainty about whether spironolactone is cost-effective and the need for further research with comparators more akin to clinical practice. The complete case analysis estimated ICERs in excess of the upper NICE threshold of £30,000 per QALY but this analysis took a conservative approach since it may be that incremental QALYs for spironolactone would have been greater had we been able to control for any placebo effect and had more complete data beyond 24 weeks. When taking into account missing data the ICER was below the upper NICE threshold suggesting spironolactone may be considered cost-effective. However, all analyses show a high degree of uncertainty suggestive of a need for further research to allow conclusions to be drawn.

ACKNOWLEDGEMENTS

We would like to thank all PPI contributors, participants, research and clinical staff, the NIHR Clinical Research Network, and the members of the Trial Steering Committee and Data Monitoring Committee for their support.

The study was developed with support from the UK Dermatology Clinical Trials Network (UK DCTN). The UK DCTN is grateful to the British Association of Dermatologists and the University of Nottingham for financial support of the Network.

The University of Southampton was the research sponsor for this trial.

CONTRIBUTORSHIP STATEMENT

MS, AL, BS, THS, MJR, NF, KST, PL, JN, GG and IM conceived the study idea and initial study design in response to a NIHR HTA call, with later input from KLT, IS, ZE, SR, ML, NP and SP. All authors contributed to the acquisition of data. Specific advice was given by BS on trial design and medical statistics; and THS on health economic evaluation. Economic analyses were conducted by SP and THS. All authors contributed to the interpretation of data and drafting of this paper, led by SP and THS, and approved the final manuscript.

For peer review only

COMPETING INTERESTS

We declare no support from any organisation other than the NIHR for the submitted work; no financial relationships with any organisations that might have an interest in the submitted work; no other relationships or activities that could appear to have influenced the submitted work.

LH has received consultancy fees from the University of Oxford on an educational grant funded by Pfizer, unrelated to the submitted work. THS was a member of NIHR HTA Efficient Study Designs - 2, HTA Efficient Study Designs Board, HTA End of Life Care and Add-on-Studies, HTA Primary Care Themed Call Board and the HTA Commissioning Board between 2013 to Dec 2019. She is a steering committee member of the UK Dermatology Clinical Trials Network and Chair of the NIHR Research for Patient Benefit Regional Advisory Panel for the East of England. THS had no part in the decision making for funding this study.

FUNDING

This study presents independent research funded by the National Institute for Health and Care Research (NIHR) under its Health Technology Assessment programme (16/13/02). The views expressed are those of the author(s) and not necessarily those of the NHS, the NIHR or the Department of Health and Social Care.

This trial was registered prospectively with the ISRCTN registry (ISRCTN12892056) and EudraCT (2018-003630-33).

DATA SHARING STATEMENT

Consent was not obtained from participants for data sharing but authors will consider reasonable requests to make relevant anonymised participant level data available via the Southampton Clinical Trials Unit Data Sharing Committee.

ETHICS STATEMENT

This study was conducted in compliance with the ethical principles of the Declaration of Helsinki and in compliance with all International Council for Harmonisation Good Clinical Practice guidelines. The study protocol was reviewed and approved by the Institutional Review board and/or Independent Ethics Committee at each participating centre. All participants provided written informed consent.

Ethical approval for the trial was given by Wales Research Ethics Committee (REC) 3 in January 2019 (18/WA/0420).

SUPPORTING INFORMATION

Additional supporting information may be found in the online version of this article at the publisher's website:

Appendix S1: Participant Resource Use Questionnaire

Appendix S2 Supplementary material: further sensitivity and sub-group analyses

Figure S1 Intervention resource use as delivered via secondary care, per trial protocol

For peer review only - http://bmjopen.bmj.com/site/about/guidelines.xhtml

Table S1 Estimates of mean baseline resource use by treatment group (available case data)

Table S2 Estimates of mean change in cost (UK£ 2021/22) including wider costs, by treatment group

Table S3 Cost utility analyses and cost-effectiveness analyses results, for additional sensitivity analyses and sub-group analysis

REFERENCES

1 Purdy S, de Berker D. Acne. *BMJ* 2006;**333**:949–53. doi:10.1136/bmj.38987.606701.80

2 Schofield J, Grindlay D, Williams H. Skin conditions in the UK: a Health Care Needs Assessment. University of Nottingham: 2009.

3 Cunliffe WJ. Acne and unemployment. *Br J Dermatol* 1986;**115**:386–386. doi:10.1111/J.1365-2133.1986.TB05757.X

4 Jowett S, Ryan T. Skin disease and handicap: an analysis of the impact of skin conditions. *Soc Sci Med* 1985;**20**:425–9. doi:10.1016/0277-9536(85)90021-8

5 Mavranezouli I, Daly CH, Welton NJ, *et al.* A systematic review and network meta-analysis of topical pharmacological, oral pharmacological, physical and combined treatments for acne vulgaris. *Br J Dermatol* 2022;**187**:639–49. doi:10.1111/BJD.21739

6 Mavranezouli I, Welton NJ, Daly CH, *et al.* Cost-effectiveness of topical pharmacological, oral pharmacological, physical and combined treatments for acne vulgaris. *Clin Exp Dermatol* Published Online First: 30 July 2022. doi:10.1111/ced.15356

7 NICE. Acne vulgaris: management NICE guideline. 2021. www.nice.org.uk/guidance/ng198 (accessed 16 Nov 2022).

8 le Cleach L, Lebrun-Vignes B, Bachelot A, *et al.* Guidelines for the management of acne: recommendations from a French multidisciplinary group. *Br J Dermatol* 2017;**177**:908–13. doi:10.1111/BJD.15843

9 Thiboutot DM, Dréno B, Abanmi A, *et al.* Practical management of acne for clinicians: An international consensus from the Global Alliance to Improve Outcomes in Acne. *J Am Acad Dermatol* 2018;**78**:S1-S23.e1. doi:10.1016/J.JAAD.2017.09.078

10 Zaenglein AL, Pathy AL, Schlosser BJ, *et al.* Guidelines of care for the management of acne vulgaris. *J Am Acad Dermatol* 2016;**74**:945-973.e33. doi:10.1016/J.JAAD.2015.12.037

11 Platt D, Muller I, Sufraz A, *et al.* GPs' perspectives on acne management in primary care: a qualitative interview study. *Br J Gen Pract* 2020;**71**:E78–84. doi:10.3399/BJGP20X713873

12 Layton AM, Eady EA, Whitehouse H, *et al.* Oral Spironolactone for Acne Vulgaris in Adult Females: A Hybrid Systematic Review. *Am J Clin Dermatol* 2017;**18**:169–91. doi:10.1007/S40257-016-0245-X

For peer review only - http://bmjopen.bmj.com/site/about/guidelines.xhtml

13 Barbieri Lab. Spironolactone versus doxycycline for acne: a comparative non-inferiority evaluation (SD-ACNE) research study. https://barbierilab.bwh.harvard.edu/clinical-trial-opportunities/ (accessed 17 Nov 2022).

14 Poinas A, Lemoigne M, Le Naour S, *et al.* FASCE, the benefit of spironolactone for treating acne in women: study protocol for a randomized double-blind trial. *Trials* 2020;**21**:571. doi:10.1186/S13063-020-04432-W

15 Renz S, Chinnery F, Stuart B, *et al.* Spironolactone for adult female acne (SAFA): protocol for a double-blind, placebo-controlled, phase III randomised study of spironolactone as systemic therapy for acne in adult women. *BMJ Open* 2021;**11**:e053876. doi:10.1136/BMJOPEN-2021-053876

16 Santer M, Lawrence M, Renz S, *et al.* Effectiveness of spironolactone for women with acne vulgaris (SAFA) in England and Wales: pragmatic, multicentre, phase 3, double blind, randomised controlled trial. *BMJ* 2023;**BMJ-2022-074349**:e074349. doi:10.1136/bmj-2022-074349

17 Drummond M, Sculpher M. Common methodological flaws in economic evaluations. *Med Care* 2005;**43**:5–14. doi:10.1097/01.mlr.0000170001.10393.b7

18 Thiede RM, Rastogi S, Nardone B, *et al.* Hyperkalemia in women with acne exposed to oral spironolactone: A retrospective study from the RADAR (Research on Adverse Drug Events and Reports) program. *Int J Womens Dermatol* 2019;**5**:155–7. doi:10.1016/J.IJWD.2019.04.024

19 Plovanich M, YuWeng Q, Mostaghimi A. Low Usefulness of Potassium Monitoring Among Healthy Young Women Taking Spironolactone for Acne. *JAMA Dermatol* 2015;**151**:941–4. doi:10.1001/JAMADERMATOL.2015.34

20 Wang Y, Lipner SR. Retrospective analysis of adverse events with spironolactone in females reported to the United States Food and Drug Administration. *Int J Womens Dermatol* 2020;**6**:272. doi:10.1016/J.IJWD.2020.05.002

21 NHS Business Service Authority. Prescription Cost Analysis - England 2020/21. 2021. https://www.nhsbsa.nhs.uk/statistical-collections/prescription-cost-analysis-england/prescription-cost-analysis-england-202021 (accessed 30 Nov 2022).

22 Herdman M, Gudex C, Lloyd A, *et al.* Development and preliminary testing of the new five-level version of EQ-5D (EQ-5D-5L). *Qual Life Res* 2011;**20**:1727–36. doi:10.1007/s11136-011-9903-x

23 van Hout B, Janssen MF, Feng YS, *et al.* Interim scoring for the EQ-5D-5L: Mapping the EQ-5D-5L to EQ-5D-3L value sets. *Value in Health* 2012;**15**:708–15. doi:10.1016/j.jval.2012.02.008

24 NICE. Guide to the methods of technology appraisal 2013. 2013. doi:10.2165/00019053-200826090-00002

For peer review only - http://bmjopen.bmj.com/site/about/guidelines.xhtml

25 Manca A, Hawkins N, Sculpher MJ. Estimating mean QALYs in trial-based cost-effectiveness analysis: The importance of controlling for baseline utility. *Health Econ* 2005;**14**:487–96. doi:10.1002/hec.944

26 Fehnel SE, McLeod LD, Brandman J, *et al.* Responsiveness of the Acne-Specific Quality of Life Questionnaire (Acne-QoL) to treatment for acne vulgaris in placebo-controlled clinical trials. *Qual Life Res* 2002;**11**:809–16. doi:10.1023/A:1020880005846

27 Martin AR, Lookingbill DP, Botek A, *et al.* Health-related quality of life among patients with facial acne -- assessment of a new acne-specific questionnaire. *Clin Exp Dermatol* 2001;**26**:380–5. doi:10.1046/J.1365-2230.2001.00839.X

28 Willan AR, Briggs AH, Hoch JS. Regression methods for covariate adjustment and subgroup analysis for non-censored cost-effectiveness data. *Health Econ* 2004;**13**:461–75. doi:10.1002/hec.843

29 Drummond M, Sculpher M, Claxton K, *et al. Methods for the economic evaluation of health care programmes*. 4th ed. Oxford, UK: : Oxford University Press 2015.

30 Ramsey SD, Willke RJ, Glick H, *et al.* Cost-effectiveness analysis alongside clinical trials II - An ISPOR good research practices task force report. *Value in Health* 2015;**18**:161–72. doi:10.1016/j.jval.2015.02.001

31 Faria R, Gomes M, Epstein D, *et al.* A Guide to Handling Missing Data in Cost-Effectiveness Analysis Conducted Within Randomised Controlled Trials. *Pharmacoeconomics* 2014;**32**:1157–70. doi:10.1007/s40273-014-0193-3

32 Xu J, Mavranezouli I, Kuznetsov L, *et al.* Management of acne vulgaris: summary of NICE guidance. *BMJ* 2021;**374**:n1800. doi:10.1136/bmj.n1800

33 Leurent B, Gomes M, Faria R, *et al.* Sensitivity Analysis for Not-at-Random Missing Data in Trial-Based Cost-Effectiveness Analysis: A Tutorial. *Pharmacoeconomics* 2018;**36**:889–901. doi:10.1007/s40273-018-0650-5

34 Thorn JC, Davies CF, Brookes ST, *et al.* Content of Health Economics Analysis Plans (HEAPs) for Trial-Based Economic Evaluations: Expert Delphi Consensus Survey. *Value in Health* 2021;**24**:539–47. doi:10.1016/j.jval.2020.10.002

35 Yang Y, Brazier J, Longworth L. EQ-5D in skin conditions: an assessment of validity and responsiveness. *European Journal of Health Economics* 2015;**16**:927–39. doi:10.1007/s10198-014-0638-9

36 Klassen AF, Newton JN, Mallon E. Measuring quality of life in people referred for specialist care of acne: Comparing generic and disease-specific measures. *J Am Acad Dermatol* 2000;**43**:229–33. doi:10.1067/mjd.2000.105507

37 Jones K, Burns A. Unit Costs of Health and Social Care 2021. PSSRU. 2021.https://www.pssru.ac.uk/project-pages/unit-costs/unit-costs-of-health-and-social-care-2021/ (accessed 5 Dec 2022).

For peer review only

For peer review only - http://bmjopen.bmj.com/site/about/guidelines.xhtml

38 NHS England. 2019/2020 National Cost Collection Data Publication. London: 2021. https://www.england.nhs.uk/publication/2019-20-national-cost-collection-data-publication/ (accessed 5 Dec 2022).

39 Curtis L, Burns A. *Unit costs of health and social care 2015*. Personal Social Services Research Unit, University of Kent, Canterbury 2015.

40 UKHCA. UKHCA Commisioning Survey 2012: Care is not a Commodity. 2012. file://ueahome/eresfmh4/jry14qdu/data/Downloads/UKHCACommissioningSurvey2012.pdf (accessed 7 Aug 2023).

41 Brown R. PSNC Pharmacy Advice Audit 2021. 2021. https://cpe.org.uk/wp-content/uploads/2021/05/PSNC-Pharmacy-Advice-Audit-2021-Report.pdf (accessed 7 Aug 2023).

42 Office for National Statistics. Annual Survey of Hours and Earnings time series of selected estimates. 2021.https://www.ons.gov.uk/employmentandlabourmarket/peopleinwork/earningsandworkinghours/datasets/ashe1997to2015selectedestimates (accessed 5 Dec 2022).

FIGURES

Figure 1 | Intervention resource use as per standard treatment with spironolactone (base-case)

Figure 2 | Cost Effectiveness Acceptability Curve (CEAC), complete case analysis, adjusted and unadjusted QALYs

For peer review only

For peer review only - http://bmjopen.bmj.com/site/about/guidelines.xhtml

TABLES

Table 1 | Unit costs (UK£ sterling, 2020/21 financial year)

Cost Item	Unit Cost (£)	Unit	Source, assumptions
**Intervention**			
Spironolactone with dose escalation	£49.37	Total	Prescription Cost Analysis 2021.[21]
GP visit related to intervention	£33.00	Total	PSSRU Unit costs 2021.[37]
Blood test for renal function (eGFR) and potassium level (K serum)	£5.22	Total	National Cost Collection 2020.[38]*
**Medication costs**	Mean cost per quantity		
Topical preparations for acne	£0.96	gram/ml	Prescription Cost Analysis 2021.[21]<br>Mean across all medications in each medication type. Weighted averages taken where listed >1x.<br>Weighted average for estimating oral antibiotic control for SA (see table 3). Assumes 1x100 mg (doxycycline)/408 mg (lymecycline) per day for 24 weeks.
Other topical preparation	£0.03	gram/ml	
Oral contraceptives	£0.08	tablet	
Oral antibiotics	£0.22	capsule/tablet	
Anti-depressants	£0.20	capsule/tablet	
Analgesics	£0.04	capsule/tablet	
PCOS/diabetes medication	£0.03	tablet	
Other medications	£0.40	various	
Doxycycline/lymecycline weighted average	£0.25	Capsule	
**Community-based HCP contacts**			
GP visit unrelated to intervention	£33.00	Visit	PSSRU Unit costs 2021.[37]
Practice Nurse	£14.13	Visit	PSSRU Unit costs 2021 & 2015.[37,39]
NHS Walk-in centre	£71.99	Visit	National Cost Collection 2020.[38] Weighted average of all community health services.*
Community dermatology service	£121.01	Visit	National Cost Collection 2020.[38]*
Healthcare assistant	£14.44	Visit	PSSRU Unit Costs 2021[37] & UKHCA Commissioning Survey 2012.[40]
Pharmacist	£6.99	Visit	PSSRU Unit costs 2021 & 2015[37,39] & PSNC Pharmacy Advice Audit 2021.[41]
Physiotherapist	£66.82	Visit	National Cost Collection 2020.[38]*
Dietician	£82.46	Visit	National Cost Collection 2020.[38]*
Other (community)	£33.00	Visit	PSSRU Unit costs 2021. Used most common visit: GP visit.[37]
**Hospital out-patient contacts**			
Dermatologist	£128.25	Visit	National Cost Collection 2020.[38]*

For peer review only

Dermatology Nurse	£100.71	Visit	National Cost Collection 2020.[38]*
Ear, nose and throat (ENT)	£116.11	Visit	National Cost Collection 2020.[38]*
Interventional radiology	£137.64	Visit	National Cost Collection 2020.[38]*
Trauma and orthopaedics	£125.67	Visit	National Cost Collection 2020.[38]*
Respiratory medicine	£161.07	Visit	National Cost Collection 2020.[38]*
Other (out-patient)	£137.10	Visit	National Cost Collection 2020.[38]*
**Hospital admission**			
Accident and emergency	£182.28	Visit	National Cost Collection 2020. Index/Accident & Emergency.[38]*
**Wider costs**			
Personal out-of-pocket expenses	Various	Per item	Participant reported.
Lost work time	£18.01	Hour	ONS 2021.[42] Mean hourly earnings, excluding overtime (£).

SA, sensitivity analysis; ONS, Office for National Statistics; PSSRU, Personal Social Services Research Unit.

*Inflated to 2021 prices as per NHSCII Pay & Prices.[37]

For peer review only

Table 2 | Estimates of mean change in resource use and cost (UK£ 2021/22) and mean utility and QALY gain by treatment group (based on available case data)

Resource	Spironolactone (N=201)		No active systemic treatment (N=209)		Mean difference
	Mean (n)	SD	Mean (n)	SD	(95% CI)
Resource use over 24-week period:					
Spironolactone (number)	294 (201)	0	0 (209)	0	-
GP visits related to intervention (no. of visits)*	2.06 (201)	0.34	0 (209)	0	-
Blood tests – renal function (eGFR) and potassium level (number)	1.06 (201)	0.34	0 (209)	0	-
Total community-based HCP visits (number)	0.15 (150)	0.51	0.10 (124)	0.43	0.05 (-0.06 to 0.16)
Total hospital contacts (number)	0.06 (132)	0.30	0.05 (115)	0.26	0.01 (-0.06 to 0.08)
All prescription medications (number)	11.42 (147)	29.65	23.36 (124)	96.80	-11.94 (-28.51 to 4.63)
Total out-of-pocket items	3.59 (131)	5.96	4.49 (113)	6.67	-0.90 (-2.49 to 0.69)
Lost patient work time (number reporting)	0.00 (186)	0.00	0.02 (191)	0.144	-0.02 (-0.04 to -0.00)
Lost carer work time (number reporting)	0.01 (185)	0.07	0.02 (190)	0.144	-0.02 (-0.04 to 0.01)
Costs over 24-week period (UK£2021/22):					
All intervention costs	122.87 (201)	13.04	0 (209)	0	122.87 (121.09 to 124.64)
All community-based HCP costs	6.28 (150)	24.83	3.75 (124)	16.46	2.53 (-2.60 to 7.66)
All hospital contact costs	7.28 (132)	36.42	5.73 (115)	28.09	1.55 (-6.70 to 9.79)
All prescription medication costs	4.37 (147)	11.77	5.91 (124)	18.93	-1.54 (-5.25 to 2.17)
**Total costs**	**141.99 (128)**	**57.90**	**15.64 (110)**	**45.62**	**126.35 (112.88 to 139.82)**
**Total costs excluding intervention**	**19.61 (128)**	**56.65**	**15.64 (110)**	**45.62**	**3.98 (-9.30 to 17.26)**
Total out-of-pocket costs	69.41 (139)	113.05	82.57 (120)	148.60	-13.15 (-45.23 to 18.92)
Lost patient and carer productivity	27.87 (177)	354.76	15.95 (179)	183.54	11.93 (-46.86 to 70.71)
**Total costs (wider perspective)**	**252.67 (113)**	**490.19**	**93.53 (100)**	**144.02**	**159.14 (58.86 to 259.41)**
EQ-5D score (CUA)					
Baseline	0.887 (200)	0.148	0.860 (209)	0.200	0.027 (-0.008 to 0.061)
6 weeks	0.894 (176)	0.135	0.863 (179)	0.168	0.031 (-0.001 to 0.063)
12 weeks	0.904 (174)	0.138	0.877 (166)	0.177	0.027 (-0.007 to 0.061)
24 weeks	0.909 (163)	0.153	0.890 (136)	0.180	0.019 (-0.019 to 0.057)
**Total QALY score over 24 weeks**	**0.417 (162)**	**0.058**	**0.404 (136)**	**0.079**	**0.013 (-0.002 to 0.029)**
Acne-QoL symptom sub-scale score (CEA)					
Baseline	13.22 (201)	4.94	12.87 (209)	4.55	0.35 (-0.57 to 1.27)
6 weeks	16.97 (176)	5.72	15.65 (179)	5.69	1.32 (0.13 to 2.51)
12 weeks	19.21 (176)	6.12	17.76 (166)	5.58	1.45 (0.20 to 2.69)
24 weeks	21.22 (163)	5.86	17.39 (136)	5.80	3.83 (2.49 to 5.16)
**Change at 24-weeks from baseline**	**8.15 (163)**	**6.12**	**4.46 (136)**	**6.34**	**3.68 (2.26 to 5.11)**

*Assumes that if spironolactone is found effective it would be prescribed in primary care.

For peer review only - http://bmjopen.bmj.com/site/about/guidelines.xhtml

Table 3 | Cost utility analyses and cost-effectiveness analyses results, including sensitivity analyses and sub-group analysis

CUA Analysis (N s, N p)	Incremental cost (95% CI)	Incremental QALYs (95% CI)	ICER	CEAC at £20,000 (£30,000) threshold*
Base-case^, CCA, adjusted (118,101)	125.36 (111.13 to 139.58)	0.0019 (-0.0096 to 0.0133)	£67,191	23% (35%)
Base-case^, CCA, unadjusted (126,109)	125.53 (112.15 to 138.91)	0.0036 (-0.0117 to 0.0189)	£34,770	37% (47%)
SA1^, Multiple imputation, adjusted (201,209)	119.78 (107.99 to 131.57)	0.0043 (-0.0041 to 0.0127)	£27,879	35% (53%)
SA2: Secondary care delivery, CCA, adjusted (118,101)	265.67 (250.52 to 280.82)	0.0019 (-0.0096 to 0.0133)	£141,955	3% (12%)
SA3a, oral antibiotic control, CCA, adjusted (118,101)	17.11 (2.88 to 31.33)	Threshold analysis value†: 0.00057		
SA3b, oral antibiotic control, MI, adjusted (201, 209)	11.53 (-0.26 to 23.32)	Threshold analysis value†: 0.00038		
SA4a: Wider perspective, CCA, adjusted (97,85)	102.07 (64.21 to 139.92)	-0.0027 (-0.0139 to 0.0085)	Dominated	9% (15%)
SA4b: Wider perspective, MI, adjusted (201,209)	133.25 (72.52 to 193.93)	0.0044 (-0.0041 to 0.0129)	£30,249	31% (50%)
**CEA Analysis (N s, N p)**	**Incremental cost (95% CI)**	**Incremental Acne-QoL symptom (95% CI)**	**Incremental cost per unit change**	-
Secondary analysis^, CCA, adjusted: (119,102)	126.57 (112.35 to 140.78)	3.31 (1.90 to 4.72)	£38.21	-
Secondary analysis^, CCA, unadjusted (127,110)	126.52 (113.00 to 140.04)	3.52 (1.94 to 5.11)	£35.91	-

^ comparing spironolactone plus routine topical treatment to no active systemic treatment plus routine topical treatment; 95% CI=95% confidence interval; ICER =incremental cost-effectiveness ratio; N s / N p =Number randomised to spironolactone / Placebo who were included in the analysis; CCA = complete case analysis; SA refers to the different sensitivity analyses described in the Methods; QALY=Quality Adjusted Life Years; *probability of being cost-effective at a the threshold (λ) of £20,000 and £30,000 per QALY. Adjusted analyses, adjusted for stratification variables (centre, baseline severity [IGA<3 vs. ≥3]) and baseline variables (Acne QoL symptom subscale score, use of topical treatments, utility score based on EQ-5D, total costs). †Threshold analysis conducted using a £30,000 threshold, as described in the methods. The value given represents the incremental QALY benefit below which spironolactone compared with oral antibiotic would switch from cost-effective to not cost-effective.

For peer review only - http://bmjopen.bmj.com/site/about/guidelines.xhtml

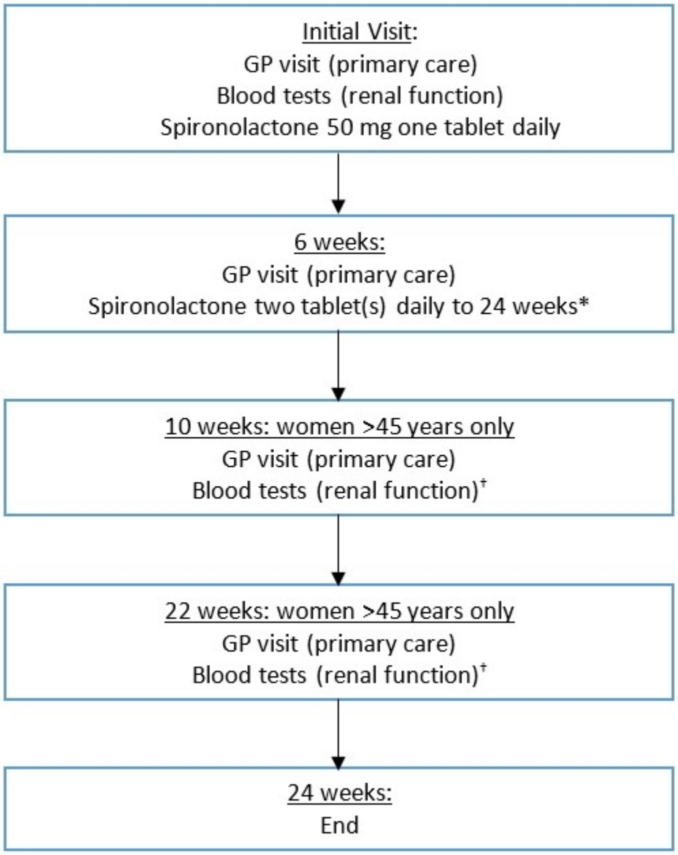

*Assumes all patients escalated to two 50 mg tablets spironolactone at 6-week visit. Based on the data from the trial, this was the case for 182/184 (99%) in the spironolactone group at 6 weeks (question response rate 184/201 in spironolactone group).

†Existing evidence and expert opinion recommend ongoing blood monitoring for women aged >45 years, or those with relevant comorbidities or on treatments with increased risk. As the latter two were not included in the trial, it is not possible to estimate the proportion of such patients that might receive spironolactone and need blood test monitoring. 6/201 (3%) patients in the spironolactone arm of the trial were aged >45 years.

Figure 1: Intervention resource use as per standard treatment with spironolactone (base-case)

190x232mm (96 x 96 DPI)

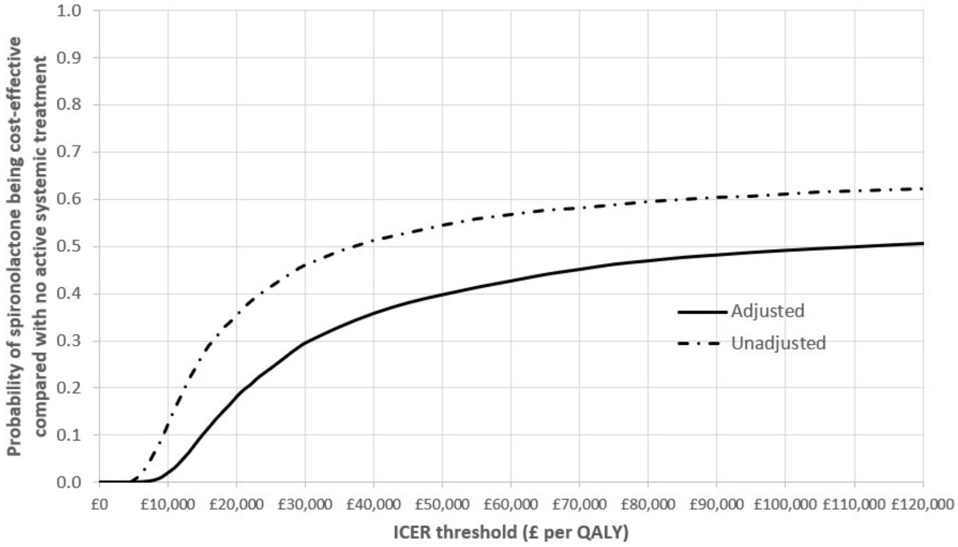

Figure 2: Cost Effectiveness Acceptability Curve (CEAC), complete case analysis, adjusted and unadjusted QALYs

246x145mm (96 x 96 DPI)

SUPPORTING INFORMATION

APPENDIX S2: FURTHER SENSITIVITY AND SUBGROUP ANALYSES

The following information is presented in addition to the main paper, "Cost-effectiveness of Spironolactone for Adult Female Acne (SAFA): Economic evaluation alongside a randomised controlled trial", published in The BMJ.[1] In addition to the sensitivity analyses presented in the main paper, a further two sensitivity analyses and a sub-group analysis were agreed before analysis and conducted to explore key uncertainties around the parameters of the economic evaluation. The details of these are outlined below.

Baseline Resource use

Table S1 presents the levels of resource use, at baseline, prior to randomisation (Table S1).

Sensitivity analysis: costing the intervention as per the SAFA trial protocol

Figure S1 describes the per protocol intervention resource use, undertaken in the trial and used to inform sensitivity analysis 2 (SA2). Subgroup analysis by age

A single sub-group analysis was undertaken for age (categorised as below 25 years and 25 years and over) as the clinical analysis found age significantly interacts with the outcome.[1]

The ICER was £263,871 per QALY for women under 25 years compared to £19,994 for women over 25 years of age (see Table S2). This result suggests that spironolactone is likely to be cost effective for women aged over 25 years. Whilst this finding is in line with the clinical findings, it ought to be interpreted with caution given the small sample sizes necessitated by splitting the dataset into subgroups combined with missing data.

Costs and outcomes over 52 weeks

Data was also collected beyond the treatment period (24 weeks) for up to 52 weeks. Response rates were, however, significantly lower at this time point, with 58% of participants missing EQ-5D data and 93% missing resource use data (see Supplementary Table S3). It is difficult to draw conclusions from these data, but incremental QALYs over 52 weeks was 0.0644 (95%CI 0.0093 to 0.1194) and incremental cost (NHS perspective) (see Supplementary Table S4) over the same period was £95.44 (95% CI 8.29 to 182.70).

Reference:

1. Santer M, Lawrence M, Renz S, *et al.* Effectiveness of spironolactone for women with acne vulgaris (SAFA) in England and Wales: pragmatic, multicentre, phase 3, double blind, randomised controlled trial. *BMJ* 2023;**BMJ-2022-074349**:e074349. doi:10.1136/bmj-2022-074349

SUPPLEMENTARY FIGURES

Supplementary Figure S1 | Intervention resource use as delivered via secondary care per trial protocol

For peer review only

SUPPLEMENTARY TABLES

Supplementary Table S1 | Estimates of mean baseline resource use by treatment group (available case data)

Resource	Spironolactone (N=201)		No active systemic treatment (N=209)		Mean difference
	Mean (n)	Std dev	Mean (n)	Std dev	(95% CI)
Total community-based HCP visits	0.27 (200)	0.616	0.225 (209)	0.590	0.045 (-0.072 to 0.162)
Total hospital contacts	0.119 (193)	0.446	0.095 (200)	0.396	0.024 (-0.059 to 0.108)
All medications – quantity (number)	11.711 (201)	46.065	7.903 (206)	21.570	3.809 (-3.174 to 10.791)
Total out-of-pocket items	2.027 (188)	2.735	1.939 (196)	2.438	0.088 (-0.432 to 0.607)
Lost patient work time (number reporting)	0.020 (197)	0.141	0.034 (205)	0.182	-0.014 (-0.046 to 0.018)
Lost carer work time (number reporting)	0.015 (194)	0.124	0.030 (203)	0.170	-0.014 (-0.044 to 0.015)

For peer review only

Supplementary Table S2 | Cost utility analyses and cost-effectiveness analyses results, for additional sub-group analysis

CUA Analysis (N s, N p)	Incremental cost (95% CI)	Incremental QALYs (95% CI)	ICER	CEAC at £20,000 (£30,000) threshold*
Sub-group analysis: <25 years, CCA, adjusted: (28,29)	108.23 (89.09 to 127.37)	0.0004 (-0.0141 to 0.0150)	£263,871	25% (33%)
Sub-group analysis: ≥25 years, CCA, adjusted: (90,72)	133.06 (114.97 to 151.16)	0.0067 (-0.0079 to 0.0213)	£19,994	50% (62%)

95% CI=95% confidence interval; ICER =incremental cost-effectiveness ratio; N s / N p =Number randomised to spironolactone / Placebo who were included in the analysis; CCA = complete case analysis; SA refers to the different sensitivity analyses described in the Methods; QALY=Quality Adjusted Life Years; *probability of being cost-effective at a the threshold (λ) of £20,000 and £30,000 per QALY. Adjusted analyses, adjusted for stratification variables (centre, baseline severity [IGA<3 vs. ≥3]) and baseline variables (Acne QoL symptom subscale score, use of topical treatments, utility score based on EQ-5D, total costs)

For peer review only

Supplementary Table S3 | 1Proportion of Missing values (%) for key variables

Variable	Spironolactone	No active systemic treatment	Total
**Baseline variables**			
Treatment allocation	0	0	0
Centre	0	0	0
Baseline severity (IGA)	0	0	0
Acne-QoL symptom subscale score at baseline	0	0	0
Use of topical treatments (y/n)	1.00	0.48	0.73
EQ-5D at baseline	0.50	0.00	0.24
Costs at baseline	4.48	5.74	5.12
**Cost variables***			
Costs at 6 weeks	17.91	18.18	18.05
Costs at 12 weeks	14.43	23.44	19.02
Costs at 24 weeks	23.88	38.76	31.46
Costs at 52 weeks	92.54	94.26	93.41
**Outcome variables for health-related quality of life**			
EQ-5D at 6 weeks	12.44	14.35	13.41
EQ-5D at 12 weeks	13.43	20.57	17.07
EQ-5D at 24 weeks	20.40	33.49	27.07
EQ-5D at 52 weeks	54.73	61.72	58.29
**Outcome variables for Acne-related quality of life**			
Acne-QoL at 6 weeks	12.44	14.35	13.41
Acne-QoL at 12 weeks	12.44	20.57	16.59
Acne-QoL at 24 weeks	18.91	34.93	27.07
Acne-QoL at 52 weeks	52.74	61.24	57.07
**Outcomes for cost-utility and cost-effectiveness analyses***			
Total costs (treatment period)	36.32	47.38	41.95
Total QALYS (treatment period)	20.90	33.49	27.32
Change Acne-QoL symptoms (treatment period)	18.91	34.93	27.07

Treatment period = baseline to 24 weeks
*For base-case, i.e. NHS-related costs only

For peer review only

Supplementary Table S4 | Mean (Standard Deviation) Cost and Cost Difference (95% Confidence Interval) Per Patient up to 25-–52 weeks for the Intervention arm compared to usual care arm (in 2021 UK pounds sterling)

Resource	Spironolactone (N=201)		No active systemic treatment (N=209)		Mean difference
	Mean (n)	Std dev	Mean (n)	Std dev	(95% CI)
**Costs**					
All community-based HCP costs	19.64 (16)	33.25	33.24 (13)	42.00	-13.60 (-42.25 to 15.05)
Total hospital contacts cost	17.10 (15)	45.13	9.87 (13)	35.57	7.23 (-24.70 to 39.17)
All medication cost	4.81 (16)	11.23	9.66 (13)	19.41	-4.85 (-16.65 to 6.96)
Total costs (NHS perspective), 25–52 weeks	39.89 (15)	67.47	54.41 (12)	79.00	-14.52 (-72.57 to 43.52)
Total costs (NHS perspective), 0–52 weeks	179.21 (13)	76.99	83.76 (10)	123.54	95.44 (8.29 to 182.60)
**Outcomes**					
52 weeks EQ-5D-5L utility	0.9208 (92)	0.1516	0.8291 (79)	0.2664	0.0918 (0.0274 to 0.1561)
QALYs at 52 weeks	0.9158 (88)	0.1364	0.8515 (74)	0.2154	0.0644 (0.0093 to 0.1194)
52 weeks symptom Acne-QoL	21.634 (95)	6.257	19.963 (81)	5.697	1.671 (-0.122 to 3.464)
Symptom Acne QoL change at 52 weeks compared to baseline	8.613 (95)	7.154	6.951 (81)	6.500	1.663 (-0.385 to 3.710)

For peer review only

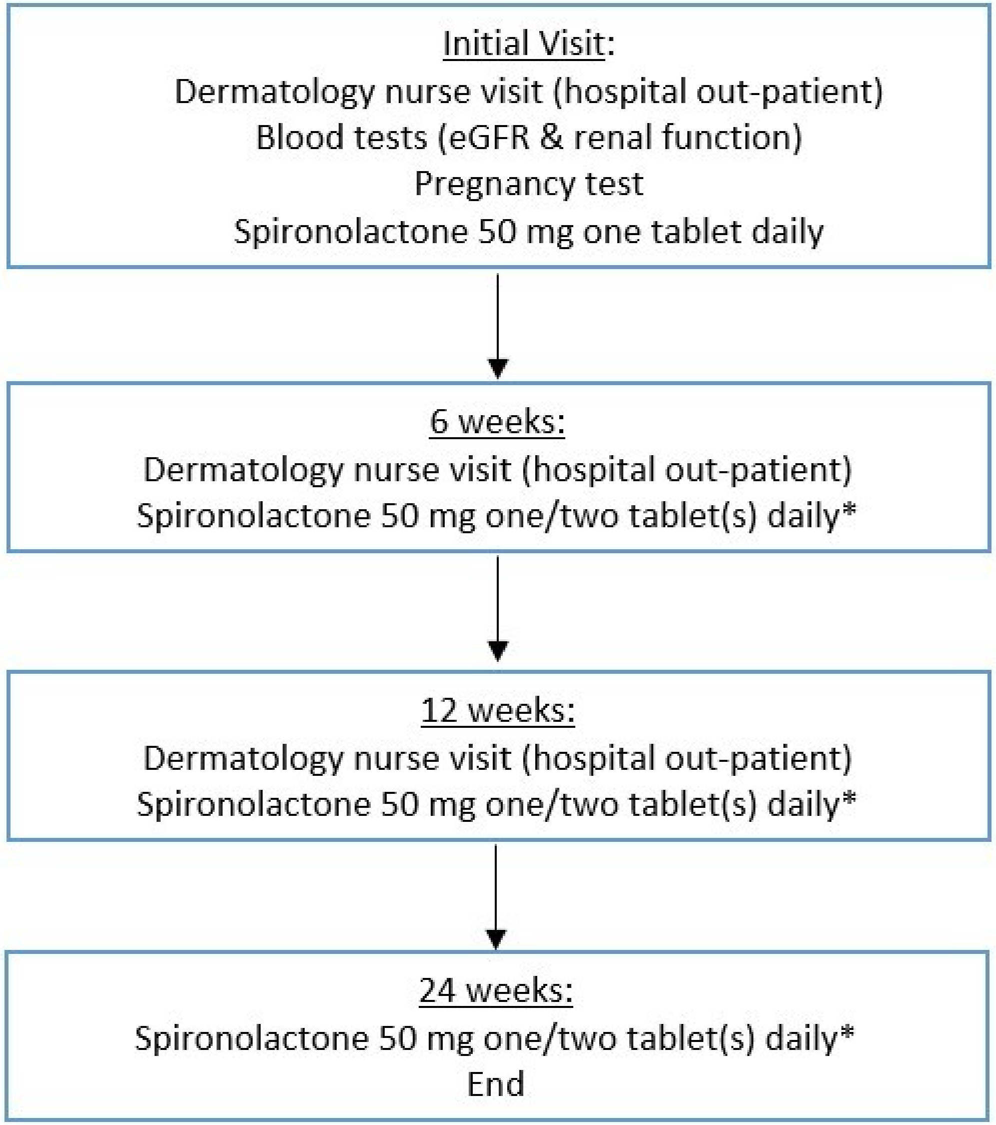

*At 6 weeks, dose was increased to 100 mg/day, assuming treatment was tolerated, which was the case for 182/184 (99%) of available patients in the spironolactone arm of the study

CHEERS 2022 Checklist – For SAFA Trial

	Item	Guidance for Reporting	Reported in section
**TITLE**			
Title	1	Identify the study as an economic evaluation and specify the interventions being compared.	Title and abstract
**ABSTRACT**			
Abstract	2	Provide a structured summary that highlights context, key methods, results and alternative analyses.	Abstract
**INTRODUCTION**			
Background and objectives	3	Give the context for the study, the study question and its practical relevance for decision making in policy or practice.	Introduction
**METHODS**			
Health economic analysis plan	4	Indicate whether a health economic analysis plan was developed and where available.	Methods
Study population	5	Describe characteristics of the study population (such as age range, demographics, socioeconomic, or clinical characteristics).	Results - Participant characteristics
Setting and location	6	Provide relevant contextual information that may influence findings.	Abstract, methods and results
Comparators	7	Describe the interventions or strategies being compared and why chosen.	Methods - Intervention and comparator. Sensitivity analysis 2 and 3.
Perspective	8	State the perspective(s) adopted by the study and why chosen.	Abstract and Methods
Time horizon	9	State the time horizon for the study and why appropriate.	Abstract and methods
Discount rate	10	Report the discount rate(s) and reason chosen.	Methods – Incremental analysis
Selection of outcomes	11	Describe what outcomes were used as the measure(s) of benefit(s) and harm(s).	Methods – measuring outcomes
Measurement of outcomes	12	Describe how outcomes used to capture benefit(s) and harm(s) were measured.	Methods – measuring outcomes
Valuation of outcomes	13	Describe the population and methods used to measure and value outcomes.	Methods – measuring outcomes
Measurement and valuation of resources and costs	14	Describe how costs were valued.	Methods – Measuring costs
Currency, price date, and conversion	15	Report the dates of the estimated resource quantities and unit costs, plus the currency and year of conversion.	Methods – Measuring costs
Rationale and description of model	16	If modelling is used, describe in detail and why used. Report if the model is publicly available and where it can be accessed.	N/A
Analytics and assumptions	17	Describe any methods for analysing or statistically transforming data, any extrapolation methods, and approaches for validating any model used.	Methods – Incremental analysis
Characterizing heterogeneity	18	Describe any methods used for estimating how the results of the study vary for sub-groups.	Supplementary material – Sub-group
Characterizing distributional effects	19	Describe how impacts are distributed across different individuals or adjustments made to reflect priority populations.	Not reported – this is a new item of the checklist which was not published at the time the study was designed.
Characterizing uncertainty	20	Describe methods to characterize any sources of uncertainty in the analysis.	Methods and supplementary analyses – sensitivity analyses
Approach to engagement with patients and others affected by the study	21	Describe any approaches to engage patients or service recipients, the general public, communities, or stakeholders (e.g., clinicians or payers) in the design of the study.	Methods - Patient and public involvement (PPI) and measuring costs
**RESULTS**			
Study parameters	22	Report all analytic inputs (e.g., values, ranges, references) including uncertainty or distributional assumptions.	Results
Summary of main results	23	Report the mean values for the main categories of costs and outcomes of interest and summarise them in the most appropriate overall measure.	Results
Effect of uncertainty	24	Describe how uncertainty about analytic judgments, inputs, or projections affect findings. Report the effect of choice of discount rate and time horizon, if applicable.	Results
Effect of engagement with patients and others affected by the study	25	Report on any difference patient/service recipient, general public, community, or stakeholder involvement made to the approach or findings of the study	Methods - Patient and public involvement (PPI) and measuring costs
**DISCUSSION**			

For peer review only

Study findings, limitations, generalizability, and current knowledge	26	Report key findings, limitations, ethical or equity considerations not captured, and how these could impact patients, policy, or practice.	Discussion
**OTHER RELEVANT INFORMATION**			
Source of funding	27	Describe how the study was funded and any role of the funder in the identification, design, conduct, and reporting of the analysis	Funding sources
Conflicts of interest	28	Report authors conflicts of interest according to journal or International Committee of Medical Journal Editors requirements.	Conflicts of interest

Husereau D, Drummond M, Augustovski F, de Bekker-Grob E, Briggs AH, Carswell C, Caulley L, Chaiyakunapruk N, Greenberg D, Loder E, Mauskopf J, Mullins CD, Petrou S, Pwu RF, Staniszewska S; CHEERS 2022 ISPOR Good Research Practices Task Force. Consolidated Health Economic Evaluation Reporting Standards 2022 (CHEERS 2022) Statement: Updated Reporting Guidance for Health Economic Evaluations. BMJ. 2022;376:e067975.
The checklist is Open Access distributed in accordance with the terms of the Creative Commons Attribution (CC BY 4.0) license, which permits others to distribute, remix, adapt and build upon this work, for commercial use, provided the original work is properly cited. See: http://creativecommons.org/licenses/by/4.0/.

For peer review only

BMJ Open

Cost-effectiveness of Spironolactone for Adult Female Acne (SAFA): Economic evaluation alongside a randomised controlled trial

Journal:	*BMJ Open*
Manuscript ID	bmjopen-2023-073245.R3
Article Type:	Original research
Date Submitted by the Author:	05-Sep-2023
Complete List of Authors:	Pyne, Sarah; University of East Anglia, Health Economics Group, Norwich Medical School<br>Sach, Tracey; University of East Anglia, Health Economics Group, Norwich Medical School; University of Southampton, School of Primary Care, Population Sciences and Medical Education<br>Lawrence, Megan; University of Southampton<br>Renz, Susanne; University Hospital Southampton NHS Foundation Trust<br>Eminton, Zina; University Hospital Southampton NHS Foundation Trust<br>Stuart, Beth; Queen Mary University of London, Centre for Evaluation and Methods Wolfson Institute of Population Health<br>Thomas, Kim; University of Nottingham, Dermatology<br>Francis, Nick; University of Southampton, Primary Care Research Centre<br>Soulsby, Irene; University of Southampton Faculty of Medicine, Primary Care and Population Sciences<br>Thomas, Karen ; University of Southampton, Public contributor, Primary Care Research Centre<br>Permyakova, Natalia; University of Southampton; University Hospital Southampton NHS Foundation Trust<br>Ridd, Matthew; University of Bristol, Population Health Sciences<br>Little, Paul; University of Southampton<br>Muller, Ingrid; University of Southampton<br>Nuttall, Jacqui; University of Southampton<br>Griffiths, Gareth; University of Southampton<br>Layton, Alison M; University of York, Skin Research Centre, Hull York Medical School<br>Santer, Miriam; University of Southampton, Primary Medical Care
**Primary Subject Heading**:	Health economics
Secondary Subject Heading:	Dermatology
Keywords:	Clinical Trial, Dermatology < INTERNAL MEDICINE, Health economics < HEALTH SERVICES ADMINISTRATION & MANAGEMENT, HEALTH ECONOMICS, Acne < DERMATOLOGY, Adult dermatology < DERMATOLOGY

SCHOLARONE™
Manuscripts

I, the Submitting Author has the right to grant and does grant on behalf of all authors of the Work (as defined in the below author licence), an exclusive licence and/or a non-exclusive licence for contributions from authors who are: i) UK Crown employees; ii) where BMJ has agreed a CC-BY licence shall apply, and/or iii) in accordance with the terms applicable for US Federal Government officers or employees acting as part of their official duties; on a worldwide, perpetual, irrevocable, royalty-free basis to BMJ Publishing Group Ltd ("BMJ") its licensees and where the relevant Journal is co-owned by BMJ to the co-owners of the Journal, to publish the Work in this journal and any other BMJ products and to exploit all rights, as set out in our licence.

The Submitting Author accepts and understands that any supply made under these terms is made by BMJ to the Submitting Author unless you are acting as an employee on behalf of your employer or a postgraduate student of an affiliated institution which is paying any applicable article publishing charge ("APC") for Open Access articles. Where the Submitting Author wishes to make the Work available on an Open Access basis (and intends to pay the relevant APC), the terms of reuse of such Open Access shall be governed by a Creative Commons licence – details of these licences and which Creative Commons licence will apply to this Work are set out in our licence referred to above.

Other than as permitted in any relevant BMJ Author's Self Archiving Policies, I confirm this Work has not been accepted for publication elsewhere, is not being considered for publication elsewhere and does not duplicate material already published. I confirm all authors consent to publication of this Work and authorise the granting of this licence.

For peer review only

TITLE:

Cost-effectiveness of Spironolactone for Adult Female Acne (SAFA): Economic evaluation alongside a randomised controlled trial

manuscript word count: 4,205/4,000 (recommended) Words
Table count: 3
Figure count: 2

AUTHORS:

Sarah Pyne,[1] Tracey H Sach*,[1,2] Megan Lawrence,[3] Susanne Renz,[3] Zina Eminton,[3] Beth Stuart,[2,4] Kim S Thomas,[5] Nick Francis,[2] Irene Soulsby,[6] Karen Thomas,[6] Natalia Permyakova,[3] Matthew J Ridd,[7] Paul Little,[4] Ingrid Muller,[2] Jacqueline Nuttall,[3] Gareth Griffiths,[3] Alison Layton,[8] Miriam Santer,[2]

[1] Health Economics Group, Norwich Medical School, University of East Anglia, Norwich, UK

[2] Primary Care Research Centre, School of Primary Care, Population Sciences and Medical Education, Faculty of Medicine, University of Southampton, Southampton, UK

[3] Southampton Clinical Trials Unit, University of Southampton and University Hospital Southampton NHS Foundation Trust, Southampton, UK

[4] Centre for Evaluation and Methods, Wolfson Institute of Population Health, Faculty of Medicine and Dentistry, Queen Mary University of London, London, UK

[5] Centre of Evidence Based Dermatology, School of Medicine, University of Nottingham, Nottingham, UK

[6] Public contributor, UK

[7] Population Health Sciences, Bristol Medical School, University of Bristol, Bristol, UK

[8] Skin Research Centre, Hull York Medical School, University of York, York, UK

***Corresponding author:** Professor Tracey Sach, Professor in Health Economics, School of Primary Care, Population Sciences and Medical Education, Faculty of Medicine, University of Southampton, Southampton, UK. Email: T.sach@Soton.ac.uk

For peer review only

For peer review only - http://bmjopen.bmj.com/site/about/guidelines.xhtml

ABSTRACT (299/300 WORDS)

Objective

This study aims to estimate the cost-effectiveness of oral spironolactone plus routine topical treatment compared with routine topical treatment alone for persistent acne in adult women from a British NHS perspective over 24-weeks.

Design

Economic evaluation undertaken alongside a pragmatic, parallel, double-blind, randomised trial.

Setting

Primary and secondary healthcare, community and social media advertising.

Participants

Women ≥18 years with persistent facial acne judged to warrant oral antibiotic treatment.

Interventions

Participants were randomised 1:1 to 50 mg/day spironolactone (increasing to 100mg/day after 6 weeks) or matched placebo until week-24. Participants in both groups could continue topical treatment.

Main outcome measures

Cost-utility analysis assessed incremental cost per Quality-Adjusted Life Year (QALY) using the EQ-5D-5L. Cost-effectiveness analysis estimated incremental cost per unit change on the Acne-QoL symptom subscale. Adjusted analysis included randomisation stratification variables (centre, baseline severity [IGA <3 versus ≥3]), and baseline variables (Acne-QoL symptom subscale score, resource use costs, EQ-5D score and use of topical treatments).

Results

Spironolactone did not appear cost-effective in the complete case analysis (n=126 spironolactone, n=109 control), compared with no active systemic treatment (adjusted incremental cost per QALY £67,191; unadjusted £34,770). Incremental cost per QALY was £27,879 (adjusted), just below the upper National Institute for Health and Care Excellence's (NICE) threshold value of £30,000, where multiple imputation took account of missing data. Incremental cost per QALY for other sensitivity analyses varied around the base-case, highlighting the degree of uncertainty. The adjusted incremental cost per point change on the Acne-QoL symptom subscale for spironolactone compared with no active systemic treatment was £38.21 (complete case analysis).

Conclusions

The results demonstrate a high level of uncertainty, particularly with respect to estimates of incremental QALYs. Compared with no active systemic treatment, spironolactone was estimated to be marginally cost-effective where multiple imputation was performed but was not cost-effective in complete case analysis.

For peer review only - http://bmjopen.bmj.com/site/about/guidelines.xhtml

STRENGTHS AND LIMITATIONS

- Our study is based on individual patient level data collected alongside the first large pragmatic, parallel, double-blind, randomised trial of spironolactone for acne.
- In addition to the base-case analysis seeking to answer the question of whether spironolactone is cost-effective compared with no active systemic treatment (both groups could use routine topical treatments) in women with persistent acne, a number of sensitivity analyses were undertaken to provide a range on estimates of cost-effectiveness under different scenarios.
- Differential rates of missing data between groups over time were addressed by undertaking both a complete case analysis and multiple imputation to explore the impact of missing data on the study conclusions.
- As the study was constrained by the design of the clinical trial, the base-case did not reflect real-world prescribing in the comparator group, limiting interpretation of the results.
- The results reflect the method of data collection and may have been limited as a consequence of resource-use under-reporting, short time-frame and limited sensitivity of the EQ-5D outcome measure in patients with acne.

INTRODUCTION

Acne (acne vulgaris) is a common condition, affecting >80% of people at some point in their life.[1] Its impact on the NHS is considerable, being responsible for around 3.5 million consultations with a GP[1] and 70,000 referrals for specialist care[2] in the UK annually. As well as direct burdens to the NHS, adults (18–30 years) with severe acne in the UK have higher unemployment rates[3] and a small study by Jowett and Ryan (1985)[4] showed that 45% (13/29) of acne patients reported interpersonal difficulties at work.

There are many treatment options for women with moderate-to-severe acne, but a recent network meta-analysis (NMA) demonstrated paucity of good quality evidence and the complexity of choice.[5] Informed in large part by this NMA and the associated economic model,[6] the National Institute of Health and Care Excellence (NICE) guidelines on the management of acne vulgaris recommend a fixed combination topical preparation containing retinoids, benzoyl peroxide or antibiotics as first-line treatment for any severity of acne, whilst a fixed combination topical agent plus oral lymecycline or doxycycline once daily is recommended for moderate-to-severe acne. The latter is also recommended for moderate-to-severe acne that does not respond adequately to a 12-week course of treatment that does not include an oral antibiotic.[7] The guidance states that treatment options including an antibiotic (topical or oral) should only be continued for more than 6 months in exceptional circumstances (other guidelines limit oral antibiotic duration to 3 months)[8–10] and that clinicians should be aware of the associated risks of antimicrobial resistance. Doctors, however, report many challenges when trying to discontinue oral antibiotics.[11]

Spironolactone is already used off license for women with acne, is an inexpensive treatment choice and could play a role in reducing antibiotic use.[12] Literature searches did not, however, find any previously published economic evaluations on the cost-effectiveness of spironolactone in this group of patients, although there are two other ongoing studies of spironolactone in France and the USA,

For peer review only - http://bmjopen.bmj.com/site/about/guidelines.xhtml

the former of which includes an economic evaluation.[13,14] In this paper we estimate the cost-effectiveness of spironolactone plus routine topical treatment compared with no active systemic treatment plus routine topical treatment for persistent acne in adult women from a British NHS perspective over 24-weeks.

PATIENTS AND METHODS

The Spironolactone for Adult Female Acne (SAFA) trial was a pragmatic, multicentre, participant-led, and clinician-blind, superiority, randomised trial with two parallel treatment groups: spironolactone compared to placebo in women aged 18 years and older with facial acne judged to warrant oral antibiotics. The economic evaluation was nested within this trial.

Participants were recruited in primary care, secondary care and through advertising (community and social media). Baseline assessment was conducted by a research nurse and/or dermatologist in secondary care clinics to ensure standard clinical assessments, as the Investigator's Global Assessment (IGA) for acne was an inclusion criterion and an important secondary outcome. Baseline appointments included a pregnancy test, blood test (to exclude renal impairment or raised serum potassium), participant photo to aid recall about changes in acne and contraceptive counselling. The first participant was recruited in June 2019 and the last in August 2021, whilst follow-up finished February 2022. The SAFA trial is described in more detail in the clinical paper.[15,16]

Participants were randomised 1:1 using online software to either 50 mg/day spironolactone or matched placebo until week-6, increasing to 100 mg/day spironolactone or matched placebo until week-24, assuming treatment was tolerated. Participants were stratified by recruitment centre and baseline acne severity (IGA<3 vs IGA≥ 3). In both groups participants could continue using topical treatment. Between baseline and week-12 participants were asked not to take oral treatment for acne other than study medication, except for oral contraception taken for over 3 months previously. After 12 weeks, participants in both groups could receive usual care, including oral treatments, such as oral antibiotics, hormonal treatment or isotretinoin. In both groups participants were followed up face-to-face (or by video call or telephone due to COVID-19) at week-6 and week-12 in secondary care, with primary outcome assessment at week-12, and longer-term follow-up by questionnaires at week-24.

Although in the clinical trial, spironolactone plus routine topical treatment was compared to placebo plus routine topical treatment, it is most appropriate in economic evaluations to compare an active treatment to current usual care.[17] Therefore, to utilise the data collected in the trial whilst reflecting a useful analysis to decision makers in practice, this economic evaluation compared spironolactone plus routine topical treatment to not active systemic treatment plus routine topical treatment.

Measuring costs

In keeping with an NHS perspective, all acne-related resource use data, including intervention, primary and secondary care visits, and prescription medication use, were collected for participants in both groups. Personal Social Services (PSS) resource use was not collected, as patient and clinician contributors did not anticipate these being incurred by participants.

For peer review only - http://bmjopen.bmj.com/site/about/guidelines.xhtml

Resource use data was collected via case report forms and participant questionnaires (see supplementary material Appendix S1 for a copy), designed with the input of public contributors, at baseline (collecting the preceding 6 weeks), week-6, week-12 and week-24 for the intervention phase.

Resource use was valued using UK unit costs (£ Sterling) for the most current price year available at the start of analysis (financial year 2021) and identified from published sources.

The intervention was costed as described in
Figure 1, which assumes that standard treatment with spironolactone, if adopted, will be delivered in primary care, including two GP visits (unless >45 years of age), baseline blood test and the cost of spironolactone (50 mg 6 weeks, 100 mg 18 weeks).[10,18–20] No intervention costs (placebo tablets, GP visits to prescribe placebo tablets or blood tests) were included for the no active systemic treatment group as these would not occur if no intervention was being given (the comparator for this economic evaluation).

Acne-related resource use data related to visits to community-based healthcare professionals (HCP), visits to hospital out-patient and in-patient services (including accident and emergency) and prescribed medication costs were self-reported via participant questionnaires at all time-points, including baseline for participants in both groups. When asked about medication use, participants were asked to report only what they had been prescribed since the previous follow-up visit. Unit costs for each visit-type were combined with this data to estimate the total community-based HCP visit costs and the total hospital contact costs. Participants were also asked for details of prescribed acne-related medication including type, strength and quantity. Unit costs for all medication types[21] were used to estimate the prescription costs over the 24-week treatment period.

The mean (sd) cost per participant per intervention group was estimated for the 24-week treatment period, for each of the cost types described above and mean difference (95% CI) in NHS cost was estimated.

Measuring outcomes
The primary economic outcome measure was QALYs over the trial period of 24 weeks, as measured by the generic preference-based EQ-5D-5L questionnaire.[22] Responses were converted to utility scores using the EQ-5D-5L Crosswalk UK preference weights, as this was in line with recommendations at the point analysis started, where utility ranges from -0.594 to 1.[23,24] Utility values were used to estimate QALYs over 24 weeks, using both linear interpolation and area under the curve analysis.[25]

A secondary economic outcome was the Acne-QoL symptom sub-scale score (five questions with seven responses to each)[26,27] at week-24, used as an estimate of effectiveness, which enables comparison with future economic studies in acne.

Economic analysis
The base-case cost-utility analysis (CUA) and secondary cost-effectiveness analysis (CEA) incorporated all randomised participants with complete cost and outcome data. Given the 24-week time-horizon, costs and benefits were not discounted.[24]

For peer review only - http://bmjopen.bmj.com/site/about/guidelines.xhtml

The base-case CUA estimated the incremental cost per QALY (incremental cost-effectiveness ratio, ICER) to enable comparison with the cost-utility of other interventions. The incremental cost (95% CI) and QALY change (95% CI) between groups was estimated unadjusted and adjusted for randomisation stratification variables (centre, baseline severity [IGA <3 versus ≥3]), and baseline variables (including Acne-QoL symptom subscale score, resource use costs, EQ-5D score and use of topical treatments (Y/N)). In line with NICE guidance,[24] we estimated whether the intervention was cost-effective by comparing the ICER with a cost-effectiveness threshold of £20,000 to £30,000 per QALY.

A CEA estimated the incremental cost per unit change on the Acne-QoL symptom sub-scale score. The incremental cost (95% CI) and Acne-QoL symptom sub-scale change (95% CI) between groups was estimated unadjusted and adjusted as described for the base-case CUA. The CUA and CEA were undertaken using a regression-based approach (seemingly unrelated regression equations).[28]

Published guidelines for the economic evaluation of health care interventions were followed as appropriate.[29,30]

To estimate the level of uncertainty associated with the decision regarding cost-effectiveness, Fieller's theorem was used to calculate[31] the probability of being cost-effective at the £20,000 and £30,000 willingness-to-pay threshold values.[24] Non-parametric bootstrapping was conducted to generate 10,000 estimates of incremental costs and benefits. From this, Cost-Effectiveness Acceptability Curves (CEACs) were generated to show the probability that the intervention is estimated to be cost-effective at different willingness-to-pay values.

Several sensitivity analyses were agreed and specified in the health economic analysis plan (HEAP) before analysis to explore key uncertainties around important parameters in the economic evaluation. The impact of missing data on cost-effectiveness estimates was explored by undertaking multiple imputation (SA1), assuming that the data was missing at random (MAR) and using chained equations to handle the missing cost and outcome data.[31] Secondly, the impact of costing the intervention as per the SAFA trial protocol (i.e. intervention was accessed via secondary care, excluding any research related costs) was explored (SA2). The cost utility analysis was repeated but with the intervention costed as described in Figure S1, while the placebo group was costed as in the base-case analysis, i.e. assumed no intervention costs. Thirdly, the CUA was repeated assuming that, as this patient population had persistent acne of sufficient severity to warrant treatment with oral antibiotics, all women in the no active systemic treatment group took oral antibiotics (lymecycline or doxycycline, 1 tablet daily for 24 weeks) as per NICE guidance[32] , in addition to topical treatment (SA3). To cost this intervention the weighted mean cost per dose of doxycycline/lymecycline was used (Table 1) and two GP visits assumed. Due to a lack of evidence about the incremental QALYs between spironolactone plus topical treatment versus oral antibiotics plus topical treatment a threshold analysis was performed to ascertain what level of incremental QALYs would switch the intervention between cost-effective and not cost-effective. Incremental costs (95% CI) and the threshold value for incremental QALYs are presented in the results. Potential costs associated with antibiotic-related side-effects and the societal costs of over prescribing of oral antibiotics were not included. Lastly, a sensitivity analysis exploring a wider perspective than that limited to the NHS was conducted (SA4). In addition to NHS-related resource use data, the following was collected via participant questionnaire: out-of-pocket expenses (including, complementary therapist visits,

For peer review only - http://bmjopen.bmj.com/site/about/guidelines.xhtml

cosmetic skin care products, non-NHS-prescribed medication, parking and travel costs for healthcare appointments and other) and productivity losses (including lost patient and carer productivity). These were valued using participant self-reported values and unit costs identified from published sources, as reported in Table 1, and summed along with NHS costs to estimate the mean difference (95% CI) in total costs (wider perspective). Utility analysis was then repeated as described for the base case. A sub-group analysis based on age was also conducted and is presented in supplementary material appendix S2.

Stata MP version 17 was used to conduct the analyses. A health economic analysis plan (HEAP) was written and followed; a copy is available from the corresponding author.

Patient and public involvement (PPI)

Key questions relating to research design were explored with a virtual acne-specific patient panel and patient survey carried out via the UK Dermatology Clinical Trials Network (UKDCTN). Two public contributors (IS and KaT) with experience of acne were members of the Trial Management group as part of this role they helped identify relevant resources and outcomes and how this data should be collected. They also contributed to the interpretation and write-up if the health economics component.

RESULTS

Participant characteristics

The clinical trial results, including details on sample size and participant characteristics, are reported elsewhere.[16] Of the 410 women recruited to the trial, 201 were randomly assigned to spironolactone and 209 allocated to placebo at the start of the trial. All were allowed to continue routine topical treatment. At week-24 126 women in the spironolactone group and 109 women in the placebo group had complete cost and outcome data, and these formed the base-case unadjusted CUA. Mean age was 29.2 years, mean BMI was 26.1, at baseline 83% (340/410) participants were using or had used topical treatments, and the majority (75% [306/410]) had acne for two or more years. There were no significant differences in characteristics between groups.[16]

Costs

The unit costs used in the analysis are presented in Table 1. The levels of resource use in each group were very similar prior to randomisation (Table S1).

The majority of responding women in the spironolactone group (182/184, 99%) increased to two tablets of spironolactone at week 6. The 'standard treatment' approach used in the base-case economic evaluation, gave rise to a mean total intervention resource use cost of £122.87 (SD £13.04) per participant in the spironolactone group (Table 2).

Using available case data, when intervention use was combined with other health resource use, the unadjusted mean incremental cost per participant was £126.35 (95% CI, £112.88 to £139.82) for women receiving spironolactone compared to women receiving no active systemic treatment in the base-case (Table 2). Excluding intervention costs, the difference was not significant between groups. While patients were asked about in-patient visits, none were reported.

For peer review only - http://bmjopen.bmj.com/site/about/guidelines.xhtml

Outcomes

The mean (sd) QALYs over 24 weeks in the spironolactone group were 0.417 (0.058) per participant compared to 0.404 (0.079) per participant in the no active systemic treatment group, giving an incremental difference of 0.013 (95% CI -0.0024 to 0.0289) QALYs using unadjusted available case data (Table 2). The wide 95% confidence intervals around mean estimates demonstrates a high degree of uncertainty.

The mean (sd) change from baseline in Acne-QoL symptom subscale score at 24 weeks was 8.15 (6.12) in the spironolactone group compared to 4.46 (6.34) in the no active systemic treatment group. Thus, the incremental difference in score was 3.68 (95% CI 2.26 to 5.11) in favour of the spironolactone group (Table 2).

Base-case Cost Utility Analysis

In the complete case analysis, the incremental cost for the spironolactone group (n=118) compared to the no active systemic treatment group (n=101) was £125.36 (95% CI, £111.13 to £139.58) (unadjusted this was £125.53 [95% CI £112.15 to £138.91]) (Table 3). The adjusted incremental QALYs for the spironolactone group compared with the no active systemic treatment group was 0.0019 (95% -0.0096 to 0.0133) (unadjusted was 0.0036, 95% CI -0.0117 to 0.0189). The ICER was £67,191 (unadjusted £34,770) per QALY. At a willingness to pay of £30,000 per QALY there was a 35% (unadjusted 47%) chance of spironolactone being cost-effective in this population of women with persistent acne.

The cost-effectiveness acceptability curves (**Error! Reference source not found.**) of the adjusted and unadjusted base-case analysis, show that the probability of spironolactone being cost-effective only approaches 50% as the threshold value approaches £120,000 (adjusted), demonstrating a high degree of uncertainty associated with the decision under these conditions.

Secondary Cost Effectiveness analysis

The adjusted incremental difference in cost per point change on the Acne-QoL symptom subscale for the spironolactone group (n=119) compared to no active systemic treatment group (n=102) was £38.21 (unadjusted £35.91) based on a complete case analysis (Table). How much a decision maker would be willing to pay for a point change on the Acne-Qol symptom subscale is unknown.

Sensitivity analyses

The results of the sensitivity analyses are presented in Table 3 and prove influential to the conclusions reached. The ICER varies around the base-case from £27,879 (with a 53% probability of being cost-effective at £30,000 threshold for the MI analysis (SA1) to spironolactone being dominated (more costly and less effective than control) for the wider perspective (CCA) analysis.

There were differential rates of attrition with greater missing data in the no active systemic treatment group, compared to spironolactone group, by 24-weeks follow-up, for costs (39% vs. 24%, respectively) and EQ-5D-5L (33% vs. 20%, respectively). This may offer some explanation for why, when using multiple imputation in a sensitivity analysis the ICER was less than in the complete case, adjusted analysis (Table 3).

With regards to the oral antibiotic control analysis (SA3), the planned threshold analysis using the complete case, adjusted data found that the incremental QALY benefit for spironolactone compared

For peer review only - http://bmjopen.bmj.com/site/about/guidelines.xhtml

with oral antibiotics would have to be 0.00057 (0.000384, MI adjusted) or less, over 24 weeks, for spironolactone to be less cost-effective than oral antibiotics at a £30,000 threshold. The plausibility of this value is unclear but research comparing spironolactone with oral antibiotics, currently underway[13] will enable an assessment of plausibility once published.

Of note regarding the wider perspective sensitivity analysis (SA4) The majority of women (97%) reported no impact on their employment as a result of their acne and thus it is mainly out-of-pocket expenses driving change from the base-case.

The results of a subgroup analysis undertaken for women aged <25 years and ≥25 years are reported in online supplementary material appendix S2. See Table S2 for results.

DISCUSSION

This economic study finds a high degree of uncertainty about whether spironolactone is likely to be cost-effective. Our economic evaluation provides a range of estimates for the cost effectiveness of spironolactone used alongside routine topical treatment. The base-case analysis, where the comparator is no active systemic treatment plus routine topical treatment, and the delivery of the intervention is costed as via primary care, spironolactone was not estimated to be cost-effective in the unadjusted and adjusted complete case analyses. However, in the adjusted analysis using multiple imputation (MI) the ICER was estimated to be just under the £30,000 per QALY threshold. This divergence in conclusion between the complete case and MI analysis demonstrates the impact of missing data (attrition bias) and suggests more weight ought to be placed on the MI analysis.[33] The results of other sensitivity analyses (Table 3) varied around the base-case, adding to the uncertainty of the results. [13]

This economic evaluation followed a Health Economic Analysis Plan finalised before data was received for analysis reducing bias in the results from selective reporting or cherry-picked analyses.[34] Another strength of this economic evaluation is that it can provide reliable estimates of cost effectiveness based on individual participant level data, collected at little marginal cost, alongside a randomised controlled trial. This is, however, also a limitation in that within trial health economic evaluations are constrained by the question, timeframe, and data collected, particularly in placebo-controlled trials. In particular there are five main limitations to acknowledge: (1) the assumptions required to compare spironolactone to inactive systemic treatment; (2) the assumptions required to undertake a sensitivity analysis using oral antibiotics as the comparator; (3) the sensitivity and validity of the EQ-5D-5L in patients with acne; (4) the time frame of the analysis; and (5) the use of complete case analysis rather than the analysis using multiple imputation to take account of missing data as the base case analysis. We look at these in turn below, but all should be borne in mind when interpreting the results.

Firstly, ideally economic evaluations should compare an active treatment to current usual care. The funder for this trial preferred the placebo comparator to current usual care.[17] We wanted our primary analysis to reflect as closely as possible the data collected in the actual trial whilst reflecting a useful analysis to decision making in practice. We therefore felt the most appropriate comparator would be no active systemic treatment, rather than placebo, which would not reflect reality. Placebos are not used in routine practice, but some evidence of placebo effects has been documented in acne.[5] Therefore, the base-case set out to answer the question of whether

For peer review only - http://bmjopen.bmj.com/site/about/guidelines.xhtml

spironolactone is cost-effective compared with no active systemic treatment (both groups could use routine topical treatments) to align with the clinical question funded. A limitation of this is that, because it does not account for the potential impact of a placebo effect, it may result in underestimation of the QALY gain with spironolactone compared with not providing spironolactone, and hence underestimate its cost-effectiveness. We also excluded the research costs associated with administering the placebo (costs of the pills and appointments to administer them) but did include ongoing costs associated with NHS resource use related to acne in both arms of the study. There is also uncertainty about how many, if any, additional GP visits might have occurred in the usual care group if they had actually received usual care as opposed to placebo during the trial. It is not possible to know how costs and effects would differ between our placebo group and a group without any active systemic treatment because we did not have the latter group in the study. We feel the assumptions made are required to make the analysis most useful to practice but acknowledge they may mean the estimates of the cost-effectiveness of spironolactone are conservative.

Secondly, in practice clinicians are unlikely to send women away with no active treatment if they consulted with acne persisting beyond 6 months. As advised by the trial clinicians, the clinically important comparator may be another systemic treatment rather than no active systemic treatment. To address this a sensitivity analysis assuming, for cost purposes, all women in the no active systemic treatment group received an oral antibiotic (in addition to topical treatments) for 24 weeks was planned. This analysis assumed that incremental QALYs remain the same as in the base-case analysis, which we acknowledge is unlikely. There is limited economic evidence comparing oral antibiotics in combination with routine topical treatment compared with routine topical treatment alone[5]. Despite these limitations and while the results of this sensitivity analysis should be interpreted with caution, considering the assumptions made, the analysis serves to provide a lower range estimate for the cost effectiveness of spironolactone that better reflects accepted standard-of-care, based upon current NICE guidelines.[32] Further evidence, from randomised controlled trials,[13,14] is required to determine whether this is a likely scenario and to draw conclusions.

Thirdly, the uncertainty highlighted by this study may be impacted, in part, by the method of measuring utility, an area where further research would be valuable. The conclusion reached about cost-effectiveness was sensitive to the estimates of QALYs generated from EQ-5D-5L, despite 46% in the intervention group and 43% in the control group reporting perfect health (EQ-5D-5L health state 11111) at baseline. For these participants, the EQ-5D-5L had no potential to measure improvements in health-related quality of life. This likely contributes to the wide 95% confidence intervals around the incremental QALY estimates in this study, which means we cannot be certain spironolactone improves QALYs rather than have no difference or worsen QALYs. At design stage, there was discussion about the possible use of other instruments, however, the limited published evidence supported the use of the EQ-5D for acne.[35,36] Like Klassen et al[36] we find that women with persistent acne report most problems on the pain/discomfort and anxiety/depression dimensions of the EQ-5D. Further research using the EQ-5D data generated in this study alongside that elicited in other studies of acne would help inform future studies about the validity and responsiveness of this instrument for acne.

Fourthly, we acknowledge that the analysis was conducted for a 24-week timeframe and that were a longer timeframe taken the cost-effectiveness of spironolactone may improve if, for instance, there

For peer review only - http://bmjopen.bmj.com/site/about/guidelines.xhtml

is a sustained effect once treatment stops. We sought to collect resource use and utility data up to 52 weeks but due to reduced data completion at 52 weeks (see supplementary material for details) it was not feasible to analyse results to a longer time horizon.[32]

Finally, a complete case analysis was specified in the Health Economic Analysis Plan as the base case analysis (with multiple imputation as a sensitivity analysis) reflecting a desire to be consistent with the approach undertaken in the Statistical Analysis Plan for the clinical primary outcome. With the benefit of hindsight primary concern ought to have been around the level of missing economic data, which is known to often be greater than that for clinical outcomes. However, both complete case and multiple imputation analyses are reported, as planned, so that the impact of missing data on the results can be clearly seen.

Our study provides estimates of the cost-effectiveness of spironolactone in women with persistent acne using the trial data and a range of scenarios. It highlights that there is considerable uncertainty about whether spironolactone is cost-effective and the need for further research with comparators more akin to clinical practice. The complete case analysis estimated ICERs in excess of the upper NICE threshold of £30,000 per QALY but this analysis took a conservative approach since it may be that incremental QALYs for spironolactone would have been greater had we been able to control for any placebo effect and had more complete data beyond 24 weeks. When taking into account missing data the ICER was below the upper NICE threshold suggesting spironolactone may be considered cost-effective. However, all analyses show a high degree of uncertainty suggestive of a need for further research to allow conclusions to be drawn.

ACKNOWLEDGEMENTS

We would like to thank all PPI contributors, participants, research and clinical staff, the NIHR Clinical Research Network, and the members of the Trial Steering Committee and Data Monitoring Committee for their support.

The study was developed with support from the UK Dermatology Clinical Trials Network (UK DCTN). The UK DCTN is grateful to the British Association of Dermatologists and the University of Nottingham for financial support of the Network.

The University of Southampton was the research sponsor for this trial.

For peer review only

CONTRIBUTORSHIP STATEMENT

MS, AL, BS, THS, MJR, NF, KST, PL, JN, GG and IM conceived the study idea and initial study design in response to a NIHR HTA call, with later input from KLT, IS, ZE, SR, ML, NP and SP. All authors contributed to the acquisition of data. Specific advice was given by BS on trial design and medical statistics; and THS on health economic evaluation. Economic analyses were conducted by SP and THS. All authors contributed to the interpretation of data and drafting of this paper, led by SP and THS, and approved the final manuscript.

For peer review only - http://bmjopen.bmj.com/site/about/guidelines.xhtml

COMPETING INTERESTS

We declare no support from any organisation other than the NIHR for the submitted work; no financial relationships with any organisations that might have an interest in the submitted work; no other relationships or activities that could appear to have influenced the submitted work.

LH has received consultancy fees from the University of Oxford on an educational grant funded by Pfizer, unrelated to the submitted work. THS was a member of NIHR HTA Efficient Study Designs - 2, HTA Efficient Study Designs Board, HTA End of Life Care and Add-on-Studies, HTA Primary Care Themed Call Board and the HTA Commissioning Board between 2013 to Dec 2019. She is a steering committee member of the UK Dermatology Clinical Trials Network and Chair of the NIHR Research for Patient Benefit Regional Advisory Panel for the East of England. THS had no part in the decision making for funding this study.

FUNDING

This study presents independent research funded by the National Institute for Health and Care Research (NIHR) under its Health Technology Assessment programme (16/13/02). The views expressed are those of the author(s) and not necessarily those of the NHS, the NIHR or the Department of Health and Social Care.

This trial was registered prospectively with the ISRCTN registry (ISRCTN12892056) and EudraCT (2018-003630-33).

DATA SHARING STATEMENT

Consent was not obtained from participants for data sharing but authors will consider reasonable requests to make relevant anonymised participant level data available via the Southampton Clinical Trials Unit Data Sharing Committee.

ETHICS STATEMENT

This study was conducted in compliance with the ethical principles of the Declaration of Helsinki and in compliance with all International Council for Harmonisation Good Clinical Practice guidelines. The study protocol was reviewed and approved by the Institutional Review board and/or Independent Ethics Committee at each participating centre. All participants provided written informed consent.

Ethical approval for the trial was given by Wales Research Ethics Committee (REC) 3 in January 2019 (18/WA/0420).

SUPPORTING INFORMATION

Additional supporting information may be found in the online version of this article at the publisher's website:

Appendix S1: Participant Resource Use Questionnaire

Appendix S2 Supplementary material: further sensitivity and sub-group analyses

Figure S1 Intervention resource use as delivered via secondary care, per trial protocol

For peer review only - http://bmjopen.bmj.com/site/about/guidelines.xhtml

Table S1 Estimates of mean baseline resource use by treatment group (available case data)

Table S2 Estimates of mean change in cost (UK£ 2021/22) including wider costs, by treatment group

Table S3 Cost utility analyses and cost-effectiveness analyses results, for additional sensitivity analyses and sub-group analysis

Table S4 Mean (Standard Deviation) Cost and Cost Difference (95% Confidence Interval) Per Patient up to 25-–52 weeks for the Intervention arm compared to usual care arm (in 2021 UK pounds sterling)

REFERENCES

1 Purdy S, de Berker D. Acne. *BMJ* 2006;**333**:949–53. doi:10.1136/bmj.38987.606701.80

2 Schofield J, Grindlay D, Williams H. Skin conditions in the UK: a Health Care Needs Assessment. University of Nottingham: 2009.

3 Cunliffe WJ. Acne and unemployment. *Br J Dermatol* 1986;**115**:386–386. doi:10.1111/J.1365-2133.1986.TB05757.X

4 Jowett S, Ryan T. Skin disease and handicap: an analysis of the impact of skin conditions. *Soc Sci Med* 1985;**20**:425–9. doi:10.1016/0277-9536(85)90021-8

5 Mavranezouli I, Daly CH, Welton NJ, *et al.* A systematic review and network meta-analysis of topical pharmacological, oral pharmacological, physical and combined treatments for acne vulgaris. *Br J Dermatol* 2022;**187**:639–49. doi:10.1111/BJD.21739

6 Mavranezouli I, Welton NJ, Daly CH, *et al.* Cost-effectiveness of topical pharmacological, oral pharmacological, physical and combined treatments for acne vulgaris. *Clin Exp Dermatol* Published Online First: 30 July 2022. doi:10.1111/ced.15356

7 NICE. Acne vulgaris: management NICE guideline. 2021. www.nice.org.uk/guidance/ng198 (accessed 16 Nov 2022).

8 le Cleach L, Lebrun-Vignes B, Bachelot A, *et al.* Guidelines for the management of acne: recommendations from a French multidisciplinary group. *Br J Dermatol* 2017;**177**:908–13. doi:10.1111/BJD.15843

9 Thiboutot DM, Dréno B, Abanmi A, *et al.* Practical management of acne for clinicians: An international consensus from the Global Alliance to Improve Outcomes in Acne. *J Am Acad Dermatol* 2018;**78**:S1-S23.e1. doi:10.1016/J.JAAD.2017.09.078

10 Zaenglein AL, Pathy AL, Schlosser BJ, *et al.* Guidelines of care for the management of acne vulgaris. *J Am Acad Dermatol* 2016;**74**:945-973.e33. doi:10.1016/J.JAAD.2015.12.037

11 Platt D, Muller I, Sufraz A, *et al.* GPs' perspectives on acne management in primary care: a qualitative interview study. *Br J Gen Pract* 2020;**71**:E78–84. doi:10.3399/BJGP20X713873

For peer review only - http://bmjopen.bmj.com/site/about/guidelines.xhtml

12 Layton AM, Eady EA, Whitehouse H, *et al.* Oral Spironolactone for Acne Vulgaris in Adult Females: A Hybrid Systematic Review. *Am J Clin Dermatol* 2017;**18**:169–91. doi:10.1007/S40257-016-0245-X

13 Barbieri Lab. Spironolactone versus doxycycline for acne: a comparative non-inferiority evaluation (SD-ACNE) research study. https://barbierilab.bwh.harvard.edu/clinical-trial-opportunities/ (accessed 17 Nov 2022).

14 Poinas A, Lemoigne M, Le Naour S, *et al.* FASCE, the benefit of spironolactone for treating acne in women: study protocol for a randomized double-blind trial. *Trials* 2020;**21**:571. doi:10.1186/S13063-020-04432-W

15 Renz S, Chinnery F, Stuart B, *et al.* Spironolactone for adult female acne (SAFA): protocol for a double-blind, placebo-controlled, phase III randomised study of spironolactone as systemic therapy for acne in adult women. *BMJ Open* 2021;**11**:e053876. doi:10.1136/BMJOPEN-2021-053876

16 Santer M, Lawrence M, Renz S, *et al.* Effectiveness of spironolactone for women with acne vulgaris (SAFA) in England and Wales: pragmatic, multicentre, phase 3, double blind, randomised controlled trial. *BMJ* 2023;**BMJ-2022-074349**:e074349. doi:10.1136/bmj-2022-074349

17 Drummond M, Sculpher M. Common methodological flaws in economic evaluations. *Med Care* 2005;**43**:5–14. doi:10.1097/01.mlr.0000170001.10393.b7

18 Thiede RM, Rastogi S, Nardone B, *et al.* Hyperkalemia in women with acne exposed to oral spironolactone: A retrospective study from the RADAR (Research on Adverse Drug Events and Reports) program. *Int J Womens Dermatol* 2019;**5**:155–7. doi:10.1016/J.IJWD.2019.04.024

19 Plovanich M, YuWeng Q, Mostaghimi A. Low Usefulness of Potassium Monitoring Among Healthy Young Women Taking Spironolactone for Acne. *JAMA Dermatol* 2015;**151**:941–4. doi:10.1001/JAMADERMATOL.2015.34

20 Wang Y, Lipner SR. Retrospective analysis of adverse events with spironolactone in females reported to the United States Food and Drug Administration. *Int J Womens Dermatol* 2020;**6**:272. doi:10.1016/J.IJWD.2020.05.002

21 NHS Business Service Authority. Prescription Cost Analysis - England 2020/21. 2021. https://www.nhsbsa.nhs.uk/statistical-collections/prescription-cost-analysis-england/prescription-cost-analysis-england-202021 (accessed 30 Nov 2022).

22 Herdman M, Gudex C, Lloyd A, *et al.* Development and preliminary testing of the new five-level version of EQ-5D (EQ-5D-5L). *Qual Life Res* 2011;**20**:1727–36. doi:10.1007/s11136-011-9903-x

23 van Hout B, Janssen MF, Feng YS, *et al.* Interim scoring for the EQ-5D-5L: Mapping the EQ-5D-5L to EQ-5D-3L value sets. *Value in Health* 2012;**15**:708–15. doi:10.1016/j.jval.2012.02.008

For peer review only

24 NICE. Guide to the methods of technology appraisal 2013. 2013. doi:10.2165/00019053-200826090-00002

25 Manca A, Hawkins N, Sculpher MJ. Estimating mean QALYs in trial-based cost-effectiveness analysis: The importance of controlling for baseline utility. *Health Econ* 2005;**14**:487–96. doi:10.1002/hec.944

26 Fehnel SE, McLeod LD, Brandman J, *et al.* Responsiveness of the Acne-Specific Quality of Life Questionnaire (Acne-QoL) to treatment for acne vulgaris in placebo-controlled clinical trials. *Qual Life Res* 2002;**11**:809–16. doi:10.1023/A:1020880005846

27 Martin AR, Lookingbill DP, Botek A, *et al.* Health-related quality of life among patients with facial acne -- assessment of a new acne-specific questionnaire. *Clin Exp Dermatol* 2001;**26**:380–5. doi:10.1046/J.1365-2230.2001.00839.X

28 Willan AR, Briggs AH, Hoch JS. Regression methods for covariate adjustment and subgroup analysis for non-censored cost-effectiveness data. *Health Econ* 2004;**13**:461–75. doi:10.1002/hec.843

29 Drummond M, Sculpher M, Claxton K, *et al. Methods for the economic evaluation of health care programmes*. 4th ed. Oxford, UK: : Oxford University Press 2015.

30 Ramsey SD, Willke RJ, Glick H, *et al.* Cost-effectiveness analysis alongside clinical trials II - An ISPOR good research practices task force report. *Value in Health* 2015;**18**:161–72. doi:10.1016/j.jval.2015.02.001

31 Faria R, Gomes M, Epstein D, *et al.* A Guide to Handling Missing Data in Cost-Effectiveness Analysis Conducted Within Randomised Controlled Trials. *Pharmacoeconomics* 2014;**32**:1157–70. doi:10.1007/s40273-014-0193-3

32 Xu J, Mavranezouli I, Kuznetsov L, *et al.* Management of acne vulgaris: summary of NICE guidance. *BMJ* 2021;**374**:n1800. doi:10.1136/bmj.n1800

33 Leurent B, Gomes M, Faria R, *et al.* Sensitivity Analysis for Not-at-Random Missing Data in Trial-Based Cost-Effectiveness Analysis: A Tutorial. *Pharmacoeconomics* 2018;**36**:889–901. doi:10.1007/s40273-018-0650-5

34 Thorn JC, Davies CF, Brookes ST, *et al.* Content of Health Economics Analysis Plans (HEAPs) for Trial-Based Economic Evaluations: Expert Delphi Consensus Survey. *Value in Health* 2021;**24**:539–47. doi:10.1016/j.jval.2020.10.002

35 Yang Y, Brazier J, Longworth L. EQ-5D in skin conditions: an assessment of validity and responsiveness. *European Journal of Health Economics* 2015;**16**:927–39. doi:10.1007/s10198-014-0638-9

36 Klassen AF, Newton JN, Mallon E. Measuring quality of life in people referred for specialist care of acne: Comparing generic and disease-specific measures. *J Am Acad Dermatol* 2000;**43**:229–33. doi:10.1067/mjd.2000.105507

For peer review only

37 Jones K, Burns A. Unit Costs of Health and Social Care 2021. PSSRU. 2021.https://www.pssru.ac.uk/project-pages/unit-costs/unit-costs-of-health-and-social-care-2021/ (accessed 5 Dec 2022).

38 NHS England. 2019/2020 National Cost Collection Data Publication. London: 2021. https://www.england.nhs.uk/publication/2019-20-national-cost-collection-data-publication/ (accessed 5 Dec 2022).

39 Curtis L, Burns A. *Unit costs of health and social care 2015*. Personal Social Services Research Unit, University of Kent, Canterbury 2015.

40 UKHCA. UKHCA Commisioning Survey 2012: Care is not a Commodity. 2012. file://ueahome/eresfmh4/jry14qdu/data/Downloads/UKHCACommissioningSurvey2012.pdf (accessed 7 Aug 2023).

41 Brown R. PSNC Pharmacy Advice Audit 2021. 2021. https://cpe.org.uk/wp-content/uploads/2021/05/PSNC-Pharmacy-Advice-Audit-2021-Report.pdf (accessed 7 Aug 2023).

42 Office for National Statistics. Annual Survey of Hours and Earnings time series of selected estimates. 2021.https://www.ons.gov.uk/employmentandlabourmarket/peopleinwork/earningsandworkinghours/datasets/ashe1997to2015selectedestimates (accessed 5 Dec 2022).

FIGURES

Figure 1 | Intervention resource use as per standard treatment with spironolactone (base-case)

Figure 2 | Cost Effectiveness Acceptability Curve (CEAC), complete case analysis, adjusted and unadjusted QALYs

For peer review only

For peer review only - http://bmjopen.bmj.com/site/about/guidelines.xhtml

TABLES

Table 1 | Unit costs (UK£ sterling, 2020/21 financial year)

Cost Item	Unit Cost (£)	Unit	Source, assumptions
**Intervention**			
Spironolactone with dose escalation	£49.37	Total	Prescription Cost Analysis 2021.[21]
GP visit related to intervention	£33.00	Total	PSSRU Unit costs 2021.[37]
Blood test for renal function (eGFR) and potassium level (K serum)	£5.22	Total	National Cost Collection 2020.[38]*
**Medication costs**	Mean cost per quantity		
Topical preparations for acne	£0.96	gram/ml	Prescription Cost Analysis 2021.[21]<br>Mean across all medications in each medication type. Weighted averages taken where listed >1x.<br>Weighted average for estimating oral antibiotic control for SA (see table 3). Assumes 1x100 mg (doxycycline)/408 mg (lymecycline) per day for 24 weeks.
Other topical preparation	£0.03	gram/ml	
Oral contraceptives	£0.08	tablet	
Oral antibiotics	£0.22	capsule/tablet	
Anti-depressants	£0.20	capsule/tablet	
Analgesics	£0.04	capsule/tablet	
PCOS/diabetes medication	£0.03	tablet	
Other medications	£0.40	various	
Doxycycline/lymecycline weighted average	£0.25	Capsule	
**Community-based HCP contacts**			
GP visit unrelated to intervention	£33.00	Visit	PSSRU Unit costs 2021.[37]
Practice Nurse	£14.13	Visit	PSSRU Unit costs 2021 & 2015.[37,39]
NHS Walk-in centre	£71.99	Visit	National Cost Collection 2020.[38] Weighted average of all community health services.*
Community dermatology service	£121.01	Visit	National Cost Collection 2020.[38]*
Healthcare assistant	£14.44	Visit	PSSRU Unit Costs 2021[37] & UKHCA Commissioning Survey 2012.[40]
Pharmacist	£6.99	Visit	PSSRU Unit costs 2021 & 2015[37,39] & PSNC Pharmacy Advice Audit 2021.[41]
Physiotherapist	£66.82	Visit	National Cost Collection 2020.[38]*
Dietician	£82.46	Visit	National Cost Collection 2020.[38]*
Other (community)	£33.00	Visit	PSSRU Unit costs 2021. Used most common visit: GP visit.[37]
**Hospital out-patient contacts**			
Dermatologist	£128.25	Visit	National Cost Collection 2020.[38]*

For peer review only

For peer review only - http://bmjopen.bmj.com/site/about/guidelines.xhtml

Dermatology Nurse	£100.71	Visit	National Cost Collection 2020.[38]*
Ear, nose and throat (ENT)	£116.11	Visit	National Cost Collection 2020.[38]*
Interventional radiology	£137.64	Visit	National Cost Collection 2020.[38]*
Trauma and orthopaedics	£125.67	Visit	National Cost Collection 2020.[38]*
Respiratory medicine	£161.07	Visit	National Cost Collection 2020.[38]*
Other (out-patient)	£137.10	Visit	National Cost Collection 2020.[38]*
**Hospital admission**			
Accident and emergency	£182.28	Visit	National Cost Collection 2020. Index/Accident & Emergency.[38]*
**Wider costs**			
Personal out-of-pocket expenses	Various	Per item	Participant reported.
Lost work time	£18.01	Hour	ONS 2021.[42] Mean hourly earnings, excluding overtime (£).

SA, sensitivity analysis; ONS, Office for National Statistics; PSSRU, Personal Social Services Research Unit.

*Inflated to 2021 prices as per NHSCII Pay & Prices.[37]

For peer review only - http://bmjopen.bmj.com/site/about/guidelines.xhtml

Table 2 | Estimates of mean change in resource use and cost (UK£ 2021/22) and mean utility and QALY gain by treatment group (based on available case data)

**Resource**	**Spironolactone (N=201)**		**No active systemic treatment (N=209)**		**Mean difference**
	**Mean (n)**	**SD**	**Mean (n)**	**SD**	**(95% CI)**
Resource use over 24-week period:					
Spironolactone (number)	294 (201)	0	0 (209)	0	-
GP visits related to intervention (no. of visits)*	2.06 (201)	0.34	0 (209)	0	-
Blood tests – renal function (eGFR) and potassium level (number)	1.06 (201)	0.34	0 (209)	0	-
Total community-based HCP visits (number)	0.15 (150)	0.51	0.10 (124)	0.43	0.05 (-0.06 to 0.16)
Total hospital contacts (number)	0.06 (132)	0.30	0.05 (115)	0.26	0.01 (-0.06 to 0.08)
All prescription medications (number)	11.42 (147)	29.65	23.36 (124)	96.80	-11.94 (-28.51 to 4.63)
Total out-of-pocket items	3.59 (131)	5.96	4.49 (113)	6.67	-0.90 (-2.49 to 0.69)
Lost patient work time (number reporting)	0.00 (186)	0.00	0.02 (191)	0.144	-0.02 (-0.04 to -0.00)
Lost carer work time (number reporting)	0.01 (185)	0.07	0.02 (190)	0.144	-0.02 (-0.04 to 0.01)
Costs over 24-week period (UK£2021/22):					
All intervention costs	122.87 (201)	13.04	0 (209)	0	122.87 (121.09 to 124.64)
All community-based HCP costs	6.28 (150)	24.83	3.75 (124)	16.46	2.53 (-2.60 to 7.66)
All hospital contact costs	7.28 (132)	36.42	5.73 (115)	28.09	1.55 (-6.70 to 9.79)
All prescription medication costs	4.37 (147)	11.77	5.91 (124)	18.93	-1.54 (-5.25 to 2.17)
**Total costs**	**141.99 (128)**	**57.90**	**15.64 (110)**	**45.62**	**126.35 (112.88 to 139.82)**
**Total costs excluding intervention**	**19.61 (128)**	**56.65**	**15.64 (110)**	**45.62**	**3.98 (-9.30 to 17.26)**
Total out-of-pocket costs	69.41 (139)	113.05	82.57 (120)	148.60	-13.15 (-45.23 to 18.92)
Lost patient and carer productivity	27.87 (177)	354.76	15.95 (179)	183.54	11.93 (-46.86 to 70.71)
**Total costs (wider perspective)**	**252.67 (113)**	**490.19**	**93.53 (100)**	**144.02**	**159.14 (58.86 to 259.41)**
EQ-5D score (CUA)					
Baseline	0.887 (200)	0.148	0.860 (209)	0.200	0.027 (-0.008 to 0.061)
6 weeks	0.894 (176)	0.135	0.863 (179)	0.168	0.031 (-0.001 to 0.063)
12 weeks	0.904 (174)	0.138	0.877 (166)	0.177	0.027 (-0.007 to 0.061)
24 weeks	0.909 (163)	0.153	0.890 (136)	0.180	0.019 (-0.019 to 0.057)
**Total QALY score over 24 weeks**	**0.417 (162)**	**0.058**	**0.404 (136)**	**0.079**	**0.013 (-0.002 to 0.029)**
Acne-QoL symptom sub-scale score (CEA)					
Baseline	13.22 (201)	4.94	12.87 (209)	4.55	0.35 (-0.57 to 1.27)
6 weeks	16.97 (176)	5.72	15.65 (179)	5.69	1.32 (0.13 to 2.51)
12 weeks	19.21 (176)	6.12	17.76 (166)	5.58	1.45 (0.20 to 2.69)
24 weeks	21.22 (163)	5.86	17.39 (136)	5.80	3.83 (2.49 to 5.16)
**Change at 24-weeks from baseline**	**8.15 (163)**	**6.12**	**4.46 (136)**	**6.34**	**3.68 (2.26 to 5.11)**

*Assumes that if spironolactone is found effective it would be prescribed in primary care.

For peer review only - http://bmjopen.bmj.com/site/about/guidelines.xhtml

Table 3 | Cost utility analyses and cost-effectiveness analyses results, including sensitivity analyses and sub-group analysis

**CUA Analysis (N s, N p)**	**Incremental cost (95% CI)**	**Incremental QALYs (95% CI)**	**ICER**	**CEAC at £20,000 (£30,000) threshold***
Base-case^, CCA, adjusted (118,101)	125.36 (111.13 to 139.58)	0.0019 (-0.0096 to 0.0133)	£67,191	23% (35%)
Base-case^, CCA, unadjusted (126,109)	125.53 (112.15 to 138.91)	0.0036 (-0.0117 to 0.0189)	£34,770	37% (47%)
SA1^, Multiple imputation, adjusted (201,209)	119.78 (107.99 to 131.57)	0.0043 (-0.0041 to 0.0127)	£27,879	35% (53%)
SA2: Secondary care delivery, CCA, adjusted (118,101)	265.67 (250.52 to 280.82)	0.0019 (-0.0096 to 0.0133)	£141,955	3% (12%)
SA3a, oral antibiotic control, CCA, adjusted (118,101)	17.11 (2.88 to 31.33)	Threshold analysis value†: 0.00057		
SA3b, oral antibiotic control, MI, adjusted (201, 209)	11.53 (-0.26 to 23.32)	Threshold analysis value†: 0.00038		
SA4a: Wider perspective, CCA, adjusted (97,85)	102.07 (64.21 to 139.92)	-0.0027 (-0.0139 to 0.0085)	Dominated	9% (15%)
SA4b: Wider perspective, MI, adjusted (201,209)	133.25 (72.52 to 193.93)	0.0044 (-0.0041 to 0.0129)	£30,249	31% (50%)
**CEA Analysis (N s, N p)**	**Incremental cost (95% CI)**	**Incremental Acne-QoL symptom (95% CI)**	**Incremental cost per unit change**	-
Secondary analysis^, CCA, adjusted: (119,102)	126.57 (112.35 to 140.78)	3.31 (1.90 to 4.72)	£38.21	-
Secondary analysis^, CCA, unadjusted (127,110)	126.52 (113.00 to 140.04)	3.52 (1.94 to 5.11)	£35.91	-

^ comparing spironolactone plus routine topical treatment to no active systemic treatment plus routine topical treatment; 95% CI=95% confidence interval; ICER =incremental cost-effectiveness ratio; N s / N p =Number randomised to spironolactone / Placebo who were included in the analysis; CCA = complete case analysis; SA refers to the different sensitivity analyses described in the Methods; QALY=Quality Adjusted Life Years; *probability of being cost-effective at a the threshold (λ) of £20,000 and £30,000 per QALY. Adjusted analyses, adjusted for stratification variables (centre, baseline severity [IGA<3 vs. ≥3]) and baseline variables (Acne QoL symptom subscale score, use of topical treatments, utility score based on EQ-5D, total costs). †Threshold analysis conducted using a £30,000 threshold, as described in the methods. The value given represents the incremental QALY benefit below which spironolactone compared with oral antibiotic would switch from cost-effective to not cost-effective.

For peer review only

For peer review only - http://bmjopen.bmj.com/site/about/guidelines.xhtml

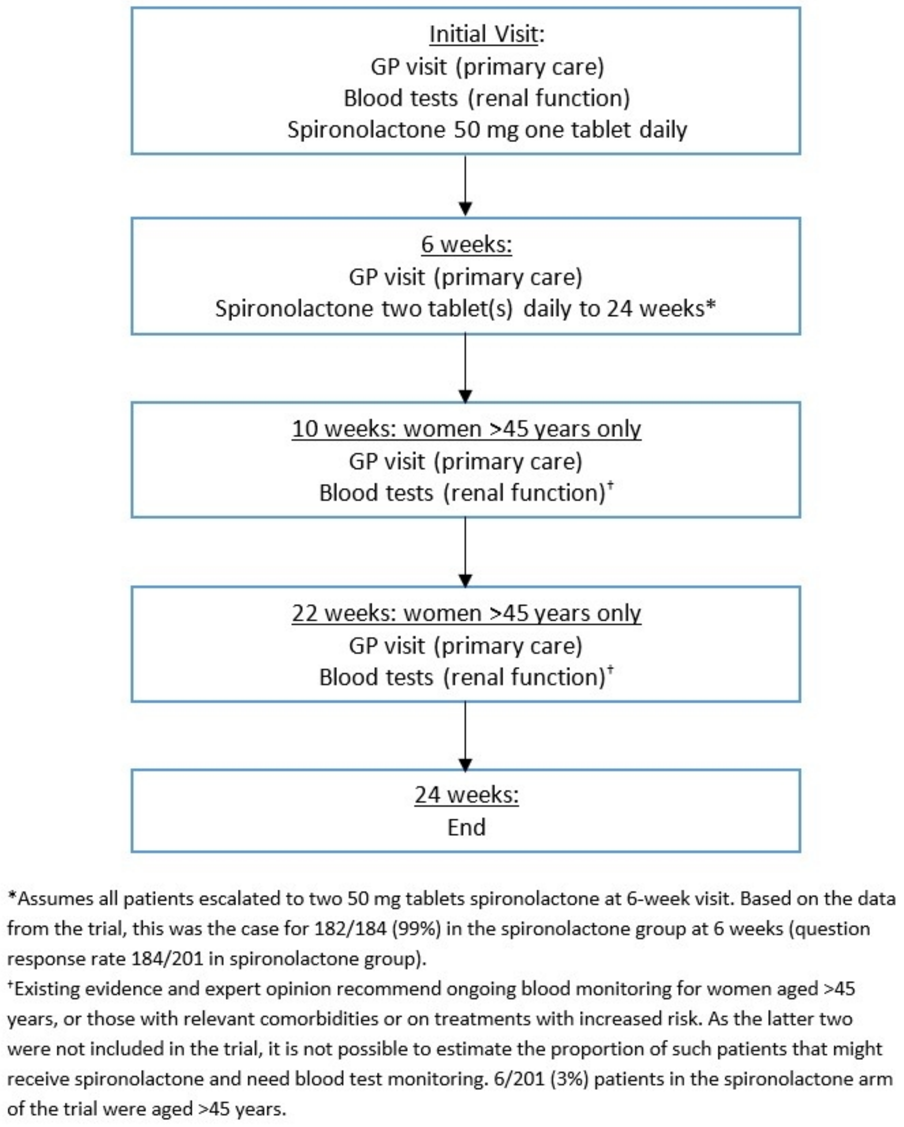

Figure 1: Intervention resource use as per standard treatment with spironolactone (base-case)

190x232mm (96 x 96 DPI)

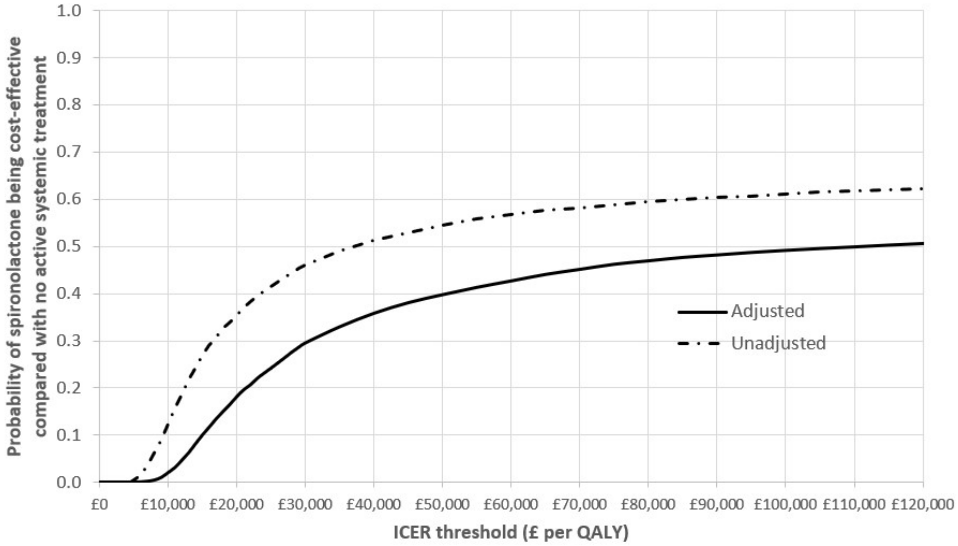

Figure 2: Cost Effectiveness Acceptability Curve (CEAC), complete case analysis, adjusted and unadjusted QALYs

246x145mm (96 x 96 DPI)

SUPPORTING INFORMATION

APPENDIX S1: PARTICIPANT RESOURCE USE QUESTIONNAIRE

The following information is presented in addition to the main paper, "Cost-effectiveness of Spironolactone for Adult Female Acne (SAFA): Economic evaluation alongside a randomised controlled trial", published in XXXX.

The example given below is taken from the SAFA 6-week questionnaire. These questions were part of a wider questionnaire used at 6 weeks.

For peer review only

Participant's initials: | | | | Participant's study identifier: | | | | | | | | |

SAFA 6 week Questionnaire – Participant

Services received

These questions are about your health and care needs. In the **last 6 weeks** what publically provided services (i.e. those you do not have to pay for out of your own pocket) have you received because of your acne?

If you are unsure, please put in your best estimate.

Question 1: Community-based NHS services

1a. In the **last 6 weeks** have you seen any community-based health professionals (e.g. GP, practice nurse, dietician etc) because of your acne?

☐ Yes ☐ No, if 'No' please go to question 2

Have you seen any of the following health professionals in the **last 6 weeks**? If 'Yes', please tell us how many times. There is space for you to name other professionals you have seen via the NHS and how many time you have visited them. If you did not see any other professionals please tick 'No' in the "Other" rows.

General Practitioner	☐ No	☐ yes	If yes, how many times?	___________
Practice nurse	☐ No	☐ Yes	If yes, how many times?	___________
Health care assistant	☐ No	☐ Yes	If yes, how many times?	___________
NHS Walk in centre	☐ No	☐ Yes	If yes, how many times?	___________
Community dermatology service	☐ No	☐ Yes	If yes, how many times?	___________
Other, please specify: ___________________________	☐ No	☐ Yes	If yes, how many times?	___________
Other, please specify: ___________________________	☐ No	☐ Yes	If yes, how many times?	___________
Other, please specify: ___________________________	☐ No	☐ Yes	If yes, how many times?	___________

For peer review only - http://bmjopen.bmj.com/site/about/guidelines.xhtml
Funded by NIHR

Participant's initials: ☐☐☐ Participant's study identifier: ☐☐☐☐☐☐☐☐

Question 2: Medication

2. In the **last 6 weeks** have you been **prescribed** any medications because of your acne? (Please include anything that you feel is related to your acne, for example if you take anti-depressants and your depression is mainly because of your acne you would include this).

☐ Yes ☐ No, if 'No' please go to question 3a

If 'Yes', please give the name of the medication, the strength, and size of the item.

Name of medication (item)	Strength	Unit	Number of items	Type of item (e.g. pack, bottle, tube, etc)	Number in item	Size of item
Example 1: Epiduo Gel	2.5	%	2	tubes	12	grams per tube
Example 2: Tetracyclin	250	mg	1	pack	28	tablets per pack

Question 3: Hospital-based services

3a. **In the last 6 weeks** have you visited a hospital as an outpatient because of your acne or side effects from treatment for your acne?

☐ Yes ☐ No, if 'No' please go to question 3b

If 'Yes', for **each outpatient visit** you had at the hospital as a result of your acne, please tell us which health professional you saw and how many times. Please enter '0' if you did not visit the health professional or in 'Other' if there were no other visits.

Please do not include visits with any professionals that took place outside of the hospital. These should be included in question 1 above. Please do not include visits made as part of this study in your answers below.

For peer review only - http://bmjopen.bmj.com/site/about/guidelines.xhtml

Funded by NIHR

Participant's initials: | | | |

Participant's study identifier: | | | | | | | | |

Health professional you saw<br>*(If unknown, please write the department in which you saw them)*	Number of outpatient visits
Example: Dermatology nurse	*2* visits
Dermatologist	visits
Dermatology nurse	visits
Other, please specify:	visits
Other, please specify:	visits
Other, please specify:	visits

3b. Did you attend Accident and Emergency Services in the **last 6 weeks** because of your acne or side effects from treatment for your acne?

☐ Yes ☐ No, if 'No' please go to question 3c

If 'Yes', how many visits in the last 6 weeks: ______________________________

3c. In **the last 6 weeks**, have you been admitted to hospital as an inpatient as a result of your acne or side effects from treatment for your acne?

☐ Yes ☐ No, if 'No' please go to question 4

If 'Yes', for **each inpatient** visit you have had, please tell us the type of ward you were admitted to and for how many nights.

Please include any day case procedures

Visit number	The type of department or ward or reason for admission	Duration of each stay<br>(number of nights)
Example	*Dermatology*	*2* nights
1		nights
2		nights

For peer review only - http://bmjopen.bmj.com/site/about/guidelines.xhtml

Funded by NIHR

SAFA
Spironolactone for Adult Female Acne

Participant's initials: | | | |

Participant's study identifier: | | | | | | | | |

Question 4: Other services

4. In the **last 6 weeks** have you received any other publically provided services because of your acne?

☐ Yes ☐ No, if 'No' please go to question 5a

If 'Yes', please give details including type and how many times received:

Details of service	Type of service	Number of times received

Costs incurred by yourself or family

These next few questions are about the costs incurred by you and your family/friends because of your acne.

Question 5: Personal Costs

5a. In the **last 6 weeks** have you or your family/friends incurred any other costs because of your acne? Please do not include visits made as part of this study in your answers below.

☐ Yes ☐ No, if 'No' please go to question 5b

If 'Yes', please give the details below and the approximate cost of items purchased as a result of your acne.

Item	Number of items or visits	Overall cost
Example: Homeopath	*2 visits*	*£80 (2 x £40)*
Complementary therapists		
Non-prescribed medication		
Travel costs to health care appointments		
Parking costs at health care appointments		
Cosmetic and skin care products		
Other, please specify:		

For peer review only - http://bmjopen.bmj.com/site/about/guidelines.xhtml

Funded by NIHR

Participant's initials: | | | |

Participant's study identifier: | | | | | | | | |

Other, please specify:		
Other, please specify:		

5b. What is your current **primary occupation**? Please tick one:

☐ Paid employment ☐ self-employment ☐ Voluntary work ☐ Education/studying

☐ None of the above (i.e. retired, unemployed)

In the **last 6 weeks** has your acne had an impact on your primary occupation?

☐ Yes ☐ No, if 'No' please go to question 5c.

If 'Yes', please fill in each row of the table below **about how your acne has affected your primary occupation** in the last 6 weeks. This asks only about your acne, so if, for example, you reduced your hours worked to look after a dependent please do not put this in this table. Please do not include visits made as part of this study in your answers below.

I have had to take leave	☐ No	☐ Yes	If yes, how much leave have you taken in the last 6 weeks?<br>_____weeks ________days _______hours<br>If in paid employment or self-employment, was this paid leave?<br>☐ Yes ☐ No ☐ Mixture of paid and unpaid<br>If a mixture of paid and unpaid leave, how much of the leave was paid leave?<br>_____weeks ________days _______hours
I have reduced the hours I undertake my primary occupation each week	☐ No	☐ Yes	If yes, how many hours per week did you used to undertake?<br>How many hours per week do you undertake now?<br>How long ago did this change:<br>_____weeks ________days _______hours

For peer review only - http://bmjopen.bmj.com/site/about/guidelines.xhtml

Funded by NIHR

Participant's initials: | | | | Participant's study identifier: | | | | | | | | |

I have increased the hours I undertake my primary occupation each week	☐ No	☐ Yes	If yes, how many hours per week did you used to undertake?<br><br>How many hours per week do you undertake now?<br><br>How long ago did this change:<br>_____weeks ________days _______hours
I have completely stopped my primary occupation and will not be going back to it	☐ No	☐ Yes	How long ago did this change:<br>_____weeks ________days _______hours
I have changed my role within my primary occupation	☐ No	☐ Yes	If yes, what was your old role title:<br><br>What is your new role title:<br><br>How long ago did this change:<br>_____weeks ________days _______hours

5c. Have you had a family member or friend who has had to take time off paid work to accompany you to health care appointments related to your acne?

☐ Yes ☐ No, if 'No' please go to question 5d

If yes, how much leave have they had to take in the last 6 weeks to accompany you to appointments related to your acne?

________hours

5d. **Support outside of official services** (For example, charity support groups such as The Acne and Rosacea Association, helplines etc)

In the **last 6 weeks**, have you received support or attended support groups?

☐ Yes ☐ No

If 'Yes', please list what support you have accessed and state whether you incurred any costs as a result (e.g. membership fee, participation fee, telephone cost etc)

Funded by NIHR

For peer review only - http://bmjopen.bmj.com/site/about/guidelines.xhtml

Participant's initials: | | | |

Participant's study identifier: | | | | | | | | |

Type of Support	Cost Incurred (£)
	£
	£
	£

Thank you for completing this questionnaire.

For peer review only

For peer review only - http://bmjopen.bmj.com/site/about/guidelines.xhtml
Funded by NIHR

SUPPORTING INFORMATION

APPENDIX S2: FURTHER SENSITIVITY AND SUBGROUP ANALYSES

The following information is presented in addition to the main paper, "Cost-effectiveness of Spironolactone for Adult Female Acne (SAFA): Economic evaluation alongside a randomised controlled trial", published in The BMJ.[1] In addition to the sensitivity analyses presented in the main paper, a further two sensitivity analyses and a sub-group analysis were agreed before analysis and conducted to explore key uncertainties around the parameters of the economic evaluation. The details of these are outlined below.

Baseline Resource use
Table S1 presents the levels of resource use, at baseline, prior to randomisation (Table S1).

Sensitivity analysis: costing the intervention as per the SAFA trial protocol

Figure S1 describes the per protocol intervention resource use, undertaken in the trial and used to inform sensitivity analysis 2 (SA2). Subgroup analysis by age
A single sub-group analysis was undertaken for age (categorised as below 25 years and 25 years and over) as the clinical analysis found age significantly interacts with the outcome.[1]

The ICER was £263,871 per QALY for women under 25 years compared to £19,994 for women over 25 years of age (see Table S2). This result suggests that spironolactone is likely to be cost effective for women aged over 25 years. Whilst this finding is in line with the clinical findings, it ought to be interpreted with caution given the small sample sizes necessitated by splitting the dataset into subgroups combined with missing data.

Costs and outcomes over 52 weeks
Data was also collected beyond the treatment period (24 weeks) for up to 52 weeks. Response rates were, however, significantly lower at this time point, with 58% of participants missing EQ-5D data and 93% missing resource use data (see Supplementary Table S3). It is difficult to draw conclusions from these data, but incremental QALYs over 52 weeks was 0.0644 (95%CI 0.0093 to 0.1194) and incremental cost (NHS perspective) (see Supplementary Table S4) over the same period was £95.44 (95% CI 8.29 to 182.70).

Reference:

1. Santer M, Lawrence M, Renz S, *et al.* Effectiveness of spironolactone for women with acne vulgaris (SAFA) in England and Wales: pragmatic, multicentre, phase 3, double blind, randomised controlled trial. *BMJ* 2023;**BMJ-2022-074349**:e074349. doi:10.1136/bmj-2022-074349

SUPPLEMENTARY FIGURES

Supplementary Figure S1 | Intervention resource use as delivered via secondary care per trial protocol

For peer review only

SUPPLEMENTARY TABLES

Supplementary Table S1 | Estimates of mean baseline resource use by treatment group (available case data)

Resource	Spironolactone (N=201)		No active systemic treatment (N=209)		Mean difference
	Mean (n)	Std dev	Mean (n)	Std dev	(95% CI)
Total community-based HCP visits	0.27 (200)	0.616	0.225 (209)	0.590	0.045 (-0.072 to 0.162)
Total hospital contacts	0.119 (193)	0.446	0.095 (200)	0.396	0.024 (-0.059 to 0.108)
All medications – quantity (number)	11.711 (201)	46.065	7.903 (206)	21.570	3.809 (-3.174 to 10.791)
Total out-of-pocket items	2.027 (188)	2.735	1.939 (196)	2.438	0.088 (-0.432 to 0.607)
Lost patient work time (number reporting)	0.020 (197)	0.141	0.034 (205)	0.182	-0.014 (-0.046 to 0.018)
Lost carer work time (number reporting)	0.015 (194)	0.124	0.030 (203)	0.170	-0.014 (-0.044 to 0.015)

For peer review only

Supplementary Table S2 | Cost utility analyses and cost-effectiveness analyses results, for additional sub-group analysis

CUA Analysis (N s, N p)	Incremental cost (95% CI)	Incremental QALYs (95% CI)	ICER	CEAC at £20,000 (£30,000) threshold*
Sub-group analysis: <25 years, CCA, adjusted: (28,29)	108.23 (89.09 to 127.37)	0.0004 (-0.0141 to 0.0150)	£263,871	25% (33%)
Sub-group analysis: ≥25 years, CCA, adjusted: (90,72)	133.06 (114.97 to 151.16)	0.0067 (-0.0079 to 0.0213)	£19,994	50% (62%)

95% CI=95% confidence interval; ICER =incremental cost-effectiveness ratio; N s / N p =Number randomised to spironolactone / Placebo who were included in the analysis; CCA = complete case analysis; SA refers to the different sensitivity analyses described in the Methods; QALY=Quality Adjusted Life Years; *probability of being cost-effective at a the threshold (λ) of £20,000 and £30,000 per QALY. Adjusted analyses, adjusted for stratification variables (centre, baseline severity [IGA<3 vs. ≥3]) and baseline variables (Acne QoL symptom subscale score, use of topical treatments, utility score based on EQ-5D, total costs)

For peer review only

Supplementary Table S3 | 1Proportion of Missing values (%) for key variables

Variable	Spironolactone	No active systemic treatment	Total
**Baseline variables**			
Treatment allocation	0	0	0
Centre	0	0	0
Baseline severity (IGA)	0	0	0
Acne-QoL symptom subscale score at baseline	0	0	0
Use of topical treatments (y/n)	1.00	0.48	0.73
EQ-5D at baseline	0.50	0.00	0.24
Costs at baseline	4.48	5.74	5.12
**Cost variables***			
Costs at 6 weeks	17.91	18.18	18.05
Costs at 12 weeks	14.43	23.44	19.02
Costs at 24 weeks	23.88	38.76	31.46
Costs at 52 weeks	92.54	94.26	93.41
**Outcome variables for health-related quality of life**			
EQ-5D at 6 weeks	12.44	14.35	13.41
EQ-5D at 12 weeks	13.43	20.57	17.07
EQ-5D at 24 weeks	20.40	33.49	27.07
EQ-5D at 52 weeks	54.73	61.72	58.29
**Outcome variables for Acne-related quality of life**			
Acne-QoL at 6 weeks	12.44	14.35	13.41
Acne-QoL at 12 weeks	12.44	20.57	16.59
Acne-QoL at 24 weeks	18.91	34.93	27.07
Acne-QoL at 52 weeks	52.74	61.24	57.07
**Outcomes for cost-utility and cost-effectiveness analyses***			
Total costs (treatment period)	36.32	47.38	41.95
Total QALYS (treatment period)	20.90	33.49	27.32
Change Acne-QoL symptoms (treatment period)	18.91	34.93	27.07

Treatment period = baseline to 24 weeks

*For base-case, i.e. NHS-related costs only

For peer review only

Supplementary Table S4 | Mean (Standard Deviation) Cost and Cost Difference (95% Confidence Interval) Per Patient up to 25-–52 weeks for the Intervention arm compared to usual care arm (in 2021 UK pounds sterling)

Resource	Spironolactone (N=201)		No active systemic treatment (N=209)		Mean difference
	Mean (n)	Std dev	Mean (n)	Std dev	(95% CI)
**Costs**					
All community-based HCP costs	19.64 (16)	33.25	33.24 (13)	42.00	-13.60 (-42.25 to 15.05)
Total hospital contacts cost	17.10 (15)	45.13	9.87 (13)	35.57	7.23 (-24.70 to 39.17)
All medication cost	4.81 (16)	11.23	9.66 (13)	19.41	-4.85 (-16.65 to 6.96)
Total costs (NHS perspective), 25–52 weeks	39.89 (15)	67.47	54.41 (12)	79.00	-14.52 (-72.57 to 43.52)
Total costs (NHS perspective), 0–52 weeks	179.21 (13)	76.99	83.76 (10)	123.54	95.44 (8.29 to 182.60)
**Outcomes**					
52 weeks EQ-5D-5L utility	0.9208 (92)	0.1516	0.8291 (79)	0.2664	0.0918 (0.0274 to 0.1561)
QALYs at 52 weeks	0.9158 (88)	0.1364	0.8515 (74)	0.2154	0.0644 (0.0093 to 0.1194)
52 weeks symptom Acne-QoL	21.634 (95)	6.257	19.963 (81)	5.697	1.671 (-0.122 to 3.464)
Symptom Acne QoL change at 52 weeks compared to baseline	8.613 (95)	7.154	6.951 (81)	6.500	1.663 (-0.385 to 3.710)

For peer review only

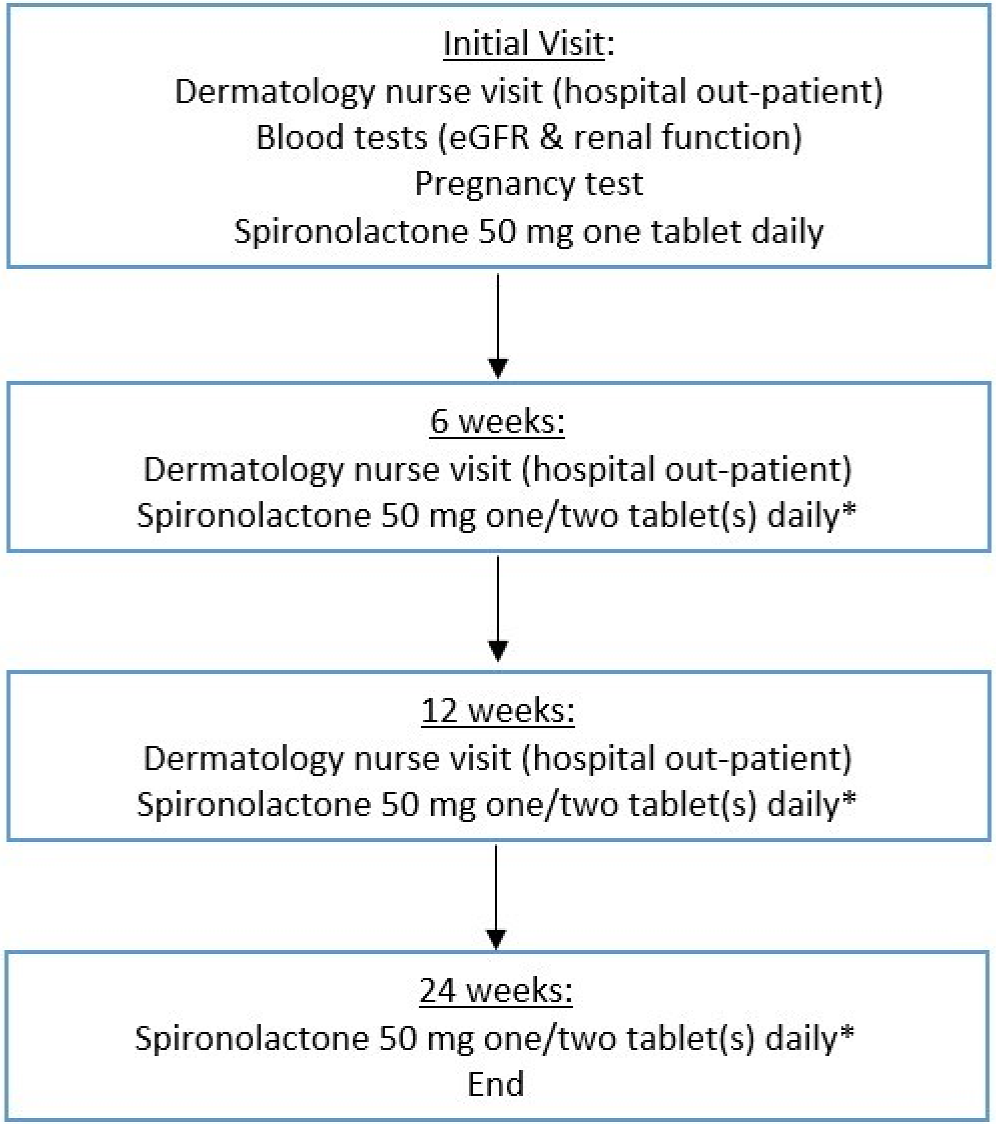

*At 6 weeks, dose was increased to 100 mg/day, assuming treatment was tolerated, which was the case for 182/184 (99%) of available patients in the spironolactone arm of the study

CHEERS 2022 Checklist – For SAFA Trial

	Item	Guidance for Reporting	Reported in section
**TITLE**			
Title	1	Identify the study as an economic evaluation and specify the interventions being compared.	Title and abstract
**ABSTRACT**			
Abstract	2	Provide a structured summary that highlights context, key methods, results and alternative analyses.	Abstract
**INTRODUCTION**			
Background and objectives	3	Give the context for the study, the study question and its practical relevance for decision making in policy or practice.	Introduction
**METHODS**			
Health economic analysis plan	4	Indicate whether a health economic analysis plan was developed and where available.	Methods
Study population	5	Describe characteristics of the study population (such as age range, demographics, socioeconomic, or clinical characteristics).	Results - Participant characteristics
Setting and location	6	Provide relevant contextual information that may influence findings.	Abstract, methods and results
Comparators	7	Describe the interventions or strategies being compared and why chosen.	Methods - Intervention and comparator. Sensitivity analysis 2 and 3.
Perspective	8	State the perspective(s) adopted by the study and why chosen.	Abstract and Methods
Time horizon	9	State the time horizon for the study and why appropriate.	Abstract and methods
Discount rate	10	Report the discount rate(s) and reason chosen.	Methods – Incremental analysis
Selection of outcomes	11	Describe what outcomes were used as the measure(s) of benefit(s) and harm(s).	Methods – measuring outcomes
Measurement of outcomes	12	Describe how outcomes used to capture benefit(s) and harm(s) were measured.	Methods – measuring outcomes
Valuation of outcomes	13	Describe the population and methods used to measure and value outcomes.	Methods – measuring outcomes
Measurement and valuation of resources and costs	14	Describe how costs were valued.	Methods – Measuring costs
Currency, price date, and conversion	15	Report the dates of the estimated resource quantities and unit costs, plus the currency and year of conversion.	Methods – Measuring costs
Rationale and description of model	16	If modelling is used, describe in detail and why used. Report if the model is publicly available and where it can be accessed.	N/A
Analytics and assumptions	17	Describe any methods for analysing or statistically transforming data, any extrapolation methods, and approaches for validating any model used.	Methods – Incremental analysis
Characterizing heterogeneity	18	Describe any methods used for estimating how the results of the study vary for sub-groups.	Supplementary material – Sub-group
Characterizing distributional effects	19	Describe how impacts are distributed across different individuals or adjustments made to reflect priority populations.	Not reported – this is a new item of the checklist which was not published at the time the study was designed.
Characterizing uncertainty	20	Describe methods to characterize any sources of uncertainty in the analysis.	Methods and supplementary analyses – sensitivity analyses
Approach to engagement with patients and others affected by the study	21	Describe any approaches to engage patients or service recipients, the general public, communities, or stakeholders (e.g., clinicians or payers) in the design of the study.	Methods - Patient and public involvement (PPI) and measuring costs
**RESULTS**			
Study parameters	22	Report all analytic inputs (e.g., values, ranges, references) including uncertainty or distributional assumptions.	Results
Summary of main results	23	Report the mean values for the main categories of costs and outcomes of interest and summarise them in the most appropriate overall measure.	Results
Effect of uncertainty	24	Describe how uncertainty about analytic judgments, inputs, or projections affect findings. Report the effect of choice of discount rate and time horizon, if applicable.	Results
Effect of engagement with patients and others affected by the study	25	Report on any difference patient/service recipient, general public, community, or stakeholder involvement made to the approach or findings of the study	Methods - Patient and public involvement (PPI) and measuring costs
**DISCUSSION**			

For peer review only

Study findings, limitations, generalizability, and current knowledge	26	Report key findings, limitations, ethical or equity considerations not captured, and how these could impact patients, policy, or practice.	Discussion
**OTHER RELEVANT INFORMATION**			
Source of funding	27	Describe how the study was funded and any role of the funder in the identification, design, conduct, and reporting of the analysis	Funding sources
Conflicts of interest	28	Report authors conflicts of interest according to journal or International Committee of Medical Journal Editors requirements.	Conflicts of interest

Husereau D, Drummond M, Augustovski F, de Bekker-Grob E, Briggs AH, Carswell C, Caulley L, Chaiyakunapruk N, Greenberg D, Loder E, Mauskopf J, Mullins CD, Petrou S, Pwu RF, Staniszewska S; CHEERS 2022 ISPOR Good Research Practices Task Force. Consolidated Health Economic Evaluation Reporting Standards 2022 (CHEERS 2022) Statement: Updated Reporting Guidance for Health Economic Evaluations. BMJ. 2022;376:e067975.
The checklist is Open Access distributed in accordance with the terms of the Creative Commons Attribution (CC BY 4.0) license, which permits others to distribute, remix, adapt and build upon this work, for commercial use, provided the original work is properly cited. See: http://creativecommons.org/licenses/by/4.0/.

For peer review only
